# Asymmetric [4 + 2] cycloaddition synthesis of 4*H*-chromene derivatives facilitated by group-assisted-purification (GAP) chemistry†

**DOI:** 10.1039/d1ra08323f

**Published:** 2021-12-14

**Authors:** Hossein Rouh, Yao Tang, Sai Zhang, Ahmed I. M. Ali, Kazimierz Surowiec, Daniel Unruh, Guigen Li

**Affiliations:** Department of Chemistry and Biochemistry, Texas Tech University Lubbock Texas 79409-1061 USA guigen.li@ttu.edu

## Abstract

In this work, we present a strategy for the preparation of functionalized 4*H*-chromene derivatives *via* a Cs_2_CO_3_-catalyzed [4 + 2] cycloaddition of enantiopure chiral salicyl *N*-phosphonyl imines with allenoates. Fifteen examples were achieved in excellent yields and diastereoselectivity. The products were purified simply by washing the crude mixture with hexanes following the Group-Assisted Purification (GAP) chemistry/technology to bypass traditional separation methods. The absolute configuration was unambiguously determined by X-ray structure analysis.

## Abstract 2

A new asymmetric method for the synthesis of highly functionalized 4*H*-chromenes was developed *via* Group-Assisted Purification (GAP) chemistry and shown in good to high yield and excellent diastereoselectivity.

## Graphical abstract

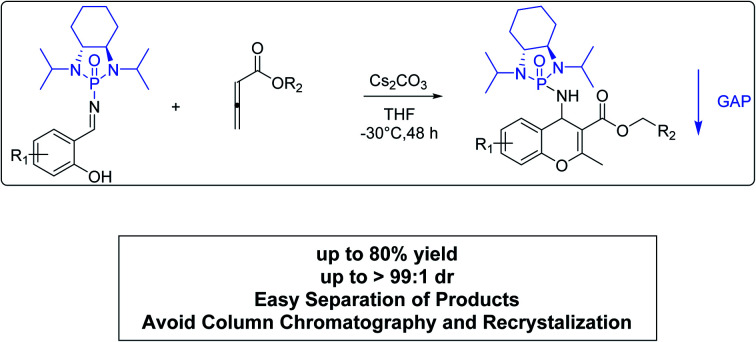

## Introduction

**Fig. 1:**
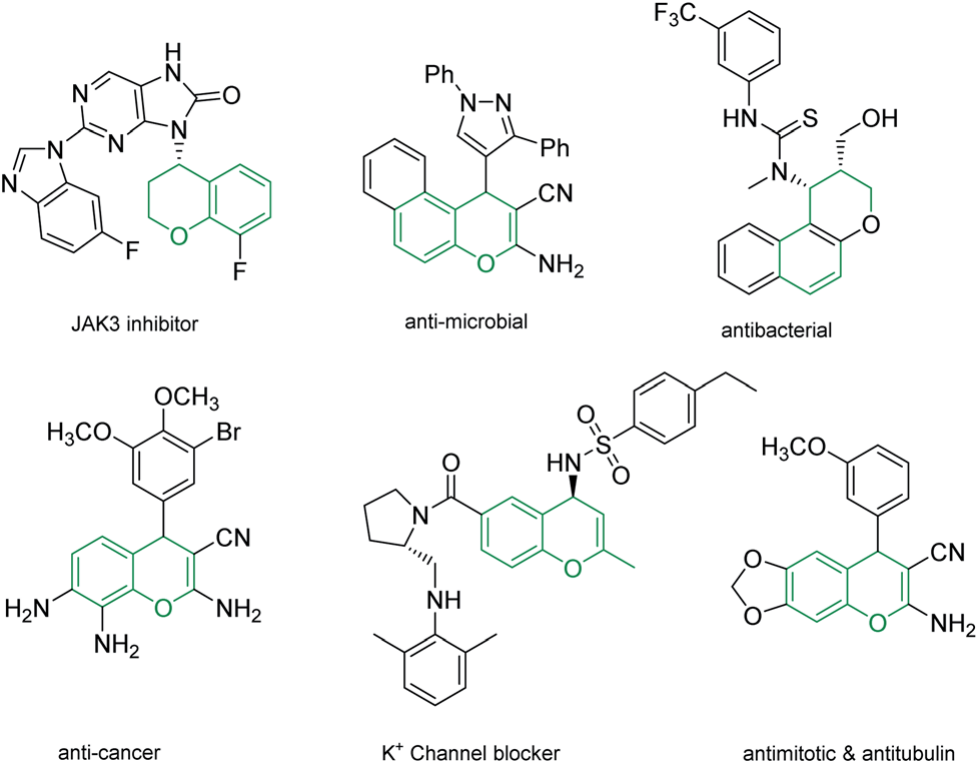
4*H*-Chromenes with biological activity.

4*H*-Chromenes represent an important class of bicyclic oxygenated heterocyclic compounds which are prevalent in a variety of natural products.^1–4^ Molecules exhibiting these motifs demonstrate biological activities such as anticonvulsant,^5^ anti-HIV^6,7^ and antimicrobial^8,9^ and numerous other biological activities (Fig. 1). In addition, chromenes possess anti-cancer properties as they induce apoptosis *via* interaction with tubulin at the binding sites of colchicine which results in the death of cancer cells.^10–13^

**Scheme 1:**
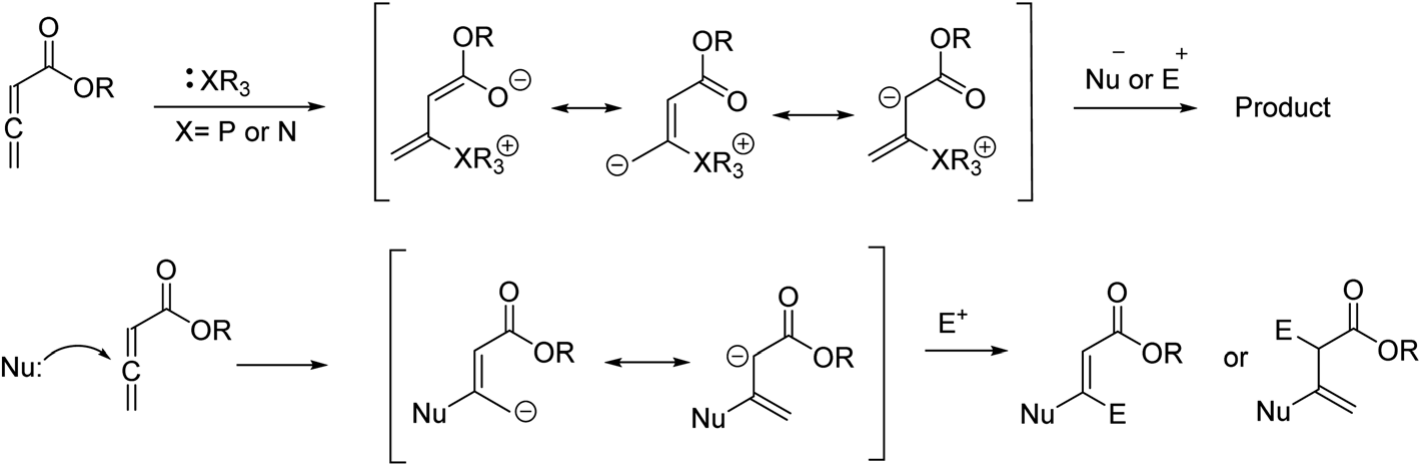
Strategies for the reactions of allenoates with nucleophiles or electrophiles.

In recent years, the chemistry of allenes has attracted significant attention and they are considered reactive substrates with synthetic utility as starting materials from which to prepare complex molecules *via* cycloaddition reactions.^14–20^ The presence of electron withdrawing or electron donating groups on the allene moiety can induce electronic effects and drive allenes to react as either electrophiles or nucleophiles.^21–24^ The reactivity of allenes is contingent upon their ability to form zwitterionic intermediates in the presence of nitrogenous/phosphorous-based or nucleophilic Lewis base catalysts.^25–33^ In addition, it has been reported that allenes can react with nucleophilic compounds in the presence of carbonate catalysis (Scheme 1).^34–36^

**Scheme 2:**
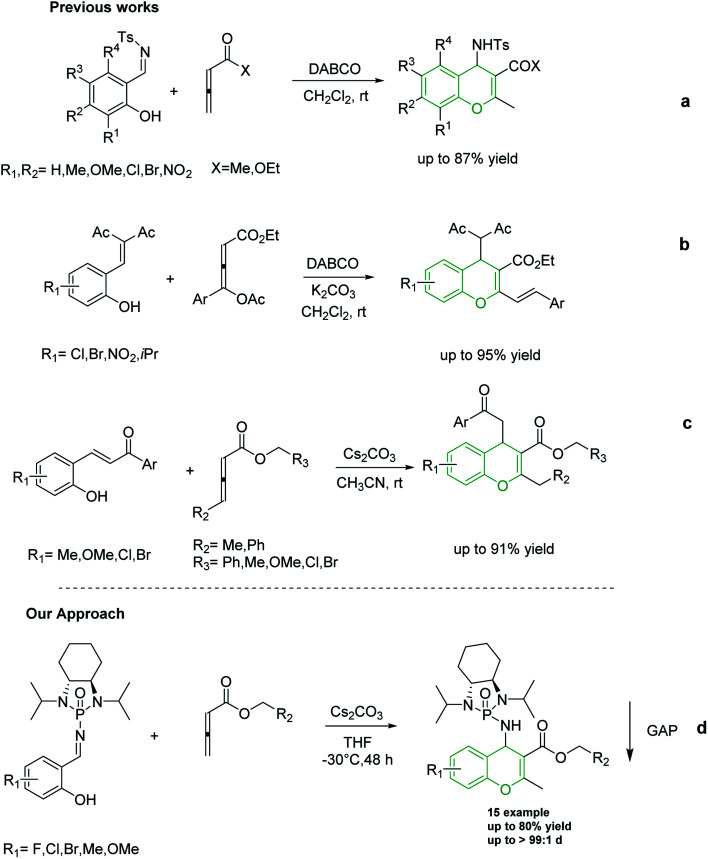
Previously reported methods for the synthesis of 4*H*-chromenes from allene molecules.

Shi and co-workers utilized allene esters and ketones to establish the first synthesis of chromenes *via* [4 + 2] cycloaddition assisted by nitrogen-based catalysts such as DABCO and DBU (Scheme 2a).^37–39^ In 2015, Tong and co-workers reported the synthesis of 4*H*-chromenes from δ-acetoxy allenoates with salicylaldehyde derivatives in an amine-catalyzed reaction (Scheme 2b).^25^ Although in both of the aforementioned methods 4*H*-chromenes were prepared in excellent yields, the enantioselectivity of these methods still need to be improved. In recent years, carbonate catalyzed reactions of allenes with nucleophiles have been recognized as an appropriate method for the synthesis of chromenes. In 2018, our group synthesized 4*H*-chromenes from the reaction of hydroxychalcones and allenoates in the presence of cesium carbonate in high yield and chemo-selectivity (Scheme 2c).^34^

Over the past decade, our group has developed Group-Assisted Purification (GAP) chemistry.^40–44^ In essence, highly functionalized chiral *N*-phosphonyl/*N*-phosphinyl imines were utilized as auxiliaries in asymmetric reactions to synthesize valuable frameworks. GAP technology represents a practical method to prepare target compounds in high yield and diastereoselectivity. In addition, it provides a benign and facile method to separate the final product after completion of the reaction by simply washing the crude mixture with common solvents such as hexanes to bypass expensive and time-consuming traditional methods such as column chromatography and recrystallization.^40^ Many asymmetric reactions were successfully performed *via* GAP chemistry, including a Mannich-type reaction,^45^ aza-MBH reaction,^46^ Strecker reaction,^43^ Umpolung reaction,^47^ and the synthesis of peptides^48^ among others. Recently, our group discovered a novel form of chirality, multi-layer 3D chirality, *via* GAP chemistry in which restriction of the free rotation of intramolecular layers generates chirality.^49–52^

To the best of our knowledge, although several routes have been reported for the synthesis of 4*H*-chromenes through applying auxiliary group, a practical method for the diastereoselective synthesis of these valuable synthons has yet to be devised. The broad spectrum of pharmaceutical properties of this class of heterocyclic compounds and their abundance in natural products with low toxicity inspired us to develop a novel method of preparing them with high chemo and diastereoselectivity. Herein, continuing our investigations into the construction of heterocyclic compounds enabled by GAP technology, we report a facile and efficient [4 + 2] cycloaddition reaction to synthesize 4*H*-chromenes. Salicyl *N*-phosphonyl imines react with allenoates in the presence of cesium carbonate and THF at −30 degrees to yield 4*H*-chromenes in good yield (up to 72%) and high chemo and diastereoselectivity (up to 99%) (Scheme 2d).

## Results and discussion

At the outset of our investigation, salicyl *N*- phosphonyl imine 1a was subjected to benzyl buta-2,3-dienoate 2a (2.00 equiv.) in the presence of various combinations of solvents and bases (2.00 equiv.) at room temperature. The progress of the reactions was monitored by thin layer chromatography and ^31^P NMR. The results are summarized in Table 1. In the presence of lithium hydroxide monohydrate in dry THF, 4*H*-chromene was obtained in 45% yield and 80 : 20 diastereoselectivity (Table 1, entry 1). Then, other solvent systems in the presence of LiOH·H_2_O were examined. Dimethylsulfoxide (DMSO), toluene and acetonitrile resulted in lower yield and dr while no product was isolated in dichloromethane and chloroform (Table 1, entries 2–6). As result, we recognized THF as the optimal solvent. Next, we tried other inorganic and organic bases. The combination of potassium carbonate and dry THF furnished 58% of the desired product with good diastereoselectivity (Table 1, entry 7). Although anhydrous potassium phosphate resulted in better yield (62%), it decreased the dr (Table 1, entry 8). To our surprise, the reactions with organic bases, DABCO (1,4-diazabicyclo[2.2.2]octane) and DBU (1,8-diazabicyclo[5.4.0]undec-7-ene), failed to yield the desired product (Table 1, entries 9 and 10). To our delight, the reaction of salicyl *N*-phosphonyl imine and benzyl buta-2,3-dienoate in dry THF in the presence of cesium carbonate yielded 66% of desired product with good diastereoselectivity (82 : 18) (Table 1, entry 11). Considering cesium carbonate and dry THF as the ideal combination for reaction, we attempted to optimize the amount of allenoate reagent and base. Increasing the amount of both allenoate and base from 2.0 to 3.0 equiv. improved the yield of the reaction while not affecting the dr significantly (Table 1, entries 12 and 13). Further increasing the amount of base resulted in undesired side reactions and lower yield (Table 1, entry 14). Increasing the amount of the allenoate reagent appeared to hinder the reaction (Table 1, entry 15). Finally, the effect of temperature was investigated. Although reducing the reaction temperature to 0 °C decreased the reaction yield, it improved the dr to 86 : 14 (Table 1, entry 16). The diastereoselectivity did not improve at −20 °C while the rate of reaction decreased (Table 1, entry 17). Although lowering the reaction temperature to −30 °C decreased the reaction rate, it improved the dr to 99 : 1 (Table 1, entry 18). However, the reaction did not proceed to completion at −40 °C and most of the salicyl *N*-phosphonyl imine was not consumed (Table 1, entry 19).

**Table 1:** Screening the reaction conditions

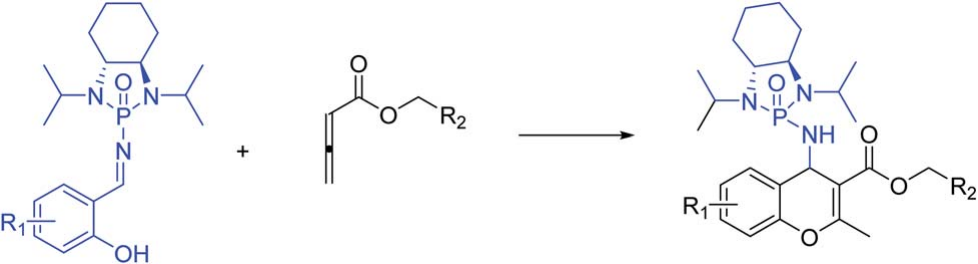						
Entry^a^	Base	Solvent	Temp.	Time (h)	Yield^b^ (%)	dr^c^
1	LiOH·H_2_O	THF	Rt	36	45%^e^^,^^h^	80 : 20
2	LiOH·H_2_O	DMSO	Rt	36	40%^h^	50 : 50
3	LiOH·H_2_O	Toluene	Rt	36	32%^h^	77 : 23
4	LiOH·H_2_O	CH_3_CN	Rt	36	30%^h^	75 : 25
5	LiOH·H_2_O	CH_2_Cl_2_	Rt	36	N.R	—
6	LiOH·H_2_O	CH_3_Cl	Rt	36	N.R	—
7	K_2_CO_3_	THF	Rt	36	58%	81 : 19
8	K_3_PO_4_	THF	Rt	36	62%	78 : 22
9	DABCO	THF	Rt	36	N.R	—
10	DBU	THF	Rt	36	N.R	—
11	Cs_2_CO_3_	THF	Rt	36	66%	82 : 18
12	Cs_2_CO_3_^d^	THF	Rt	36	68%	80 : 20
13^e^	Cs_2_CO_3_^d^	THF	Rt	36	72%	82 : 18
14	Cs_2_CO_3_^f^	THF	Rt	36	41%^h^	78 : 22
15^g^	Cs_2_CO_3_^d^	THF	Rt	36	65%	81 : 19
16	Cs_2_CO_3_^d^	THF	0	36	59%	86 : 14
17	Cs_2_CO_3_^d^	THF	−20	48	60%	86 : 14
18	Cs_2_CO_3_^d^	THF	−30	36	68%	99 : 1
19	Cs_2_CO_3_^d^	THF	−30	36	Trace	—

a Reactions were performed with salicyl *N*-phosphonyl imine (1 mmol), base (2 mmol) and benzyl buta-2,3-dienoate (2 mmol) in dry solvents for 24 hours.

b Isolated yields after GAP washing.

c The dr is determined from ^31^P NMR of the crude mixture.

d 3 equiv. of base was loaded.

e 3 equiv. of allenoate was loaded.

f 4 equiv. of base was loaded.

g 4 equiv. of allenoate was loaded.

h Products were separated by column chromatography EA : hexanes (4 : 6).

**Scheme 3:**
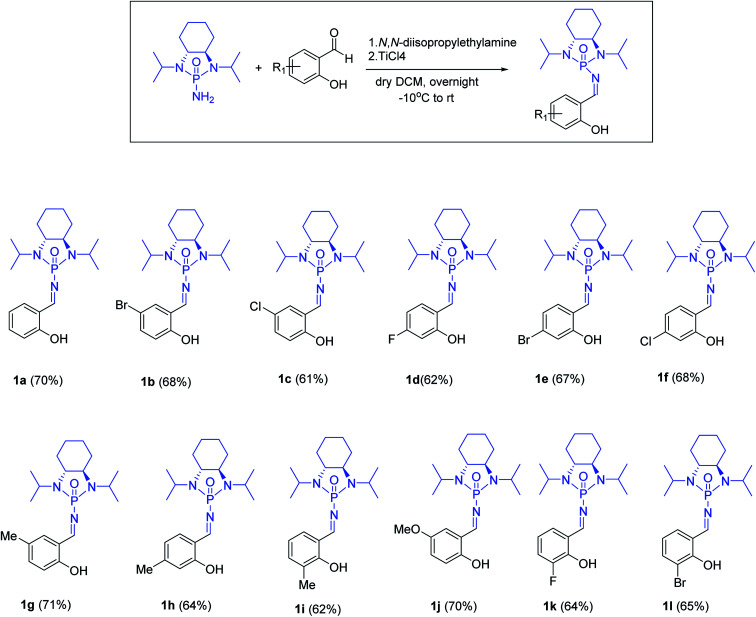
Substrate scope for the synthesis of salicyl *N*-phosphonylimines.

With optimized reaction conditions in hand, a wide range of annulation reactions implementing various substitution patterns on the salicyl *N*-phosphonyl imine and allenoate were evaluated with regard to the synthesis of 4*H*-chromenes. A wide range of enantiomerically pure salicyl *N*-phosphonyl auxiliary imines were isolated in the presence of TiCl_4_ and *N*,*N*-diisopropylethylamine in dry dichloromethane and under argon gas. Enantiopure salicyl *N*-phosphonyl imines with both electron-withdrawing electron-donating groups on various positions of the aromatic ring were synthesized in good yields and excellent diastereoselectivity (Scheme 2). In addition, various allenoates were prepared according to a previously reported method (Scheme 3).^53^

**Scheme 4:**
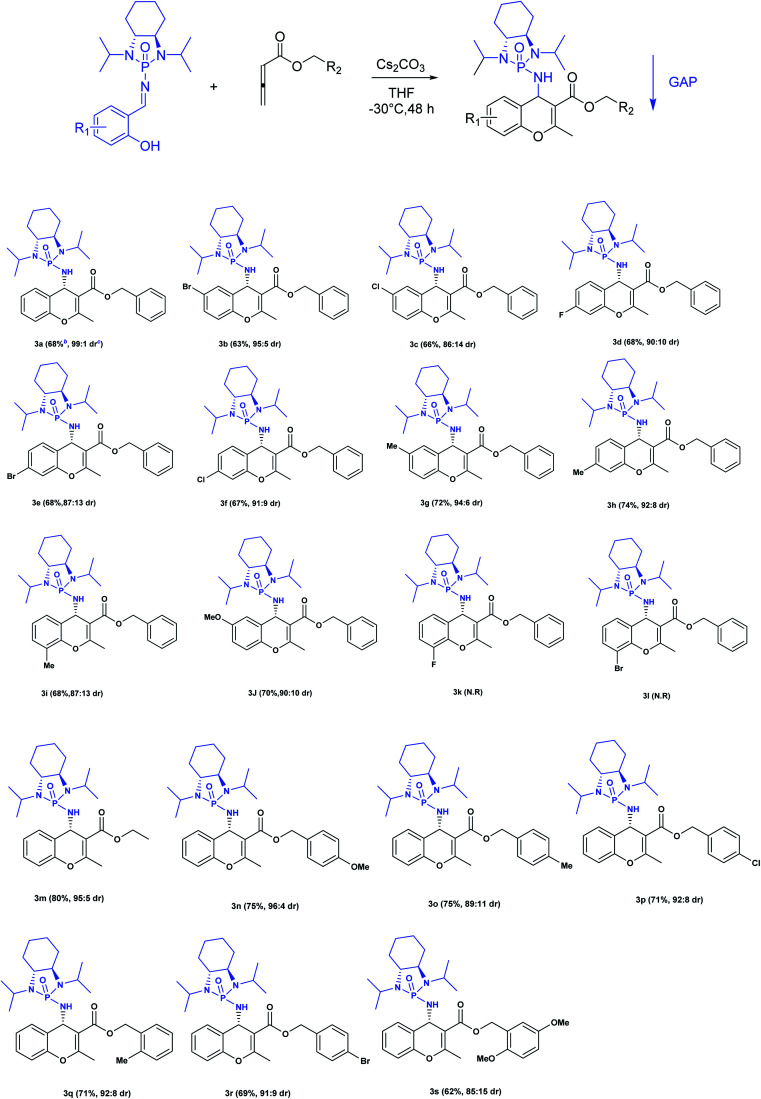
Substrate scope of [4 + 2] annulation for the synthesis of functionalized chromenes.^*a a*^ Reactions were performed with salicyl *N*-phosphonyl imine (1 mmol), cesium carbonate (3 mmol) and allenoate (3 mmol) in dry THF at −35 °C for 48 h. ^*b*^ Isolated yields after GAP washing. ^*c*^ The dr is determined from ^31^P NMR of the crude mixture.

**Fig. 2:**
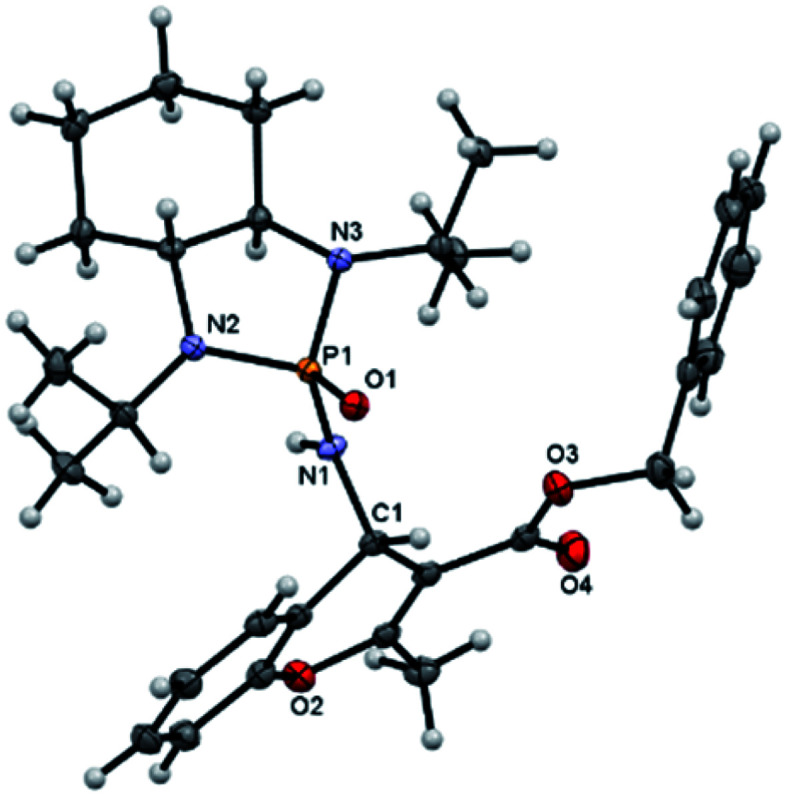
X-ray structure of product 3a.

Next, various salicyl *N*-phosphonyl imines were subjected to allenoates (3.0 equiv.) in the presence of cesium carbonate (3.0 equiv.) and dry THF at −30 °C. A broad scope of functionalized 4*H*-chromenes were synthesized in good yield (up to 78%) and excellent diastereoselectivity (up to 99 : 1). A variety of substituents on the phenolic moiety of the salicyl *N*-phosphonyl group, including MeO, Me, Cl, F and Br, were successfully implemented in these transformations under the above conditions. With an electron-donating group on the aromatic ring of the salicyl *N*-phosphonyl imine, the 4*H*-chromene was obtained smoothly in high yield and excellent diastereoselectivity. However, when the salicyl *N*-phosphonyl imine possessed an electron-withdrawing group at the *meta* or *para* position on the aromatic ring, the result was that less desired product was observed as it is hypothesized that they reduced the nucleophilicity of the oxygen atom, present on the imine. The presence of an electron deficient group at the *ortho* position reduced the rate of reaction significantly and just a trace amount of desired product was observed after 48 hours. Generally, the substrates with electron-donating groups resulted in slightly better diastereoselectivity compared to electron withdrawing groups. One explanation for lower diastereoselectivity of substrates with halogens as electron withdrawing groups could be the steric hindrance effect of halogen groups. Next, we decided to modify the ester group of the allenoate to expand the synthetic scope. The reaction proceeded smoothly with ethyl 2,3-butadienoate and 80% of product with 95 : 5 diastereoselectivity was isolated. Subsequently, other nonterminal allenoates including 4-OMeC_6_H_4_, 4-MeC_6_H_4,_ 4-ClC_6_H_4,_ 4-BrC_6_H_4_ and 2-MeC_6_H_4_ were subjected to *N*-salicyl phosphonyl imine. In all cases, 4*H*-chromenes were obtained in high yield and dr, demonstrating the efficiency of the reaction. It was observed that the substituent on the allenoate did not affect the reaction yield and diastereoselectivity noticeably (Scheme 4). The structures of the products were unambiguously determined by X-ray crystallographic analysis of compound 3a (Fig. 2).

**Scheme 5:**
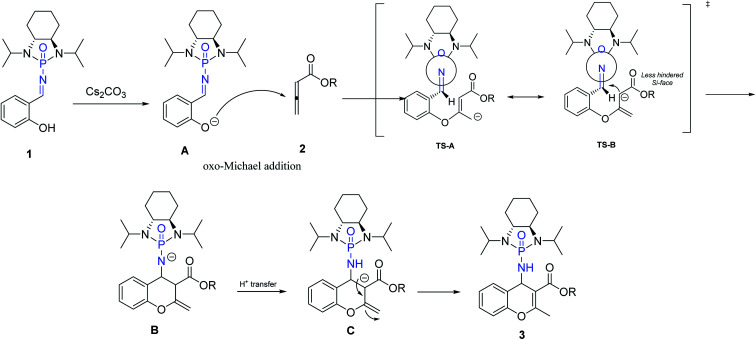
Proposed mechanism for [4 + 2] cycloaddition.

Based on our investigation and previous reports,^12,34,36,54^ especially the recent review regarding stepwise [4 + 2] cycloaddition reactions,^55^ a plausible mechanism for the reaction is represented in Scheme 5. The sequence is initiated by deprotonation of salicyl *N*-phosphonyl imine 1 by cesium carbonate as a non-nucleophilic base to form intermediate A. The oxo-Michael addition of intermediate A to allenoate 2 produces transition state TS-A which is in resonance with TS-B. The following intramolecular cycloaddition of the nucleophilic carbon from the less hindered SI-face of the auxiliary results in intermediate B. Intramolecular proton-transfer forms intermediate C which affords chromene 3 after protonation.

## Conclusions

In summary, a new and facile asymmetric [4 + 2] cycloaddition of salicyl *N*-phosphonyl imines with allenoates has been developed under convenient condition. The reaction provides an easy access to highly functionalized 4*H*-chromenes 4*H*-chromenes in good yield (up to 80%) and excellent diastereoselectivity (up to 99%). The products were conveniently separated from the crude mixtures by simple washing with hexanes to bypass traditional purification methods. This method complements other methods to access functionalized 4*H*-chromene derivatives for potential applications in biological activity screening. Our further studies will be focused on new asymmetric GAP reactions of salicyl *N*-phosphonyl imines for synthesis of a series of new 2,3-dihydrobeznofuran derivatives.

## Conflicts of interest

There are no conflicts to declare.

## Supplementary Material

RA-011-D1RA08323F-s001

RA-011-D1RA08323F-s002
