# Supplementary material for: Asymmetric [4 + 2] cycloaddition synthesis of 4H-chromene derivatives facilitated by group-assisted-purification (GAP) chemistry
Source: RSC Adv. 2021 Dec 14;11(63):39790–6. doi: 10.1039/d1ra08323f (PMC9044656; doi:10.1039/d1ra08323f)
Supplement: RA-011-D1RA08323F-s001 [file RA-011-D1RA08323F-s001.pdf]

Electronic Supplementary Material (ESI) for RSC Advances.
This journal is © The Royal Society of Chemistry 2021

Supporting Information

Asymmetric [4 + 2] Cycloaddition Synthesis of 4H-Chromene Derivatives Facilitated by Group-Assisted-Purification (GAP) Chemistry

Hossein Rouh, Yao Tang, Sai Zhang, Ahmed I.M. Ali, Kazimierz Surowiec, Daniel Unruh and Guigen Li*

[1]Department of Chemistry and Biochemistry, Texas Tech University, Lubbock, Texas 79409-1061, USA

General Aspects……………………………………………………………………………………...S2

General synthesis of salicyl N-phosphonyl imine……………………………………...………...…S2-S6

General synthesis of 4H-chromenes…………………………………………………………..……S7-S15

X-Ray Structure of Products 3a S16-S17

Copies of ^{1}H, ^{31}P, ^{13}C NMR Spectra for Compounds **1a-1l**...S18-S41

Copies of ^{1}H, ^{31}P, ^{13}C NMR Spectra for Compounds **3a-3q**...S42-S75

General Aspects. All commercially available chemicals were used as received without further purification. Solvents were obtained as follows: ether, dichloromethane, tetrahydrofuran, and toluene were delivered from an Innovation Technology solvent system. All reactions were carried out in a flame-dried flask under nitrogen gas. The ^{1}H and ^{13}C NMR spectra were recorded in $CDCl_3$ and CD_3CN on a 400 MHz instrument with TMS as the internal standard. Chemical shifts (δ) were reported in ppm with respect to TMS. Data are represented as follows: chemical shift, mutiplicity (s = singlet, d = doublet, t = triplet, m = multiplet), coupling constant (J, Hz), and integration. ^{31}P NMR spectra were referenced to external H_3PO_4 (0.00 ppm). Shifts in ^{19}F NMR spectra were reported based on an external hexafluorobenzene reference. HRMS analyses were carried out using a TOF-MS instrument with an ESI source. The optical rotations were measured by Rudolph automatic polarimeter model APIV/2W and at room temperature.

EXPERIMENTAL SECTION

General synthesis of salicyl *N*-phosphonyl imine (1a-1n): Into an oven-dried round bottom flask, flushed and protected by argon, phosphoramide (1g, 3.85 mmol) and salicylaldehyde (5.78 mmol) were dissolved in 40 ml dry dichloromethane. After 5 minutes, the mixture was cooled to -10° C, followed by drop-wise addition of diisopropylethylamine (11.56 mmol) and $TiCl_4$ in DCM (1M, 3.08 mmol). The reaction stirred at -10°C for 30 minutes and room temperature overnight. Next, the mixture was concentrated to 5 ml by rotary vapor. The remained solution was passed from a pad of silica gel, eluted with Hexanes: Ethyl acetate (v/v 7:3 to 3:7) to provide salicyl *N*-phosphonyl imine as a yellow solid.

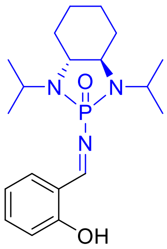

***(3aR,7aR)-2-((2-hydroxybenzylidene)amino)-1,3-diisopropyloctahydrobenzo[d][1,3,2]diazaphosphole 2-oxide* (1a).** Yellow solid, 0.981 g, 70% yield; mp 180 ^{o}C; dr 99.1, $[\alpha]_D^{25}$ +10.6 (c 0.45, $CHCl_3$); ^{1}H NMR (400 MHz, $CDCl_3$) δ 9.09 (d, J = 29.0 Hz, 1H), 7.45 – 7.36 (m, 2H), 7.00 – 6.88 (m, 2H), 3.46 – 3.26 (m, 2H), 3.11 – 3.02 (m, 1H), 2.90 (td, J = 10.4, 3.1 Hz, 1H), 2.14 – 1.92 (m, 2H), 1.80 (d, J = 6.4 Hz, 2H), 1.40 – 1.27 (m, 3H), 1.27 – 1.14 (m, 10H), 1.09 (d, J = 6.7 Hz, 3H); ^{13}C NMR (101 MHz, $CDCl_3$) δ 175.50 (s), 175.44 (s), 162.84 (d, J = 1.9 Hz), 135.04 (s), 133.94 (s), 119.28 (s), 117.62 (s), 60.01 (d, J = 2.5 Hz), 59.92 (d, J = 2.4 Hz), 45.09 (d, J = 3.3 Hz), 44.49 (d, J = 3.6 Hz), 29.85 (d, J = 9.7 Hz), 29.57 (d, J = 9.5 Hz), 24.41 – 24.31 (m), 21.98 (d, J = 3.7 Hz), 21.21 (s), 21.19 (s), 20.85 (s), 20.38 (d, J = 1.5 Hz); ^{31}P NMR (162 MHz, $CDCl_3$) δ 21.81. ; HRMS (TOF ES+) m/z calcd for $C_{19}H_{30}N_3O_2P$ [$(M + H)^+$], 364.2075; found, 364.2163

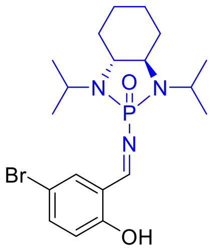

***(3aR,7aR)-2-((5-bromo-2-hydroxybenzylidene)amino)-diisopropyloctahydrobenzo[d][1,3,2]diazaphosphole 2-oxide* (1b).**Yellow solid, 1.16 g, 68% yield; mp 113 ^{o}C; dr 99.1 $[\alpha]_D^{25}$ +16 (c 0.45, $CHCl_3$); ^{1}H NMR (400 MHz, $CDCl_3$) δ 9.01 (d, J = 28.4 Hz, 1H), 7.52 (d, J = 2.5 Hz, 1H), 7.47 (dd, J = 8.8, 2.4 Hz, 1H), 6.88 (d, J = 8.9 Hz, 1H), 3.46 – 3.28 (m, 2H), 3.16 – 3.01 (m, 1H), 2.96 –

2.79 (m, 1H), 2.11 – 2.03 (m, 2H), 1.82 (d, J = 7.2 Hz, 2H), 1.44 – 1.30 (m, 3H), 1.27 – 1.16 (m, 10H), 1.09 (d, J = 6.7 Hz, 3H); ^{13}C NMR (101 MHz, $CDCl_3$) δ 173.70 (d, J = 6.1 Hz), 161.93 (d, J = 1.9 Hz), 137.60 (s), 135.68 (s), 120.46 (d, J = 20.0 Hz), 119.76 (s), 110.68 (d, J = 1.3 Hz), 60.03 (d, J = 6.9 Hz), 59.94 (d, J = 7.2 Hz), 45.18 (d, J = 3.2 Hz), 44.54 (d, J = 3.5 Hz), 29.78 (d, J = 9.7 Hz), 29.55 (d, J = 9.4 Hz), 24.34 (s), 21.97 (s), 21.94 (s), 21.19 (d, J = 2.8 Hz), 20.85 (d, J = 1.3 Hz), 20.45 (d, J = 1.5 Hz); ^{31}P NMR (162 MHz, $CDCl_3$) δ 21.44. ; HRMS (TOF ES+) m/z calcd for $C_{19}H_{29}BrN_3O_2P$ [$(M + H)^+$], 443.1180; found, 442.1271

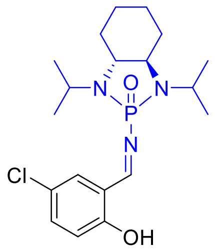

***(3aR,7aR)-2-((5-chloro-2-hydroxybenzylidene)amino)-1,3-diisopropyloctahydrobenzo[d][1,3,2]diazaphosphole 2-oxide* (1c).** Yellow solid, 0.935 g, 61% yield; mp 104 ^{o}C; dr 99.1 $[\alpha]_D^{25}$ -25.6 (c 2.2, $CHCl_3$); ^{1}H NMR (400 MHz, $CDCl_3$) δ 8.99 (d, J = 28.5 Hz, 1H), 7.36 (d, J = 2.5 Hz, 1H), 7.31 (dd, J = 8.8, 2.5 Hz, 1H), 6.89 (d, J = 8.8 Hz, 1H), 3.50 – 3.22 (m, 2H), 3.17 – 2.95 (m, 1H), 2.84 (dd, J = 13.3, 6.1 Hz, 1H), 2.08 – 2.00 (m, 2H), 1.80 (d, J = 6.3 Hz, 2H), 1.33 (t, J = 9.5 Hz, 3H), 1.20 (t, J = 6.4 Hz, 10H), 1.07 (d, J = 6.7 Hz, 3H); ^{13}C NMR (101 MHz, $CDCl_3$) δ 173.79 (d, J = 6.2 Hz), 161.42 (d, J = 1.9 Hz), 134.80 (s), 132.59 (s), 123.86 (d, J = 1.2 Hz), 119.80 (d, J = 20.3 Hz), 119.32 (d, J = 0.8 Hz), 60.01 (d, J = 6.4 Hz), 59.91 (d, J = 6.8 Hz), 45.15 (d, J = 3.2 Hz), 44.52 (d, J = 3.5 Hz), 29.76 (d, J = 9.7 Hz), 29.53 (d, J = 9.4 Hz), 24.32 (s), 21.96 (s), 21.93 (s), 21.17 (d, J = 2.7 Hz), 20.83 (d, J = 1.2 Hz), 20.43 (d, J = 1.4 Hz); ^{31}P NMR (162 MHz, $CDCl_3$) δ 21.51 ; HRMS (TOF ES+) m/z calcd $C_{19}H_{29}ClN_3O_2P$ [$(M + H)^+$], 398.1685; found, 398.1771

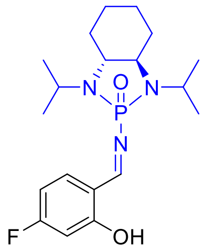

***(3aR,7aR)-2-((4-fluoro-2-hydroxybenzylidene)amino)-1,3-diisopropyloctahydrobenzo[d][1,3,2]diazaphosphole 2-oxide* (1d).** Yellow solid, 0.912 g, 62% yield; mp 186 ^{o}C; dr 99.1 $[\alpha]_D^{25}$ 23.7 (c 0.35 $CHCl_3$); ^{1}H NMR (400 MHz, $CDCl_3$) δ 8.99 (d, J = 28.8 Hz, 1H), 7.14 – 7.01 (m, 2H), 6.87 (dd, J = 8.7, 4.3 Hz, 1H), 3.45 – 3.20 (m, 2H), 3.09 – 2.94 (m, 1H), 2.83 (dd, J = 13.1, 6.0 Hz, 1H), 2.02 (s, 2H), 1.76 (d, J = 6.1 Hz, 2H), 1.30 (t, J = 9.3 Hz, 3H), 1.17 (dd, J = 9.4, 4.8 Hz, 10H), 1.05 (d, J = 6.6 Hz, 3H) ; ^{13}C NMR (101 MHz, $CDCl_3$) δ 174.12 (dd, J = 6.2, 2.7 Hz), 158.95 (d, J = 1.6 Hz), 155.44 (dd, J = 238.2, 1.3 Hz), 122.36 (d, J = 23.6 Hz), 118.95 – 118.68 (m), 118.59 (d, J = 7.0 Hz), 118.26 (d, J = 22.7 Hz), 59.98 (d, J = 3.7 Hz), 59.89 (d, J = 3.9 Hz), 45.10 (d, J = 3.3 Hz), 44.47 (d, J = 3.5 Hz), 29.77 (d, J = 9.7 Hz), 29.49 (d, J = 9.4 Hz), 24.30 (s), 21.96 (s), 21.93 (s), 21.14 (d, J = 2.7 Hz), 20.81 (d, J = 1.1 Hz), 20.37 (d, J = 1.4 Hz); ^{31}P NMR (162 MHz, $CDCl_3$) δ 21.67 ; HRMS (TOF ES+) m/z calcd $C_{19}H_{29}FN_3O_2P$ [$(M + H)^+$], 382.1981; found, 382.2068

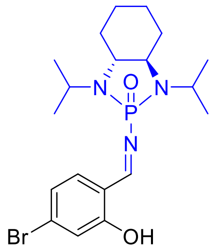

***(3aR,7aR)-2-((4-bromo-2-hydroxybenzylidene)amino)-1,3-diisopropyloctahydrobenzo[d][1,3,2]diazaphosphole 2-oxide* (1e).**Yellow solid, 1.14 g, 67% yield; mp 138 °C; dr 99.1 $[\alpha]_D^{25}$ -12.5(c 0.4, $CHCl_3$); ^{1}H NMR (400 MHz, $CDCl_3$) δ 9.02 (d, J = 28.0 Hz, 1H), 7.24 (d, J = 8.3 Hz, 1H), 7.13 (d, J = 1.7 Hz, 1H), 7.03 (dd, J = 8.3, 1.8 Hz, 1H), 3.45 – 3.27 (m, 2H), 3.11 – 3.01 (m, 1H), 2.94 – 2.83 (m, 1H), 2.04 (d, J = 10.7 Hz, 2H), 1.80 (d, J = 6.2 Hz, 2H), 1.42 – 1.29 (m, 3H), 1.21 (dd, J = 14.3, 6.9 Hz, 11H), 1.08 (d, J = 6.7 Hz, 3H); ^{13}C NMR (101 MHz, $CDCl_3$) δ 174.31 (d, J = 6.0 Hz), 163.92 (s), 134.74 (s), 129.92 (s), 122.70 (s), 121.21 (d, J = 0.8 Hz), 118.00 (d, J = 19.5 Hz), 60.04 (d, J = 2.2 Hz), 59.95 (d, J = 2.0 Hz), 45.12 (d, J = 3.3 Hz), 44.49 (d, J = 3.7 Hz), 29.82 (d, J = 9.8 Hz), 29.51 (d, J = 9.5 Hz), 24.36 – 24.30 (m), 22.01 (s), 21.97 (s), 21.17 (d, J = 2.8 Hz), 20.85 (d, J = 1.4 Hz), 20.39 (d, J = 1.5 Hz); ^{31}P NMR (162 MHz, $CDCl_3$) *δ* 21.29 ; HRMS (TOF ES+) m/z calcd for $C_{19}H_{29}BrN_3O_2P$ [(M + H)$^+$], 442.1180; found, 442.1272

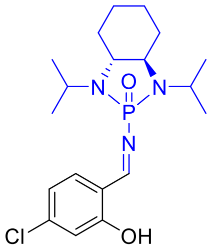

***(3aR,7aR)-2-((4-chloro-2-hydroxybenzylidene)amino)-1,3-diisopropyloctahydrobenzo[d][1,3,2]diazaphosphole 2-oxide* (1f).**Yellow solid, 1.04 g, 68% yield; mp 138 °C; dr 99.1 $[\alpha]_D^{25}$ +8.88 (c 0.45, $CHCl_3$); ^{1}H NMR (400 MHz, $CDCl_3$) δ 9.03 (d, J = 28.0 Hz, 1H), 7.32 (d, J = 8.3 Hz, 1H), 6.96 (d, J = 2.0 Hz, 1H), 6.87 (dd, J = 8.3, 1.9 Hz, 1H), 3.44 – 3.26 (m, 2H), 3.11 – 3.01 (m, 1H), 2.95 – 2.83 (m, 1H), 2.12 – 1.98 (m, 2H), 1.80 (d, J = 6.4 Hz, 2H), 1.42 – 1.25 (m, 3H), 1.26 – 1.12 (m, 11H), 1.09 (d, J = 6.7 Hz, 3H); ^{13}C NMR (101 MHz, $CDCl_3$) δ 174.12 (d, J = 5.9 Hz), 164.11 (d, J = 1.9 Hz), 141.26 (s), 134.71 (s), 119.85 (s), 118.10 (d, J = 0.7 Hz), 117.71 (d, J = 19.6 Hz), 60.04 (d, J = 2.2 Hz), 59.95 (d, J = 2.0 Hz), 45.12 (d, J = 3.3 Hz), 44.49 (d, J = 3.6 Hz), 29.83 (d, J = 9.7 Hz), 29.52 (d, J = 9.5 Hz), 25.34 – 23.53 (m), 22.01 (s), 21.97 (s), 21.17 (d, J = 2.8 Hz), 20.86 (d, J = 1.4 Hz), 20.39 (d, J = 1.5 Hz); ^{31}P NMR (162 MHz, $CDCl_3$) *δ* 21.33 ; HRMS (TOF ES+) m/z calcd for $C_{19}H_{29}ClN_3O_2P$ [(M + H)$^+$], 398.1685; found, 398.1773

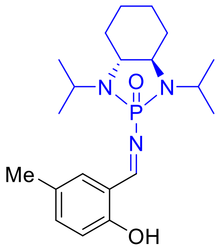

***(3aR,7aR)-2-((2-hydroxy-5-methylbenzylidene)amino)-1,3-diisopropyloctahydrobenzo[d][1,3,2]diazaphosphole 2-oxide* (1g).**Yellow solid, 1.03 g, 71% yield; mp 85 °C; dr 99.1 $[\alpha]_D^{25}$ +16.6 (c 0.45, $CHCl_3$); ^{1}H NMR (400 MHz, CDCl3) δ 9.05 (d, J = 29.1 Hz, 1H), 7.24 – 7.17 (m, 2H), 6.87 (d, J = 8.2 Hz, 1H), 3.46 – 3.26 (m, 2H), 3.13 – 3.01 (m, 1H), 2.88 (td, J = 10.0, 3.1 Hz, 1H), 2.28 (s, 3H), 2.15 (s, 1H), 2.04 (dd, J = 11.0, 6.2 Hz, 2H), 1.80 (d, J = 6.7 Hz, 2H), 1.35 (dd, J = 13.6, 5.5 Hz, 3H), 1.26 – 1.15 (m, 10H), 1.09 (d, J = 6.7 Hz, 3H); ^{13}C NMR (101 MHz, $CDCl_3$) δ 175.37 (d, J = 6.4 Hz), 160.69 (d, J = 2.2 Hz), 136.09 (s), 133.68 (s), 128.43 (d, J = 1.1 Hz), 118.89 (d, J = 20.1 Hz), 117.41 (d, J = 1.2 Hz), 60.00 (d, J = 1.6 Hz), 59.91 (d, J = 1.5 Hz), 45.09 (d, J = 3.6 Hz), 44.50 (d, J = 3.8 Hz), 29.83 (d, J = 9.8 Hz), 29.58 (d, J = 9.5 Hz), 24.64 – 23.72 (m), 21.95 (s), 21.91 (s), 21.19 (d, J = 3.0 Hz), 20.85 (d, J = 1.8 Hz), 20.39 (s), 20.37 (s); ^{31}P NMR (162 MHz, $CDCl_3$) *δ* 22.02 ; HRMS (TOF ES+) m/z calcd for $C_{20}H_{32}N_3O_2P$ [(M + H)$^+$], 378.2232; found, 378.2318

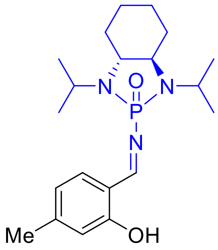

(3aR,7aR)-2-((2-hydroxy-4-methylbenzylidene)amino)-1,3-diisopropyloctahydrobenzo[d][1,3,2]diazaphosphole 2-oxide **(1h).** Yellow solid, 0.932 g, 64% yield; mp 124 ^{o}C; dr 99.1 $[\alpha]_D^{25}$ +17 (c 0.3, $CHCl_3$); ^{1}H NMR (400 MHz, $CDCl_3$) δ 9.01 (d, J = 28.9 Hz, 1H), 7.26 (d, J = 8.3 Hz, 1H), 6.73 (s, 1H), 6.70 (d, J = 7.9 Hz, 1H), 3.45 – 3.23 (m, 2H), 3.09 – 2.98 (m, 1H), 2.86 (td, J = 10.2, 3.1 Hz, 1H), 2.30 (s, 3H), 2.09 – 1.98 (m, 2H), 1.77 (d, J = 6.0 Hz, 2H), 1.23 – 1.15 (m, 10H), 1.06 (d, J = 6.7 Hz, 3H); ^{13}C NMR (101 MHz, $CDCl_3$) δ 174.90 (d, J = 6.2 Hz), 163.04 (d, J = 1.9 Hz), 146.67 (s), 133.76 (s), 120.56 (s), 117.86 (d, J = 0.8 Hz), 117.01 (d, J = 20.0 Hz), 59.97 (d, J = 4.6 Hz), 59.88 (d, J = 4.5 Hz), 45.04 (d, J = 3.3 Hz), 44.44 (d, J = 3.6 Hz), 29.83 (d, J = 9.8 Hz), 29.54 (d, J = 9.4 Hz), 24.34 (dd, J = 2.0, 1.3 Hz), 22.13 (s), 21.93 (s), 21.90 (s), 21.15 (d, J = 2.8 Hz), 20.83 (d, J = 1.4 Hz), 20.32 (d, J = 1.5 Hz); ^{31}P NMR (162 MHz, $CDCl_3$) 22.01 ; HRMS (TOF ES+) m/z calcd for $C_{20}H_{32}N_3O_2P$ [(M + H)$^+$], 378.2232; found, 378.2317

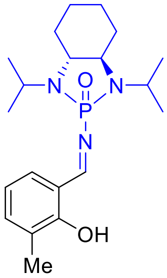

(3aR,7aR)-2-((2-hydroxy-3-methylbenzylidene)amino)-1,3-diisopropyloctahydrobenzo[d][1,3,2]diazaphosphole 2-oxide **(1i).** Yellow solid, 0.902 g, 62% yield; mp 90 ^{o}C; dr 99.1 $[\alpha]_D^{25}$ +2.9 (c 0.55 $CHCl_3$); ^{1}H NMR (400 MHz, $CDCl_3$) δ 9.09 (d, J = 28.9 Hz, 1H), 7.26 (d, J = 7.5 Hz, 2H), 6.82 (t, J = 7.5 Hz, 1H), 3.47 – 3.24 (m, 2H), 3.12 – 3.00 (m, 1H), 2.92 (dd, J = 12.9, 6.1 Hz, 1H), 2.25 (s, 3H), 2.04 (d, J = 10.6 Hz, 2H), 1.80 (d, J = 7.1 Hz, 2H), 1.33 (q, J = 10.2 Hz, 3H), 1.25 – 1.16 (m, 10H), 1.10 (d, J = 6.6 Hz, 3H); ^{13}C NMR (101 MHz, $CDCl_3$) δ 175.86 (d, J = 6.2 Hz), 161.35 (d, J = 1.9 Hz), 135.94 (s), 131.61 (s), 126.61 (d, J = 0.7 Hz), 118.76 (s), 118.50 (d, J = 19.7 Hz), 60.07 (s), 59.99 (d, J = 2.2 Hz), 59.91 (s), 45.07 (d, J = 3.4 Hz), 44.48 (d, J = 3.7 Hz), 29.88 (d, J = 9.8 Hz), 29.55 (d, J = 9.4 Hz), 24.37 (t, J = 1.3 Hz), 22.06 (s), 22.02 (s), 21.18 (d, J = 2.6 Hz), 20.87 (d, J = 1.5 Hz), 20.41 (d, J = 1.5 Hz).; ^{31}P NMR (162 MHz, $CDCl_3$) *δ* 21.73 ; HRMS (TOF ES+) m/z calcd for $C_{20}H_{32}N_3O_2P$ [(M + H)$^+$], 378.2232; found, 378.2318

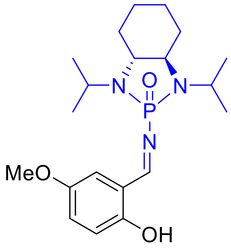

(3aR,7aR)-2-((2-hydroxy-5-methoxybenzylidene)amino)-1,3-diisopropyloctahydrobenzo[d][1,3,2]diazaphosphole 2-oxide **(1j).** Yellow solid, 1.06 g, 70% yield; mp 88 ^{o}C; dr 99.1 $[\alpha]_D^{25}$ +18 (c 0.3, $CHCl_3$); ^{1}H NMR (400 MHz, $CDCl_3$) δ 9.06 (d, J = 29.0 Hz, 1H), 7.03 (dd, J = 9.0, 3.1 Hz, 1H), 6.91 (d, J = 9.0 Hz, 2H), 3.77 (s, 3H), 3.48 – 3.22 (m, 2H), 3.13 – 3.01 (m, 1H), 2.91 (t, J = 8.0 Hz, 1H), 2.05 (d, J = 8.8 Hz, 2H), 1.81 (d, J = 5.6 Hz, 2H), 1.41 – 1.29 (m, 3H), 1.28 – 1.16 (m, 10H), 1.10 (d, J = 6.7 Hz, 3H); ^{13}C NMR (101 MHz, $CDCl_3$) δ 175.15 (d, J = 6.2 Hz), 157.37 (s), 152.34 (d, J = 1.0 Hz), 123.26 (s), 118.62 (s), 115.74 (s), 100.00 (s), 60.03 (d, J = 1.6 Hz), 59.94 (d, J = 1.6 Hz), 55.96 (s), 45.10 (d, J = 3.3 Hz), 44.51 (d, J = 3.6 Hz), 29.85 (d, J = 9.8 Hz), 29.55 (d, J = 9.4 Hz), 24.36 (dd, J = 2.2, 1.3 Hz), 22.00 (s), 21.96 (s), 21.19

(d, J = 2.7 Hz), 20.88 (d, J = 1.3 Hz), 20.40 (d, J = 1.3 Hz); ^{31}P NMR (162 MHz, $CDCl_3$) δ 21.98 ; HRMS (TOF ES+) m/z calcd $C_{20}H_{32}N_3O_3P$ [(M + H)$^+$], 394.2181; found, 392.2268

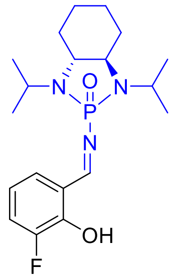

(3aR,7aR)-2-((3-fluoro-2-hydroxybenzylidene)amino)-1,3diisopropyloctahydrobenzo[d][1,3,2]diazaphosphole 2-oxide **(1k).** Yellow solid, 0.941 g, 64% yield; mp 193 °C; dr 99.1 $[\alpha]_D^{25}$ -10.7 (c 0.40, $CHCl_3$); ^{1}H NMR (400 MHz, $CDCl_3$) δ 9.07 (dd, J = 27.3, 1.2 Hz, 1H), 7.24 – 7.16 (m, 2H), 6.90 – 6.75 (m, 1H), 3.48 – 3.27 (m, 2H), 3.12 – 3.01 (m, 1H), 2.98 – 2.87 (m, 1H), 2.06 (t, J = 10.6 Hz, 2H), 1.81 (d, J = 7.1 Hz, 2H), 1.34 (td, J = 11.8, 6.3 Hz, 4H), 1.26 – 1.16 (m, 10H), 1.09 (dd, J = 14.7, 6.7 Hz, 3H); ^{13}C NMR (101 MHz, $CDCl_3$) δ 174.46 (dd, J = 5.8, 2.8 Hz), 152.75 (dd, J = 13.0, 1.9 Hz), 150.40 (d, J = 0.8 Hz), 128.72 (d, J = 3.6 Hz), 120.79 (d, J = 17.5 Hz), 120.57 (d, J = 4.2 Hz), 118.15 (d, J = 6.3 Hz), 60.10 (d, J = 6.2 Hz), 60.01 (d, J = 6.1 Hz), 45.12 (d, J = 3.4 Hz), 44.55 (d, J = 3.7 Hz), 29.82 (d, J = 9.7 Hz), 29.55 (d, J = 9.4 Hz), 24.34 (d, J = 1.1 Hz), 22.06 (s), 22.03 (s), 21.23 (d, J = 2.7 Hz), 20.85 (d, J = 1.5 Hz), 20.45 (d, J = 1.5 Hz); ^{31}P NMR (162 MHz, $CDCl_3$) δ 21.75 ; HRMS (TOF ES+) m/z calcd for $C_{19}H_{29}FN_3O_2P$ [(M + H)$^+$], 382.1981; found, 382.2067

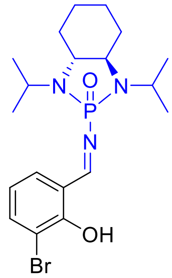

(3aR,7aR)-2-((3-bromo-2-hydroxybenzylidene)amino)-1,3-diisopropyloctahydrobenzo[d][1,3,2]diazaphosphole 2-oxide **(1l).**Yellow solid, 1.10 g, 65% yield; mp 145 °C; dr 99.1 $[\alpha]_D^{25}$ -35.3 (c 0.3 $CHCl_3$); ^{1}H NMR (400 MHz, $CDCl_3$) δ 9.03 (d, J = 26.4 Hz, 1H), 7.68 (d, J = 7.8 Hz, 1H), 7.46 – 7.34 (m, 1H), 6.80 (td, J = 7.8, 1.0 Hz, 1H), 3.36 (ddt, J = 20.3, 13.6, 6.8 Hz, 2H), 3.20 – 2.84 (m, 2H), 2.06 (t, J = 13.2 Hz, 2H), 1.83 (d, J = 7.5 Hz, 2H), 1.46 – 1.27 (m, 3H), 1.21 (dt, J = 13.4, 7.2 Hz, 10H), 1.12 (d, J = 6.7 Hz, 3H); ^{13}C NMR (101 MHz, $CDCl_3$) δ 174.28 (d, J = 5.4 Hz), 161.20 (d, J = 2.2 Hz), 138.30 (s), 133.15 (s), 119.56 (s), 112.23 (d, J = 1.5 Hz), 100.00 (s), 60.12 (d, J = 6.2 Hz), 60.03 (d, J = 6.4 Hz), 45.12 (d, J = 3.6 Hz), 44.56 (d, J = 3.9 Hz), 29.85 (d, J = 9.8 Hz), 29.45 (d, J = 9.5 Hz), 24.35 (s), 22.18 (d, J = 4.0 Hz), 21.21 (s), 21.18 (s), 20.87 (d, J = 2.0 Hz), 20.56 (d, J = 1.8 Hz); ^{31}P NMR (162 MHz, $CDCl_3$) δ 20.21 ; HRMS (TOF ES+) m/z calcd $C_{19}H_{29}BrN_3O_2P$ [(M + H)$^+$], 442.1180; found, 442.1271

General synthesis of 4H-chromenes (3a-3q): Into an oven-dried round bottom flask, flushed and protected by argon, salicyl N-phosphonyl imine (qmol) was dissolved in freshly dried THF (25 ml). After 5 minutes, Cs_2CO_3 (3 mmol) was added, and reaction was stirred at room temperature for 1 hour. The reaction was cooled to -30ºC and after 5 minutes, allenoate (3 mmol) dissolved in 2 ml of dry THF was added dropwise and over 5 minutes. The reaction was stirred at -30ºC for 48 hours and was quenched by addition of 10 ml water. The mixture was extracted with ethyl acetate and combined organic layers were extracted with brine and dried over Na_2SO_4. After that, the solution was concentrated before addition of hexanes and stirring the reaction overnight to precipitate the product. Solid Pure product was obtained by filtration and washing the solid product few times with hexanes.

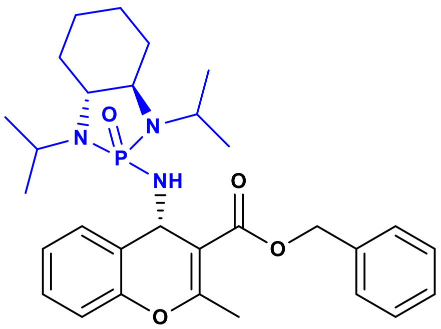

benzyl (S)-4-(((3aR,7aR)-1,3-diisopropyl-2-oxidooctahydrobenzo[d][1,3,2]diazaphosphol-2-yl)amino)-2-methyl-4H-chromene-3-carboxylate (3a). White solid, 0.365 g, 68% yield; mp 143 ºC; $[\alpha]_D^{25}$ +11.5 (c 1.8, CH_3OH),^{1}H NMR (400 MHz, CD_3CN) δ 7.68 (dd, *J* = 7.6, 1.6 Hz, 1H), 7.46 (dd, *J* = 7.9, 1.0 Hz, 2H), 7.36 (ddd, *J* = 7.3, 6.2, 1.4 Hz, 2H), 7.31 (dt, *J* = 9.8, 4.4 Hz, 1H), 7.24 (ddd, *J* = 8.1, 7.4, 1.7 Hz, 1H), 7.13 (td, *J* = 7.5, 1.2 Hz, 1H), 7.03 (dd, *J* = 8.1, 1.2 Hz, 1H), 5.40 (dd, *J* = 9.5, 7.5 Hz, 1H), 5.32 (d, *J* = 12.8 Hz, 1H), 5.08 (d, *J* = 12.8 Hz, 1H), 3.43 – 3.25 (m, 1H), 3.15 (tt, *J* = 13.8, 6.9 Hz, 1H), 2.94 (t, *J* = 10.1 Hz, 1H), 2.75 – 2.65 (m, 1H), 2.63 – 2.49 (m, 1H), 2.39 (s, 3H), 1.91 (dt, *J* = 6.3, 2.5 Hz, 2H), 1.70 – 1.52 (m, 2H), 1.33 – 1.14 (m, 3H), 1.03 (d, *J* = 6.9 Hz, 4H), 0.97 (dd, *J* = 6.9, 5.4 Hz, 6H), 0.65 (d, *J* = 6.9 Hz, 3H).; ^{13}C NMR (101 MHz, CD_3CN) δ 166.99 (s), 162.20 (s), 150.57 (s), 136.55 (s), 129.45 (s), 128.59 (s), 128.06 (s), 125.95 (s), 124.35 (s), 115.74 (s), 106.42 (s), 106.35 (s), 66.03 (s), 58.99 (d, *J* = 10.2 Hz), 58.56 (d, *J* = 10.1 Hz), 45.05 (s), 43.46 (d, *J* = 3.7 Hz), 43.06 (d, *J* = 3.9 Hz), 31.56 (d, *J* = 11.0 Hz), 31.33 (d, *J* = 11.5 Hz), 24.07 (d, *J* = 1.4 Hz), 23.97 (d, *J* = 1.4 Hz), 23.10 (d, *J* = 6.6 Hz), 22.97 (d, *J* = 6.6 Hz), 19.02 (s), 18.65 (s), 17.91 (s).; ^{31}P NMR (162 MHz, CD_3CN) δ 21.85, 21.70 ; HRMS (TOF ES+) m/z calcd $C_{30}H_{40}N_3O_4P$ [(M + H)$^+$], 538.2756; found, 538.2819

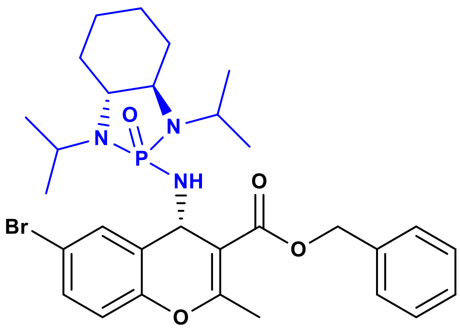

benzyl (S)-6-bromo-4-(((3aR,7aR)-1,3-diisopropyl-2-oxidooctahydrobenzo[d][1,3,2]diazaphosphol-2-yl)amino)-2-methyl-4H-chromene-3-carboxylate (3b). White solid, 0.365 g, 68% yield; mp 141 ºC; $[\alpha]_D^{25}$ -53.2 (c 1.25, CH_3OH),^{1}H NMR (400 MHz, CD_3CN) δ 7.89 (d, *J* = 2.4 Hz, 1H), 7.45 (d, *J* = 7.2 Hz, 3H), 7.37 (ddd, *J* = 7.4, 5.2, 1.9 Hz, 4H), 7.34 – 7.27 (m, 2H), 6.96 (d, *J* = 8.7 Hz, 1H), 5.44 – 5.25 (m, 3H), 5.08 (d, *J* = 12.8 Hz, 1H), 3.31 (tt, *J* = 14.0, 7.0 Hz, 1H), 3.16 (tt, *J* = 13.7, 6.8 Hz, 1H), 3.05 (t, *J* = 10.0 Hz, 1H), 2.79 – 2.65 (m, 1H), 2.58 (dd, *J* = 13.4, 6.3 Hz, 1H), 2.38 (s, 3H), 1.91 (dt, *J* = 4.9, 2.5 Hz, 4H), 1.62 (d, *J*

= 10.5 Hz, 3H), 1.29 – 1.13 (m, 4H), 1.02 (dd, *J* = 6.8, 3.8 Hz, 9H), 0.96 (d, *J* = 6.9 Hz, 4H), 0.70 (d, *J* = 6.9 Hz, 3H).; ^{13}C NMR (101 MHz, CD_3CN) δ 166.74 (s), 161.89 (s), 149.53 (s), 136.45 (s), 132.17 (s), 130.79 (s), 128.61 (s), 128.18 (s), 128.11 (s), 128.06 (s), 117.93 (s), 115.71 (s), 106.03 (s), 66.12 (s), 59.01 (d, *J* = 10.3 Hz), 58.55 (d, *J* = 10.1 Hz), 44.78 (s), 43.52 (d, *J* = 3.7 Hz), 43.25 (d, *J* = 3.9 Hz), 31.61 (d, *J* = 11.0 Hz), 31.25 (d, *J* = 11.5 Hz), 24.05 (d, *J* = 1.4 Hz), 23.96 (d, *J* = 1.4 Hz), 23.00 (d, *J* = 3.5 Hz), 22.94 (d, *J* = 3.3 Hz), 18.93 (s), 18.78 (s), 17.93 (s).; ^{31}P NMR (162 MHz, CD_3CN) δ 21.96, 21.60 ; HRMS (TOF ES+) m/z calcd $C_{30}H_{39}BrN_3O_4P$ [(M + H)$^+$], 616.1861; found, 616.1920

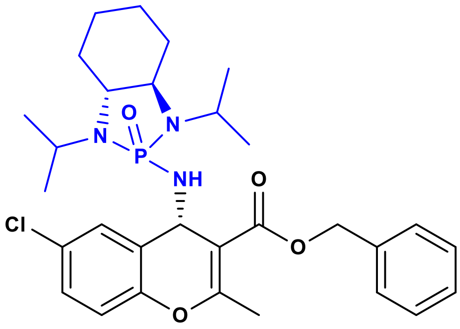

benzyl (S)-6-chloro-4-(((3aR,7aR)-1,3-diisopropyl-2-oxidooctahydrobenzo[d][1,3,2]diazaphosphol-2-yl)amino)-2-methyl-4H-chromene-3-carboxylate (3c). White solid, 0.377 g, 66% yield; mp 155 °C; $[\alpha]_D^{25}$ -28.2 (c 1.7, CH_3OH),^{1}H NMR (400 MHz, CD_3CN) δ 7.74 (d, *J* = 2.6 Hz, 1H), 7.51 – 7.42 (m, 3H), 7.40 – 7.34 (m, 3H), 7.34 – 7.30 (m, 2H), 7.23 (dd, *J* = 8.7, 2.6 Hz, 2H), 5.42 – 5.28 (m, 3H), 5.08 (d, *J* = 12.8 Hz, 2H), 3.40 – 3.22 (m, 2H), 3.14 (tt, *J* = 13.7, 6.9 Hz, 1H), 3.05 (t, *J* = 10.1 Hz, 1H), 2.76 – 2.67 (m, 1H), 2.63 – 2.49 (m, 1H), 2.38 (s, 4H), 1.95 – 1.87 (m, 4H), 1.62 (d, *J* = 10.1 Hz, 3H), 1.27 – 1.11 (m, 5H), 1.07 – 0.88 (m, 14H), 0.70 (d, *J* = 6.9 Hz, 3H).; ^{13}C NMR (101 MHz, CD_3CN) δ 166.73 (s), 161.86 (s), 151.08 (s), 136.43 (s), 132.64 (s), 130.92 (s), 128.60 (s), 128.10 (s), 124.91 (s), 124.37 (s), 115.85 (s), 106.68 (s), 106.60 (s), 66.17 (s), 58.99 (d, *J* = 10.2 Hz), 58.57 (d, *J* = 10.1 Hz), 44.61 (s), 43.49 (d, *J* = 3.6 Hz), 43.14 (d, *J* = 3.9 Hz), 31.57 (d, *J* = 11.0 Hz), 31.29 (d, *J* = 11.5 Hz), 24.06 (d, *J* = 1.4 Hz), 23.96 (d, *J* = 1.4 Hz), 23.04 (d, *J* = 6.6 Hz), 22.95 (d, *J* = 6.5 Hz), 18.85 (s), 18.64 (s), 17.92 (s).; ^{31}P NMR (162 MHz, CD_3CN) δ 21.92, 21.65 ; HRMS (TOF ES+) m/z calcd $C_{30}H_{39}ClN_3O_4P$ [(M + H)$^+$], 572.2366; found, 572.2427

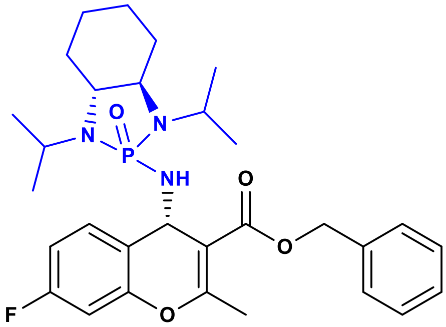

benzyl (S)-4-(((3aR,7aR)-1,3-diisopropyl-2-oxidooctahydrobenzo[d][1,3,2]diazaphosphol-2-yl)amino)-7-fluoro-2-methyl-4H-chromene-3-carboxylate (3d). White solid, 0.367 g, 68% yield; mp 186 °C; $[\alpha]_D^{25}$ +13.7 (c 2.85, CH_3OH),^{1}H NMR (400 MHz, CD_3CN) δ 7.50 – 7.42 (m, 1H), 7.39 – 7.34 (m, 1H), 7.32 (dd, *J* = 5.1, 3.5 Hz, 1H), 7.05 (dd, *J* = 9.0, 4.8 Hz, 1H), 7.02 – 6.94 (m, 1H), 5.45 – 5.25 (m, 1H), 5.07 (d, *J* = 12.7 Hz, 1H), 3.45 – 3.22 (m, 1H), 3.20 – 3.06 (m, 1H), 3.02 (t, *J* = 10.2 Hz, 1H), 2.80 – 2.65 (m, 1H), 2.58 (dd, *J* = 13.5, 6.2 Hz, 1H), 2.38 (s, 1H), 1.91 (dt, *J* = 4.9, 2.5 Hz, 1H), 1.62 (d, *J* = 10.1 Hz, 1H), 1.31 – 1.12 (m, 2H), 1.10 – 0.90 (m, 4H), 0.71 (d, *J* = 6.9 Hz, 1H), 0.71 (d, *J* = 6.9 Hz, 1H).; ^{13}C NMR (101 MHz, CD_3CN) δ ^{13}C NMR (101 MHz, ACETONITRILE-D3) δ 166.79 (s), 162.43 (s), 160.00 (s), 157.61 (s), 146.80 (s), 136.48 (s), 128.62 (s), 127.57 (s), 115.13 (s), 114.89 (s), 114.74 (s), 105.40 (s), 105.32 (s), 66.15 (d, *J* = 7.5 Hz), 66.07 (d, *J* = 7.3 Hz), 59.01 (d, *J* = 10.1 Hz), 58.58 (d, *J* = 9.9 Hz), 45.14 (d, *J* = 5.2 Hz), 43.51 (d, *J* = 3.6 Hz), 43.21 (s), 31.48 (d, *J* = 11.0 Hz), 31.26 (d, *J* = 11.4 Hz), 24.00 (d, *J* = 9.9 Hz), 23.00 (d, *J* = 6.6 Hz),

22.83 (d, *J* = 4.3 Hz), 18.99 (d, *J* = 2.4 Hz), 18.68 (s), 17.94 (s).; ^{31}P NMR (162 MHz, CD_3CN) δ 21.82, 21.73 ; HRMS (TOF ES+) m/z calcd $C_{30}H_{39}FN_3O_4P$ [(M + H)$^+$], 556.2662; found, 556.2723

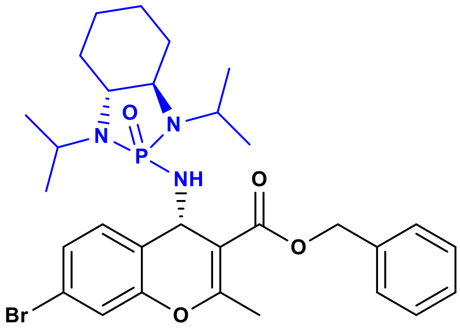

benzyl (S)-7-bromo-4-(((3aR,7aR)-1,3-diisopropyl-2-oxidooctahydrobenzo[d][1,3,2]diazaphosphol-2-yl)amino)-2-methyl-4H-chromene-3-carboxylate (3e). White solid, 0.418 g, 68% yield; mp 148 °C; $[\alpha]_D^{25}$ -4 (c 1.45, $CHCl_3$); ^{1}H NMR (400 MHz, CD_3CN) δ 7.61 (d, *J* = 8.2 Hz, 1H), 7.47 – 7.43 (m, 2H), 7.36 (ddd, *J* = 7.3, 6.1, 1.4 Hz, 2H), 7.33 – 7.27 (m, 2H), 7.24 (d, *J* = 2.0 Hz, 1H), 5.36 (dd, *J* = 9.2, 7.9 Hz, 1H), 5.31 (d, *J* = 12.7 Hz, 1H), 5.08 (d, *J* = 12.7 Hz, 1H), 3.38 – 3.21 (m, 1H), 3.12 (tt, *J* = 13.8, 6.9 Hz, 1H), 3.03 (t, *J* = 10.1 Hz, 1H), 2.75 – 2.63 (m, 1H), 2.56 (td, *J* = 10.3, 3.1 Hz, 1H), 2.37 (s, 3H), 1.95 – 1.86 (m, 3H), 1.61 (d, *J* = 9.7 Hz, 2H), 1.22 (dd, *J* = 14.4, 6.8 Hz, 3H), 1.05 – 0.93 (m, 9H), 0.69 (d, *J* = 6.9 Hz, 3H).; ^{13}C NMR (101 MHz, CD_3CN) δ 166.70 (s), 161.90 (s), 151.21 (s), 136.42 (s), 131.17 (s), 128.61 (s), 128.10 (s), 127.30 (s), 125.29 (s), 120.29 (s), 118.79 (s), 106.64 (s), 106.56 (s), 66.18 (s), 59.00 (d, *J* = 10.4 Hz), 58.58 (d, *J* = 10.1 Hz), 44.66 (s), 43.49 (d, *J* = 3.6 Hz), 43.16 (d, *J* = 3.9 Hz), 31.55 (d, *J* = 11.0 Hz), 31.28 (d, *J* = 11.5 Hz), 24.06 (d, *J* = 1.3 Hz), 23.96 (d, *J* = 1.5 Hz), 23.00 (s), 22.94 (s), 18.89 (s), 18.65 (s), 17.94 (s).; ^{31}P NMR (162 MHz, CD_3CN) δ 21.89, 21.72 ; HRMS (TOF ES+) m/z calcd $C_{30}H_{39}BrN_3O_4P$ [(M + H)$^+$], 616.1861; found, 616.1920

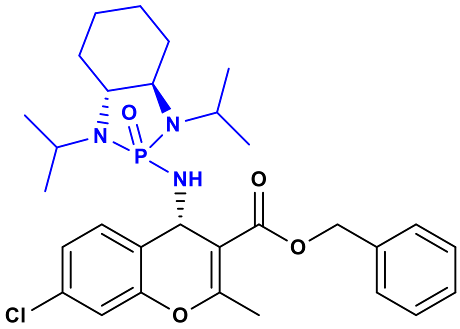

benzyl (S)-7-chloro-4-(((3aR,7aR)-1,3-diisopropyl-2-oxidooctahydrobenzo[d][1,3,2]diazaphosphol-2-yl)amino)-2-methyl-4H-chromene-3-carboxylate (3f). White solid, 0383 g, 67% yield; mp 150 °C; $[\alpha]_D^{25}$ -8.8 (c 0.7, CH_3OH); ^{1}H NMR (400 MHz, CD_3CN) δ 7.68 (d, *J* = 8.3 Hz, 1H), 7.43 (dd, *J* = 14.1, 5.2 Hz, 2H), 7.40 – 7.29 (m, 4H), 7.18 – 7.13 (m, 1H), 7.10 (d, *J* = 2.0 Hz, 1H), 5.41 – 5.34 (m, 1H), 5.32 (d, *J* = 12.7 Hz, 1H), 5.08 (d, *J* = 12.7 Hz, 1H), 3.40 – 3.24 (m, 1H), 3.12 (tt, *J* = 13.7, 6.9 Hz, 1H), 3.02 (t, *J* = 10.1 Hz, 1H), 2.81 – 2.62 (m, 1H), 2.62 – 2.49 (m, 1H), 2.38 (s, 3H), 1.93 (ddd, *J* = 10.2, 7.4, 6.4 Hz, 4H), 1.62 (d, *J* = 10.2 Hz, 2H), 1.21 (ddd, *J* = 9.9, 7.2, 4.1 Hz, 5H), 1.06 – 0.91 (m, 11H), 0.69 (d, *J* = 6.9 Hz, 3H).; ^{13}C NMR (101 MHz, CD_3CN) δ 166.73 (s), 161.86 (s), 151.07 (s), 136.43 (s), 132.64 (s), 130.92 (s), 128.60 (s), 128.10 (s), 124.91 (s), 124.37 (s), 115.85 (s), 106.68 (s), 106.60 (s), 66.17 (s), 58.99 (d, *J* = 10.2 Hz), 58.57 (d, *J* = 10.1 Hz), 44.61 (s), 43.48 (d, *J* = 3.7 Hz), 43.14 (d, *J* = 3.9 Hz), 31.57 (d, *J* = 11.0 Hz), 31.29 (d, *J* = 11.4 Hz), 24.06 (d, *J* = 1.4 Hz), 23.96 (d, *J* = 1.4 Hz), 23.04 (d, *J* = 6.6 Hz), 22.96 (d, *J* = 6.6 Hz), 18.86 (s), 18.65 (s), 17.92 (s).; ^{31}P NMR (162 MHz, CD_3CN) δ 21.87, 21.68 ; HRMS (TOF ES+) m/z calcd $C_{30}H_{39}ClN_3O_4P$ [(M + H)$^+$], 572.2366; found, 572.2429

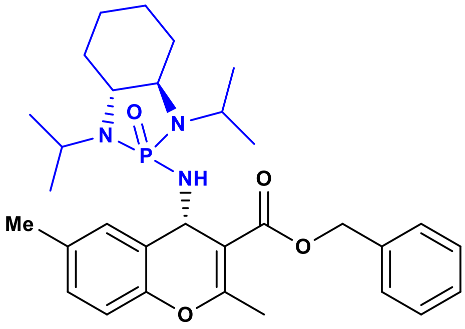

benzyl (S)-4-(((3aR,7aR)-1,3-diisopropyl-2-oxidooctahydrobenzo[d][1,3,2]diazaphosphol-2-yl)amino)-2,6-dimethyl-4H-chromene-3-carboxylate (3g). White solid, 0.396 g, 72% yield; mp 135 °C; $[\alpha]_D^{25}$ +127.2 (c 0.7, CH_3OH); ^1H NMR (400 MHz, CD_3CN) δ 7.54 (d, *J* = 7.8 Hz, 1H), 7.48 – 7.43 (m, 2H), 7.39 – 7.33 (m, 2H), 7.32 (dd, *J* = 5.0, 3.6 Hz, 1H), 6.98 – 6.88 (m, 1H), 6.85 (s, 1H), 5.42 – 5.33 (m, 1H), 5.31 (d, *J* = 12.8 Hz, 1H), 5.07 (d, *J* = 12.8 Hz, 1H), 3.33 (tt, *J* = 13.7, 6.9 Hz, 1H), 3.18 (tt, *J* = 13.7, 6.9 Hz, 1H), 2.90 (t, *J* = 10.0 Hz, 1H), 2.75 – 2.65 (m, 1H), 2.56 (td, *J* = 10.6, 3.4 Hz, 1H), 2.37 (s, 3H), 2.29 (s, 3H), 1.96 – 1.87 (m, 3H), 1.67 – 1.56 (m, 2H), 1.23 (dd, *J* = 11.9, 7.8 Hz, 3H), 1.04 – 0.95 (m, 9H), 0.67 (d, *J* = 6.9 Hz, 3H).; ^{13}C NMR (101 MHz, CD_3CN) δ 167.02 (s), 161.98 (s), 150.40 (s), 138.29 (s), 136.51 (s), 129.13 (s), 128.52 (s), 128.00 (s), 125.13 (s), 122.95 (s), 115.80 (s), 106.45 (s), 106.37 (s), 65.94 (s), 58.91 (d, *J* = 10.4 Hz), 58.50 (d, *J* = 10.1 Hz), 44.80 (s), 43.40 (d, *J* = 3.4 Hz), 43.02 (d, *J* = 3.6 Hz), 31.54 (d, *J* = 11.0 Hz), 31.29 (d, *J* = 11.3 Hz), 23.97 (d, *J* = 9.5 Hz), 23.08 (s), 23.01 (s), 22.93 (s), 20.14 (s), 18.96 (s), 18.60 (s), 17.86 (s).; ^{31}P NMR (162 MHz, CD_3CN) δ 21.95, 21.75 ; HRMS (TOF ES+) m/z calcd $C_{31}H_{42}N_3O_4P$ [(M + H)$^+$], 552.2912; found, 552.2968

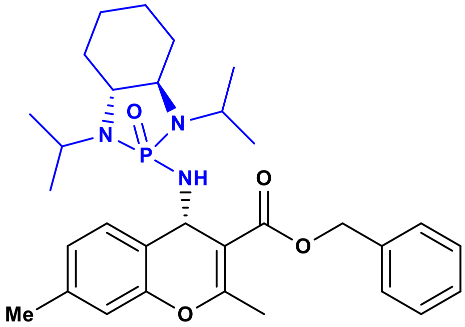

benzyl (S)-4-(((3aR,7aR)-1,3-diisopropyl-2-oxidooctahydrobenzo[d][1,3,2]diazaphosphol-2-yl)amino)-2,7-dimethyl-4H-chromene-3-carboxylate (3h). White solid, 0.407 g, 74% yield; mp 140 °C; $[\alpha]_D^{25}$ -8.0 (c 1.05, CH_3OH); ^1H NMR (400 MHz, CD_3CN) δ 7.54 (d, *J* = 7.8 Hz, 1H), 7.51 – 7.42 (m, 1H), 7.36 (ddd, *J* = 7.3, 4.5, 1.4 Hz, 1H), 7.33 – 7.28 (m, 1H), 6.96 (ddd, *J* = 7.8, 1.6, 0.6 Hz, 1H), 6.87 – 6.83 (m, 1H), 5.36 (dd, *J* = 9.4, 7.6 Hz, 1H), 5.31 (d, *J* = 12.8 Hz, 1H), 5.07 (d, *J* = 12.8 Hz, 1H), 3.41 – 3.26 (m, 1H), 3.18 (tt, *J* = 13.8, 6.9 Hz, 1H), 2.89 (t, *J* = 10.0 Hz, 1H), 2.76 – 2.64 (m, 1H), 2.62 – 2.51 (m, 1H), 2.29 (s, 1H), 2.00 – 1.87 (m, 2H), 1.69 – 1.55 (m, 1H), 1.34 – 1.19 (m, 2H), 1.07 – 0.91 (m, 5H), 0.67 (d, *J* = 6.9 Hz, 1H).; ^{13}C NMR (101 MHz, CD_3CN) δ 167.08 (s), 162.01 (s), 150.46 (s), 138.33 (s), 136.57 (s), 129.20 (s), 128.58 (s), 128.06 (s), 125.18 (s), 123.02 (s), 115.85 (s), 106.51 (s), 106.43 (s), 65.99 (s), 58.96 (d, *J* = 10.3 Hz), 58.55 (d, *J* = 10.1 Hz), 44.85 (s), 43.45 (d, *J* = 4.0 Hz), 43.07 (d, *J* = 4.2 Hz), 31.60 (d, *J* = 11.1 Hz), 31.35 (d, *J* = 11.5 Hz), 24.07 (d, *J* = 1.6 Hz), 23.98 (d, *J* = 1.7 Hz), 23.11 (d, *J* = 6.6 Hz), 23.03 (d, *J* = 6.6 Hz), 20.20 (s), 19.01 (s), 18.66 (s), 17.91 (s).; ^{31}P NMR (162 MHz, CD_3CN) δ 21.93, 21.71 ; HRMS (TOF ES+) m/z calcd $C_{31}H_{42}N_3O_4P$ [(M + H)$^+$], 552.2912; found, 552.2971

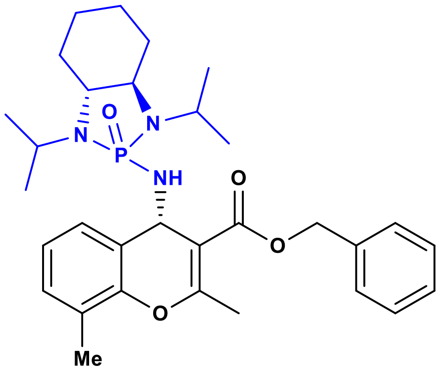

benzyl (S)-4-(((3aR,7aR)-1,3-diisopropyl-2-oxidooctahydrobenzo[d][1,3,2]diazaphosphol-2-yl)amino)-2,8-dimethyl-4H-chromene-3-carboxylate (3i). White solid, 0.407 g, 74% yield; mp 130 °C; $[\alpha]_D^{25}$ -4.3 (c 3, CH_3OH); ^1H NMR (400 MHz, CD_3CN) δ 7.50 – 7.47 (m, 1H), 7.45 (d, *J* = 0.5 Hz, 1H), 7.40 – 7.34 (m, 1H), 7.31 (dt, *J* = 5.5, 2.2 Hz, 1H), 7.09 (ddd, *J* = 7.5, 1.7, 0.8 Hz, 1H), 7.01 (t, *J* = 7.5 Hz, 1H), 5.38 (dd, *J* = 9.5, 7.2 Hz, 1H), 5.31 (d, *J* = 12.8 Hz, 1H), 5.08 (d, *J* = 12.8 Hz, 1H), 3.35 (tt, *J* = 13.8, 6.9 Hz, 1H), 3.15 (tt, *J* = 13.8, 6.9 Hz, 1H), 2.86 (t, *J* = 10.1 Hz, 1H), 2.79 – 2.65 (m, 1H), 2.57 (dd, *J* = 13.3, 6.5 Hz, 1H), 2.42 (s, 2H), 2.27 (s, 2H), 1.91 (dt, *J* = 4.9, 2.5 Hz, 1H), 1.61 (d, *J* = 10.6 Hz, 1H), 1.29 – 1.14 (m, 2H), 1.03 (d, *J* = 6.9 Hz, 2H), 0.97 (dd, *J* = 6.9, 3.8 Hz, 3H), 0.65 (d, *J* = 6.9 Hz, 2H).; ^{13}C NMR (101 MHz, CD_3CN) δ 167.01 (s), 162.39 (s), 149.00 (s), 136.56 (s), 129.13 (s), 128.59 (s), 128.06 (s), 126.90 (s), 125.59 (s), 124.97 (s), 123.86 (s), 106.48 (s), 106.40 (s), 66.01 (s), 58.96 (d, *J* = 10.4 Hz), 58.58 (d, *J* = 10.1 Hz), 45.29 (s), 43.46 (d, *J* = 4.0 Hz), 43.05 (d, *J* = 4.2 Hz), 31.51 (d, *J* = 11.1 Hz), 31.33 (d, *J* = 11.4 Hz), 24.09 (d, *J* = 1.6 Hz), 23.99 (d, *J* = 1.6 Hz), 23.12 (d, *J* = 6.6 Hz), 22.98 (d, *J* = 6.7 Hz), 19.09 (s), 18.71 (s), 17.96 (d, *J* = 0.6 Hz), 14.75 (s).; ^{31}P NMR (162 MHz, CD_3CN) δ 21.80 ; HRMS (TOF ES+) m/z calcd $C_{31}H_{42}N_3O_4P$ [(M + H)$^+$], 552.2912; found, 552.2974

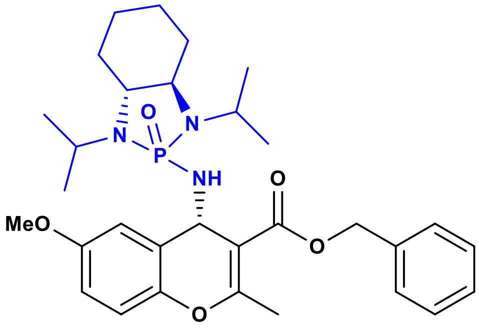

benzyl (S)-4-(((3aR,7aR)-1,3-diisopropyl-2-oxidooctahydrobenzo[d][1,3,2]diazaphosphol-2-yl)amino)-6-methoxy-2-methyl-4H-chromene-3-carboxylate (3j). White solid, 0.397 g, 70% yield; mp 141 °C; $[\alpha]_D^{25}$ -15.6 (c 0.65, CH_3OH); ^1H NMR (400 MHz, CD_3CN) δ 7.46 (d, *J* = 7.7 Hz, 2H), 7.36 (t, *J* = 7.4 Hz, 2H), 7.31 (d, *J* = 7.3 Hz, 1H), 7.28 (d, *J* = 3.1 Hz, 1H), 6.96 (d, *J* = 8.9 Hz, 1H), 6.79 (dd, *J* = 9.0, 3.0 Hz, 1H), 5.38 (dd, *J* = 9.4, 6.9 Hz, 1H), 5.32 (d, *J* = 12.7 Hz, 1H), 5.07 (d, *J* = 12.8 Hz, 1H), 3.74 (s, 3H), 3.43 – 3.28 (m, 1H), 3.20 (tt, *J* = 13.4, 6.8 Hz, 1H), 2.93 (t, *J* = 10.1 Hz, 1H), 2.77 – 2.66 (m, 1H), 2.63 – 2.49 (m, 1H), 2.37 (s, 3H), 1.91 (dt, *J* = 5.0, 2.5 Hz, 3H), 1.61 (d, *J* = 12.1 Hz, 2H), 1.32 – 1.16 (m, 4H), 1.12 – 0.84 (m, 10H), 0.68 (d, *J* = 6.9 Hz, 3H).; ^{13}C NMR (101 MHz, CD_3CN) δ 167.06 (s), 162.46 (s), 156.21 (s), 144.61 (s), 136.57 (s), 128.59 (s), 128.02 (s), 126.58 (s), 116.79 (s), 114.89 (s), 112.39 (s), 105.27 (s), 105.19 (s), 65.97 (s), 59.04 (d, *J* = 10.3 Hz), 58.54 (d, *J* = 10.1 Hz), 55.37 (s), 45.48 (s), 43.49 (d, *J* = 3.7 Hz), 43.12 (d, *J* = 3.9 Hz), 31.55 (d, *J* = 11.1 Hz), 31.28 (d, *J* = 11.4 Hz), 24.05 (d, *J* = 1.3 Hz), 23.97 (d, *J* = 1.4 Hz), 22.98 (d, *J* = 12.2 Hz), 22.98 (s), 19.08 (s), 18.60 (s), 17.94 (s).; ^{31}P NMR (162 MHz, CD_3CN) δ 21.85, 21.78 ; HRMS (TOF ES+) m/z calcd $C_{31}H_{42}N_3O_5P$ [(M + H)$^+$], 568.2862; found, 568.2914

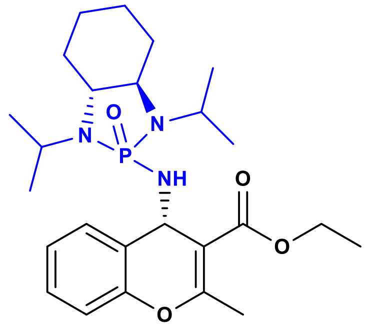

ethyl (S)-4-(((3aR,7aR)-1,3-diisopropyl-2-oxidooctahydrobenzo[d][1,3,2]diazaphosphol-2-yl)amino)-2-methyl-4H-chromene-3-carboxylate (3m). White solid, 0.380 g, 80% yield; mp 117 °C; $[\alpha]_D^{25}$ -35.3 (c 0.45, CH_3OH); ^1H NMR (400 MHz, CD_3CN) δ 7.68 (dd, *J* = 7.6, 1.4 Hz, 1H), 7.29 – 7.20 (m, 1H), 7.17 – 7.09 (m, 1H), 7.02 (d, *J* = 8.1 Hz, 1H), 5.35 (dd, *J* = 9.5, 7.2 Hz, 1H), 4.25 (dq, *J* = 11.0, 7.2 Hz, 1H), 4.10 (dq, *J* = 11.4, 7.4 Hz, 1H), 3.51 – 3.27 (m, 1H), 3.28 – 3.00 (m, 1H), 2.91 (t, *J* = 10.1 Hz, 1H), 2.77 – 2.65 (m, 1H), 2.67 – 2.53 (m, 1H), 2.39 (s, 3H), 1.99 – 1.88 (m, 5H), 1.78 – 1.50 (m, 3H), 1.28 (t, *J* = 7.1 Hz, 5H), 1.23 (dd, *J* = 6.5, 3.3 Hz, 3H), 1.06 (d, *J* = 6.9 Hz, 7H), 0.98 (d, *J* = 6.9 Hz, 5H), 0.65 (d, *J* = 6.9 Hz, 4H).; ^{13}C NMR (101 MHz, CD_3CN) δ ^{13}C NMR (101 MHz, ACETONITRILE-D3) δ 167.14 (s), 161.65 (s), 150.62 (s), 129.42 (s), 128.03 (s), 126.00 (s), 124.28 (s), 115.72 (s), 106.58 (s), 60.37 (s), 59.01 (d, *J* = 10.2 Hz), 58.54 (d, *J* = 10.2 Hz), 45.00 (s), 43.44 (d, *J* = 3.7 Hz), 43.04 (d, *J* = 4.0 Hz), 31.56 (d, *J* = 11.1 Hz), 31.30 (d, *J* = 11.4 Hz), 24.06 (d, *J* = 1.3 Hz), 23.97 (d, *J* = 1.4 Hz), 23.07 (d, *J* = 6.5 Hz), 22.95 (d, *J* = 6.6 Hz), 18.85 (s), 18.63 (s), 17.96 (s), 13.65 (s).; ^{31}P NMR (162 MHz, CD_3CN) δ 21.89, 21.78 ; HRMS (TOF ES+) m/z calcd $C_{25}H_{38}N_3O_4P$ [(M + H)$^+$], 476.2599; found, 476.2661

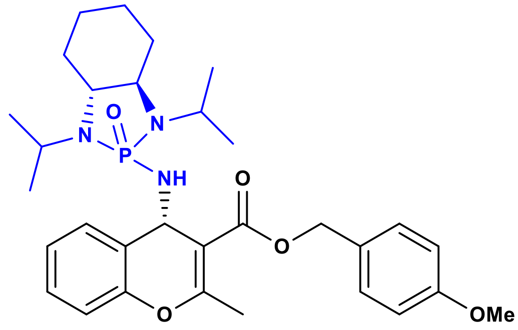

4-methoxybenzyl (S)-4-(((3aR,7aR)-1,3-diisopropyl-2-oxidooctahydrobenzo[d][1,3,2]diazaphosphol-2-yl)amino)-2-methyl-4H-chromene-3-carboxylate (3n). White solid, 0.425 g, 75% yield; mp 171 °C; $[\alpha]_D^{25}$ -2 (c 1.1, CH_3OH) ^1H NMR (400 MHz, CD_3CN) δ 7.66 (dd, *J* = 7.6, 1.6 Hz, 1H), 7.38 (d, *J* = 8.8 Hz, 1H), 7.23 (ddd, *J* = 8.2, 7.4, 1.7 Hz, 1H), 7.12 (td, *J* = 7.4, 1.2 Hz, 1H), 7.02 (dd, *J* = 8.2, 1.1 Hz, 1H), 6.90 (d, *J* = 8.7 Hz, 1H), 5.37 (dd, *J* = 9.5, 7.4 Hz, 1H), 5.24 (d, *J* = 12.3 Hz, 1H), 5.00 (d, *J* = 12.3 Hz, 1H), 3.75 (s, 1H), 3.42 – 3.24 (m, 1H), 3.14 (tt, *J* = 13.7, 6.9 Hz, 1H), 2.92 (t, *J* = 10.2 Hz, 1H), 2.80 – 2.62 (m, 1H), 2.56 (td, *J* = 10.4, 3.2 Hz, 1H), 1.91 (dt, *J* = 4.9, 2.5 Hz, 1H), 1.62 (t, *J* = 9.3 Hz, 1H), 1.20 (dt, *J* = 14.3, 5.9 Hz, 1H), 1.20 (ddd, *J* = 14.3, 10.9, 5.9 Hz, 1H), 1.08 – 0.87 (m, 3H), 0.64 (d, *J* = 6.9 Hz, 1H).; ^{13}C NMR (101 MHz, CD_3CN) δ 167.05 (s), 161.97 (s), 159.67 (s), 150.59 (s), 130.01 (s), 129.96 (s), 129.44 (s), 128.47 (s), 128.09 (s), 125.94 (s), 115.72 (s), 113.92 (s), 113.87 (s), 106.53 (s), 106.45 (s), 65.86 (d, *J* = 1.3 Hz), 58.99 (d, *J* = 10.1 Hz), 58.57 (d, *J* = 9.9 Hz), 54.99 (d, *J* = 8.5 Hz), 45.08 (s), 45.02 (d, *J* = 1.0 Hz), 43.48 (d, *J* = 3.6 Hz), 43.08 (d, *J* = 3.4 Hz), 31.55 (d, *J* = 11.0 Hz), 31.33 (d, *J* = 11.5 Hz), 24.02 (d, *J* = 10.4 Hz), 23.12 (d, *J* = 6.5 Hz), 18.97 (d, *J* = 2.6 Hz), 18.65 (s), 17.94 (s).; ^{31}P NMR (162 MHz, CD_3CN) δ 21.87, 21.70 ; HRMS (TOF ES+) m/z calcd $C_{31}H_{42}N_3O_5P$ [(M + H)$^+$], 568.2862; found, 568.2921

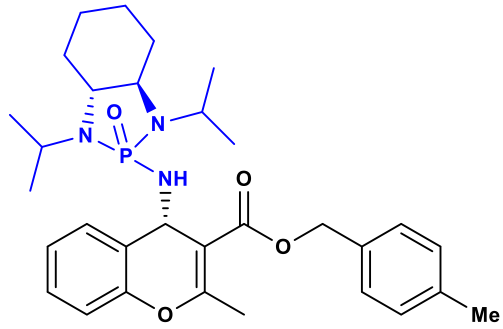

4-methylbenzyl (S)-4-(((3aR,7aR)-1,3-diisopropyl-2-oxidooctahydrobenzo[d][1,3,2]diazaphosphol-2-yl)amino)-2-methyl-4H-chromene-3-carboxylate (3o). White solid, 0.413 g, 75% yield; mp 167 °C; $[\alpha]_D^{25}$ 4.8 (c 0.6, CH_3OH) ^1H NMR (400 MHz, CD_3CN) δ 7.67 (dd, *J* = 7.6, 1.4 Hz, 1H), 7.34 (d, *J* = 8.0 Hz, 2H), 7.28 – 7.20 (m, 1H), 7.14 (ddd, *J* = 10.9, 8.5, 4.5 Hz, 4H), 7.02 (d, *J* = 8.1 Hz, 1H), 5.45 – 5.34 (m, 1H), 5.27 (d, *J* = 12.5 Hz, 1H), 5.03 (d, *J* = 12.5 Hz, 2H), 3.41 – 3.28 (m, 2H), 3.15 (tt, *J* = 13.8, 6.9 Hz, 1H), 2.93 (t, *J* = 10.0 Hz, 1H), 2.78 – 2.64 (m, 1H), 2.61 – 2.49 (m, 1H), 2.38 (s, 3H), 2.30 (s, 4H), 1.91 (dt, *J* = 5.0, 2.5 Hz, 4H), 1.62 (d, *J* = 10.3 Hz, 2H), 1.21 – 1.15 (m, 3H), 1.09 – 0.92 (m, 12H), 0.65 (d, *J* = 6.9 Hz, 3H).; ^{13}C NMR (101 MHz, CD_3CN) δ 167.02 (s), 162.05 (s), 150.58 (s), 137.99 (s), 133.49 (s), 129.45 (s), 129.17 (s), 129.15 (s), 128.26 (s), 128.05 (s), 125.97 (s), 124.32 (s), 115.71 (s), 106.48 (s), 106.40 (s), 65.99 (s), 58.98 (d, *J* = 10.3 Hz), 58.55 (d, *J* = 10.2 Hz), 45.04 (s), 43.46 (d, *J* = 4.1 Hz), 43.05 (d, *J* = 4.1 Hz), 31.57 (d, *J* = 11.1 Hz), 31.34 (d, *J* = 11.5 Hz), 24.07 (d, *J* = 1.7 Hz), 23.97 (d, *J* = 1.7 Hz), 23.10 (d, *J* = 6.6 Hz), 22.96 (d, *J* = 6.7 Hz), 20.27 (s), 18.97 (s), 18.63 (s), 17.92 (s).; ^{31}P NMR (162 MHz, CD_3CN) δ 21.86, 21.70 ; HRMS (TOF ES+) m/z calcd $C_{31}H_{42}N_3O_4P$ [(M + H)$^+$], 552.2912; found, 552.2972

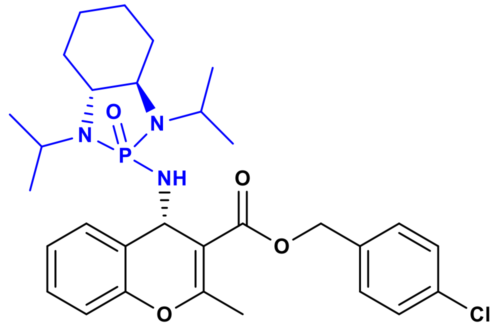

4-chlorobenzyl (S)-4-(((3aR,7aR)-1,3-diisopropyl-2-oxidooctahydrobenzo[d][1,3,2]diazaphosphol-2-yl)amino)-2-methyl-4H-chromene-3-carboxylate (3p). White solid, 0.405 g, 71% yield; mp 190 °C; $[\alpha]_D^{25}$ -12.7 (c 0.7, CH_3OH) ^1H NMR (400 MHz, CD_3CN) δ 7.68 (d, *J* = 8.3 Hz, 1H), 7.45 (d, *J* = 7.2 Hz, 2H), 7.36 (t, *J* = 7.2 Hz, 2H), 7.34 – 7.28 (m, 2H), 7.19 – 7.12 (m, 1H), 7.10 (d, *J* = 1.9 Hz, 1H), 5.41 – 5.34 (m, 1H), 5.32 (d, *J* = 12.7 Hz, 1H), 5.08 (d, *J* = 12.7 Hz, 1H), 3.42 – 3.24 (m, 1H), 3.13 (tt, *J* = 13.5, 6.8 Hz, 1H), 3.02 (t, *J* = 10.1 Hz, 1H), 2.78 – 2.65 (m, 1H), 2.62 – 2.50 (m, 1H), 2.38 (s, 3H), 1.91 (ddd, *J* = 4.9, 2.5, 0.6 Hz, 3H), 1.62 (d, *J* = 9.7 Hz, 1H), 1.22 (d, *J* = 8.2 Hz, 4H), 1.07 – 0.93 (m, 11H), 0.69 (d, *J* = 6.9 Hz, 3H).; ^{13}C NMR (101 MHz, CD_3CN) δ 166.73 (s), 161.86 (s), 151.08 (s), 136.43 (s), 132.64 (s), 130.92 (s), 128.60 (s), 128.10 (s), 124.91 (s), 124.37 (s), 115.85 (s), 106.68 (s), 106.60 (s), 66.17 (s), 58.99 (d, *J* = 10.2 Hz), 58.57 (d, *J* = 10.1 Hz), 44.61 (s), 43.49 (d, *J* = 3.6 Hz), 43.14 (d, *J* = 3.9 Hz), 31.57 (d, *J* = 11.0 Hz), 31.29 (d, *J* = 11.5 Hz), 24.06 (d, *J* = 1.4 Hz), 23.96 (d, *J* = 1.4 Hz), 23.04 (d, *J* = 6.6 Hz), 22.95 (d, *J* = 6.5 Hz), 18.85 (s), 18.64 (s), 17.92 (s).; ^{31}P NMR (162 MHz, CD_3CN) δ 21.87, 21.68 ; HRMS (TOF ES+) m/z calcd $C_{30}H_{39}ClN_3O_4P$ [(M + H)$^+$], 572.2366; found, 572.2427

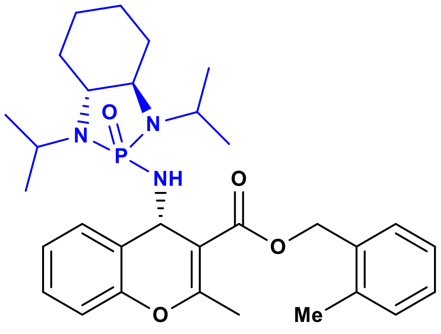

2-methylbenzyl (S)-4-(((3aR,7aR)-1,3-diisopropyl-2-oxidooctahydrobenzo[d][1,3,2]diazaphosphol-2-yl)amino)-2-methyl-4H-chromene-3-carboxylate (3q). White solid, 0.386 g, 70% yield; mp 152 °C; $[\alpha]_D^{25}$ -31.7 (c 0.7, CH_3OH) ^{1}H NMR (400 MHz, CD_3CN) δ 7.52 – 7.42 (m, 3H), 7.37 (ddd, *J* = 12.5, 5.2, 2.3 Hz, 2H), 7.31 (dt, *J* = 9.8, 4.3 Hz, 1H), 7.11 – 6.98 (m, 1H), 6.91 (d, *J* = 8.3 Hz, 1H), 5.37 (dd, *J* = 9.6, 7.5 Hz, 1H), 5.31 (d, *J* = 12.8 Hz, 1H), 5.07 (d, *J* = 12.7 Hz, 1H), 3.33 (tt, *J* = 14.0, 7.0 Hz, 1H), 3.28 – 3.10 (m, 1H), 2.91 (t, *J* = 10.0 Hz, 1H), 2.76 – 2.63 (m, 1H), 2.63 – 2.49 (m, 1H), 2.37 (s, 3H), 2.28 (s, 3H), 1.91 (dt, *J* = 4.9, 2.5 Hz, 3H), 1.61 (d, *J* = 10.1 Hz, 2H), 1.27 – 1.14 (m, 3H), 1.09 – 0.98 (m, 7H), 0.96 (d, *J* = 6.9 Hz, 3H), 0.67 (d, *J* = 6.9 Hz, 3H).; ^{13}C NMR (101 MHz, CD_3CN) δ 167.11 (s), 162.07 (s), 148.43 (s), 136.57 (s), 133.91 (s), 129.43 (s), 129.38 (s), 128.61 (s), 128.58 (s), 128.56 (s), 128.02 (s), 125.54 (s), 115.49 (s), 106.09 (s), 106.01 (s), 65.96 (s), 58.97 (d, *J* = 10.3 Hz), 58.55 (d, *J* = 10.1 Hz), 45.09 (s), 43.47 (d, *J* = 3.8 Hz), 43.14 (d, *J* = 4.0 Hz), 31.62 (d, *J* = 11.1 Hz), 31.33 (d, *J* = 11.5 Hz), 24.06 (d, *J* = 1.4 Hz), 23.97 (d, *J* = 1.4 Hz), 23.09 (d, *J* = 6.6 Hz), 22.97 (d, *J* = 6.8 Hz), 19.92 (s), 19.04 (s), 18.70 (s), 17.87 (s).; ^{31}P NMR (162 MHz, CD_3CN) δ 21.94, 21.66 ; HRMS (TOF ES+) m/z calcd $C_{31}H_{42}N_3O_4P$ [(M + H)$^+$], 552.2912; found, 552.2966

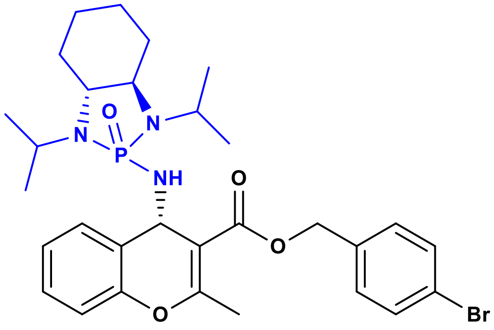

4-bromobenzyl (S)-4-(((3aR,7aR)-1,3-diisopropyl-2-oxidooctahydrobenzo[d][1,3,2]diazaphosphol-2-yl)amino)-2-methyl-4H-chromene-3-carboxylate (3r). White solid, 0.430 g, 69% yield; mp 168 °C; $[\alpha]_D^{25}$ -27.6 (c 0.8, CH_3OH) ^{1}H NMR (400 MHz, CD_3CN) δ 7.49 – 7.42 (m, 1H), 7.40 – 7.29 (m, 1H), 5.42 – 5.29 (m, 1H), 5.08 (d, *J* = 12.8 Hz, 1H), 3.32 (pd, *J* = 13.4, 7.4 Hz, 1H), 3.08 (ddd, *J* = 36.0, 17.0, 8.5 Hz, 1H), 2.76 – 2.66 (m, 1H), 2.58 (dd, *J* = 13.5, 6.2 Hz, 1H), 2.39 (s, 1H), 1.91 (dt, *J* = 4.9, 2.5 Hz, 1H), 1.62 (d, *J* = 10.4 Hz, 1H), 1.28 – 1.17 (m, 1H), 1.03 (d, *J* = 6.9 Hz, 2H), 0.98 (dd, *J* = 6.9, 3.6 Hz, 2H), 0.71 (d, *J* = 6.9 Hz, 1H).^{13}C NMR (101 MHz, CD_3CN) δ 166.99 (s), 162.20 (s), 150.58 (d, *J* = 1.2 Hz), 136.55 (s), 129.45 (s), 128.59 (s), 128.06 (s), 125.95 (s), 124.35 (s), 115.74 (s), 106.39 (d, *J* = 7.9 Hz), 66.03 (s), 58.99 (d, *J* = 10.2 Hz), 58.56 (d, *J* = 10.1 Hz), 45.05 (s), 43.46 (d, *J* = 3.7 Hz), 43.06 (d, *J* = 3.9 Hz), 31.45 (dd, *J* = 23.0, 11.3 Hz), 24.02 (dd, *J* = 9.7, 1.4 Hz), 23.04 (dd, *J* = 12.7, 6.6 Hz), 19.02 (s), 18.65 (s), 17.91 (s).^{31}P NMR (162 MHz, CD_3CN) δ 21.87, 21.68 ; HRMS (TOF ES+) m/z calcd $C_{30}H_{39}BrN_3O_4P$ [(M + H)$^+$], 616.1861; found, 616.1953

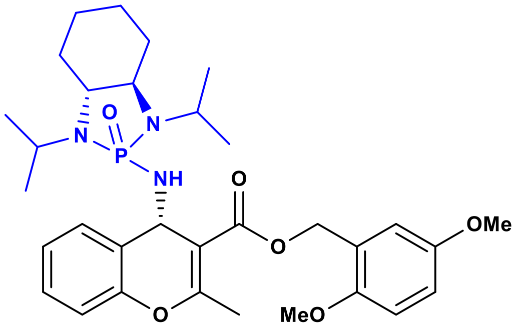

2,5-dimethoxybenzyl (S)-4-(((3aR,7aR)-1,3-diisopropyl-2-oxidooctahydrobenzo[d][1,3,2]diazaphosphol-2-yl)amino)-2-methyl-4H-chromene-3-carboxylate (3s). White solid, 0.370 g, 62% yield; mp 190 °C; $[\alpha]_D^{25}$ -51.7 (c 1, CH_3OH) ^{1}H NMR (400 MHz, CH_3CD) δ 7.70 (dd, *J* = 7.6, 1.7 Hz, 1H), 7.28 – 7.20 (m, 1H), 7.19 – 7.09 (m, 1H), 7.08 – 6.98 (m, 2H), 6.84 (d, *J* = 3.1 Hz, 1H), 5.47 – 5.37 (m, 1H), 5.28 (d, *J* = 13.1 Hz, 1H), 5.05 (d, *J* = 13.2 Hz, 1H), 3.76 (s, 3H), 3.74 (s, 4H), 3.41 – 3.24 (m, 1H), 3.14 (tt, *J* = 13.7, 6.9 Hz, 1H), 2.90 (dd, *J* = 13.0, 8.4 Hz, 1H), 2.73 – 2.62 (m, 1H), 2.55 (td, *J* = 10.4, 3.1 Hz, 1H), 2.38 (d, *J* = 1.6 Hz, 3H), 1.91 (dt, *J* = 5.0, 2.5 Hz, 3H), 1.60 (d, *J* = 9.6 Hz, 2H), 1.28 – 1.16 (m, 4H), 1.12 – 0.82 (m, 13H), 0.65 (d, *J* = 6.9 Hz, 3H). ^{13}C NMR (101 MHz, CH_3CD) δ 166.90 (s), 161.82 (s), 153.46 (s), 151.25 (s), 150.38 (s), 129.35 (s), 127.89 (s), 125.77 (s), 125.17 (s), 124.15 (s), 115.55 (s), 115.06 (s), 113.48 (s), 111.65 (s), 106.23 (d, *J* = 7.7 Hz), 61.30 (s), 58.60 (dd, *J* = 42.3, 10.1 Hz), 58.32 – 58.20 (m), 55.59 (s), 55.19 (s), 44.93 (s), 43.28 (s), 42.88 (d, *J* = 3.9 Hz), 31.29 (dd, *J* = 19.0, 11.1 Hz), 23.85 (d, *J* = 8.7 Hz), 22.87 (dd, *J* = 15.9, 6.5 Hz), 18.78 (s), 18.49 (s), 17.72 (s). ^{31}P NMR (162 MHz, CD_3CN) δ 21.85, 21.71; HRMS (TOF ES+) m/z calcd $C_{32}H_{44}N_3O_6P$ [(M + H)$^+$], 598.2967; found, 598.2951

Preparation of Crystal of compound 3a

5 mg of product 3a is placed in a shell vial and dissolved in acetonitrile. The slow evaporation of solvent over 3 days resulted the white crystal.

X-Ray Structure of Products 3a

General Data Collection

Data were collected on a Rigaku XtaLAB Synergy-*i* Kappa diffractometer equipped with a PhotonJet-*i* X-ray source operated at 50 W (50kV, 1 mA) to generate Cu Kα radiation (λ = 1.54178 Å) and a HyPix-6000HE HPC detector. Crystals were transferred from the vial and placed on a glass slide in type NVH immersion oil by Cargille. A Zeiss Stemi 305 microscope was used to identify a suitable specimen for X-ray diffraction from a representative sample of the material. The crystal and a small amount of the oil were collected on a MiTeGen 50 micron MicroLoop and transferred to the instrument where it was placed under a cold nitrogen stream (Oxford 700 series) maintained at 100K throughout the duration of the experiment. The sample was optically centered with the aid of a video camera to insure that no translations were observed as the crystal was rotated through all positions.

A unit cell collection was then carried out. After it was determined that the unit cell was not present in the CCDC database a data collection strategy was calculated by *CrysAlis^{Pro}*[1]. The crystal was measured for size, morphology, and color. These values are reported in the accompanying Li21_03_tables file.

Refinement Details

After data collection, the unit cell was re-determined using a subset of the full data collection. Intensity data were corrected for Lorentz, polarization, and background effects using the *CrysAlis^{Pro}*[1]. A numerical absorption correction was applied based on a Gaussian integration over a multifaceted crystal and followed by a semi-empirical correction for adsorption applied using the program *SCALE3 ABSPACK*[2]. The programs *SHELXT*[3] was used for the initial structure solution and *SHELXL*[4] was used for refinement of the structure. Both of these programs

were utilized within the OLEX2 software[5]. Hydrogen atoms bound to carbon atoms were located in the difference Fourier map and were geometrically constrained using the appropriate AFIX commands. The hydrogen atom bound to N1 (H1) was allowed to free refine its position while its Uiso value was set to ride on N1.

References:

1. CrysAlis^{Pro} (2018) Oxford Diffraction Ltd.
2. SCALE3 ABSPACK (2005) Oxford Diffraction Ltd.
3. Sheldrick, G. M. (2015) *Acta Crystallogr*., C**71**, 3-8.
4. Sheldrick, G. M. (2015) *Acta Crystallogr*., A**71**, 3-8.
5. Dolomanov, O. V.; Bourhis, . L. J.; Gildea, R. J.; Howard, J. A. K.; Puschmann. H. (2009) *J. Appl. Cryst*. **42**, 339-341.
6. van der Sluis, P.; Spek, A.L. (1990) *Acta Crystallogr*., **A46**, 194-201.

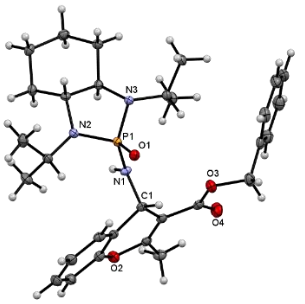

The thermal ellipsoids are represented at 50% probability. Carbon, hydrogen, nitrogen, oxygen and phosphorus atoms are represented by gray, white, light blue, red, and orange ellipsoids, respectively.

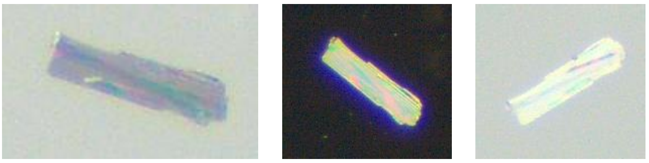

Crystal used for single crystal X-ray diffraction experiment.

Copies of NMRs of compounds 1a-1l

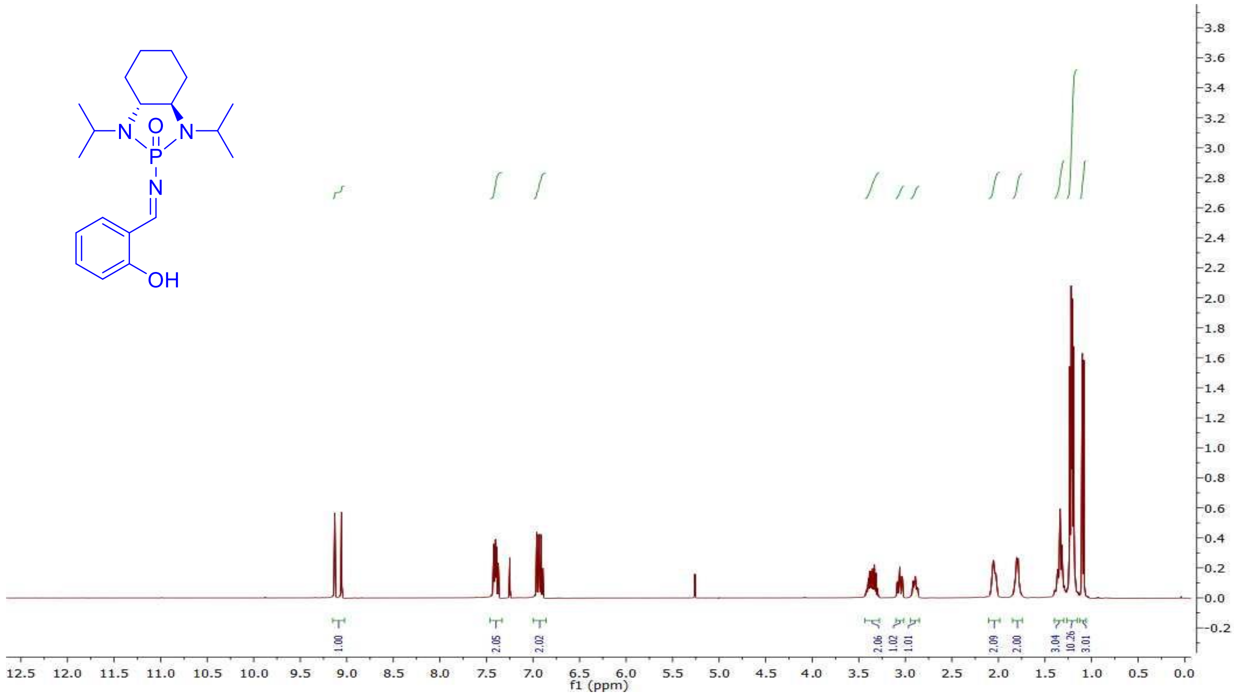

^{1}H NMR of imine **1a** ($CDCl_3$, 400 Hz)

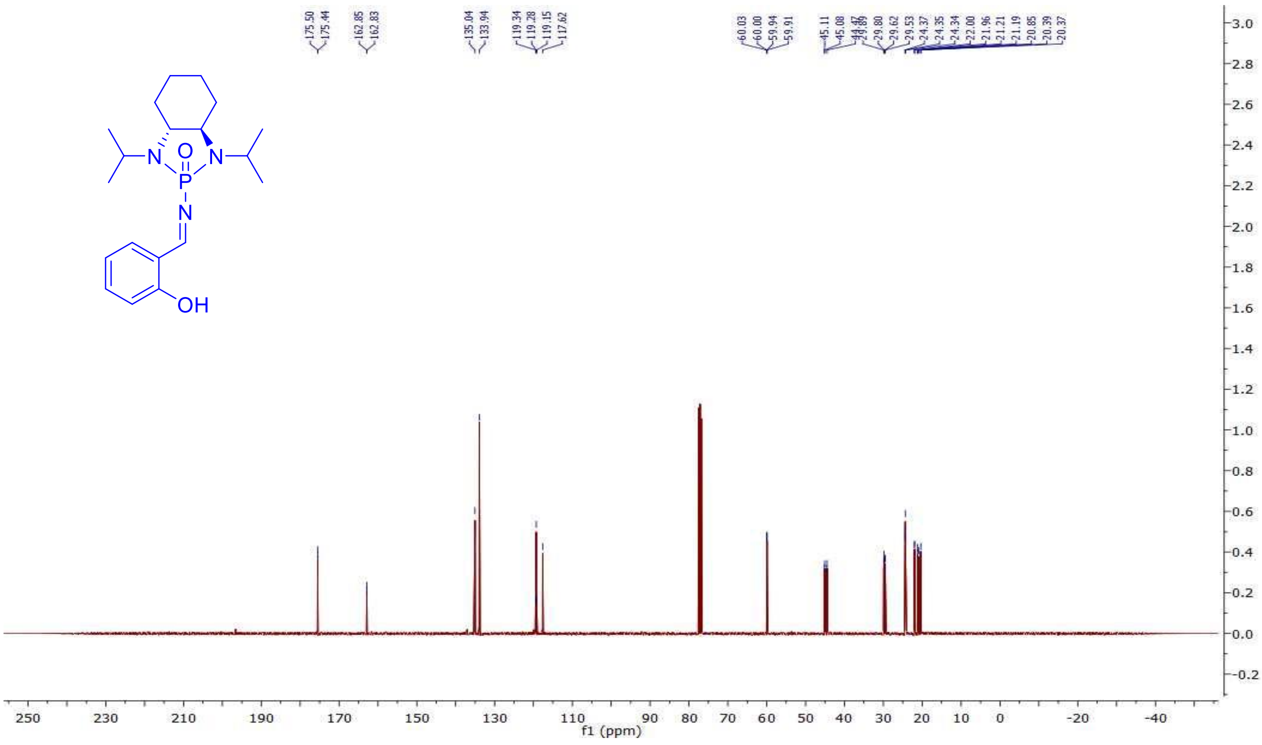

^{13}C NMR of imine **1a** ($CDCl_3$, 100 Hz)

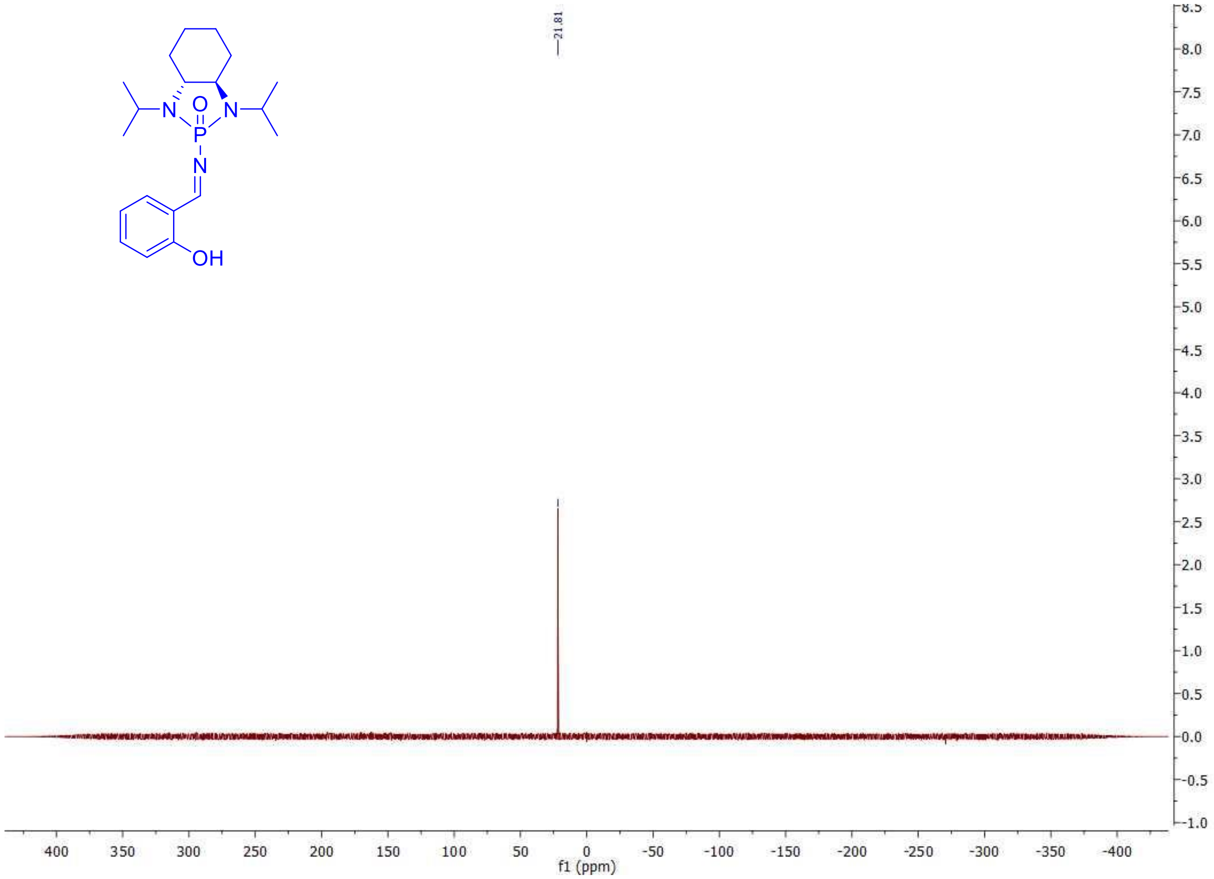

^{31}P NMR of imine **1a** ($CDCl_3$, 162 Hz)

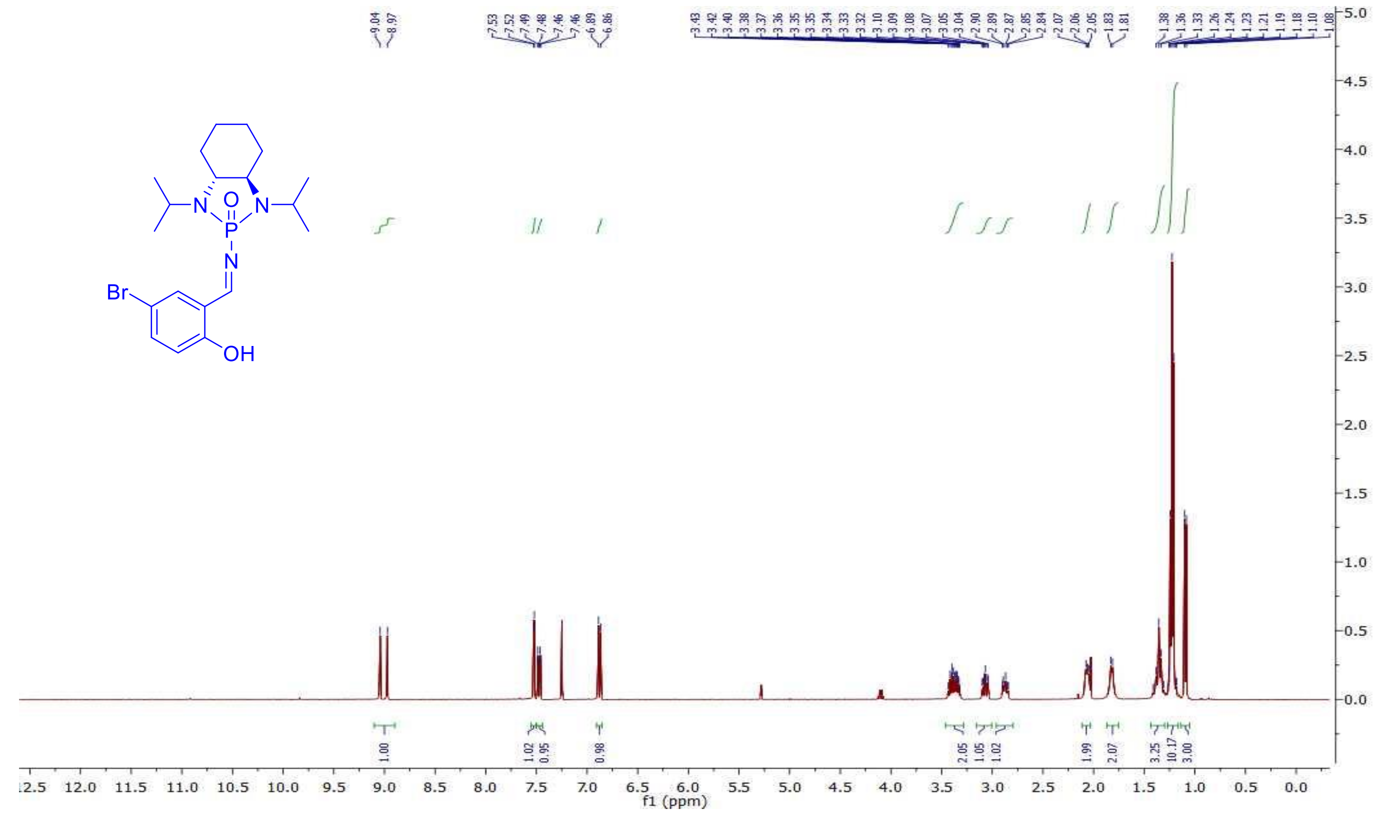

^{1}H NMR of imine **1b** ($CDCl_3$, 400 Hz)

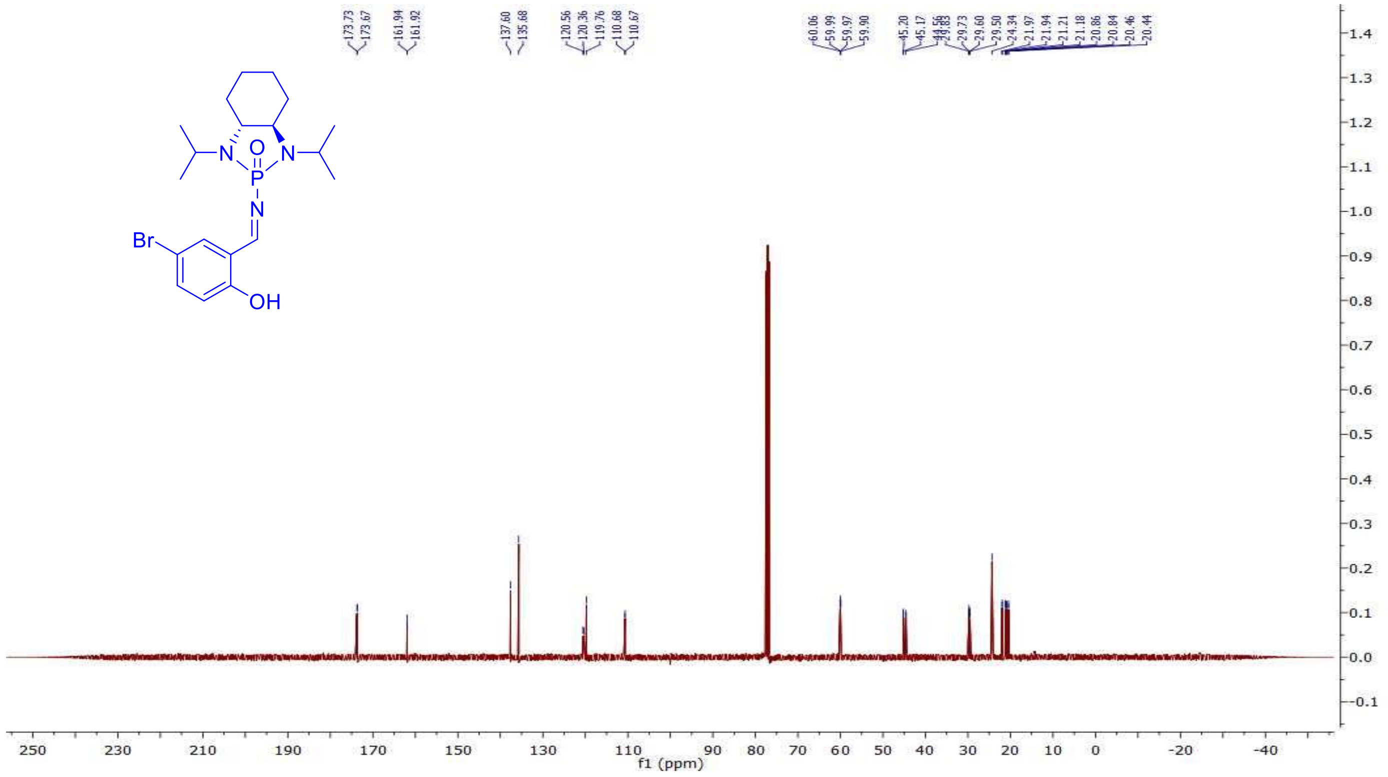

^{13}C NMR of imine **1b** ($CDCl_3$, 100 Hz)

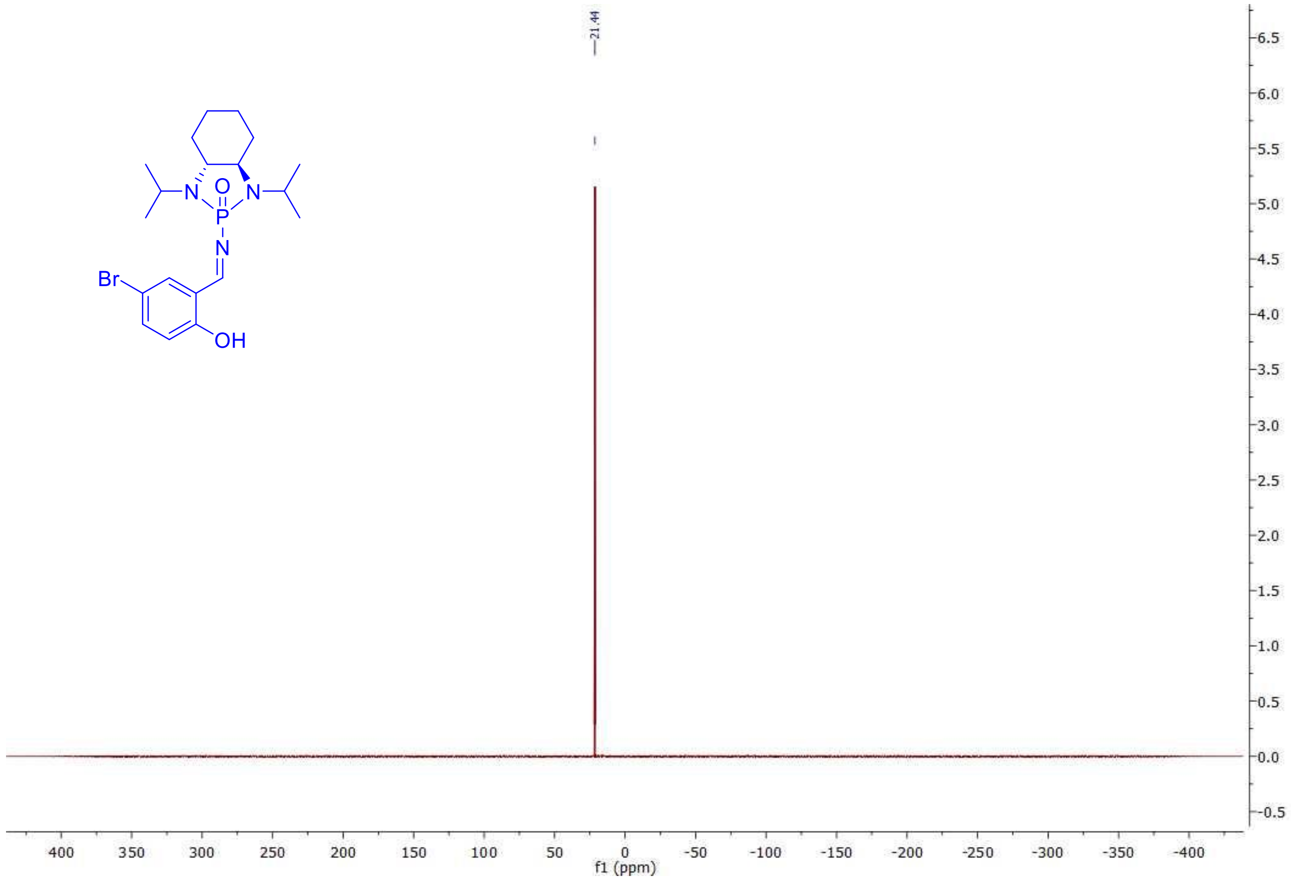

^{31}P NMR of imine **1b** ($CDCl_3$, 162 Hz)

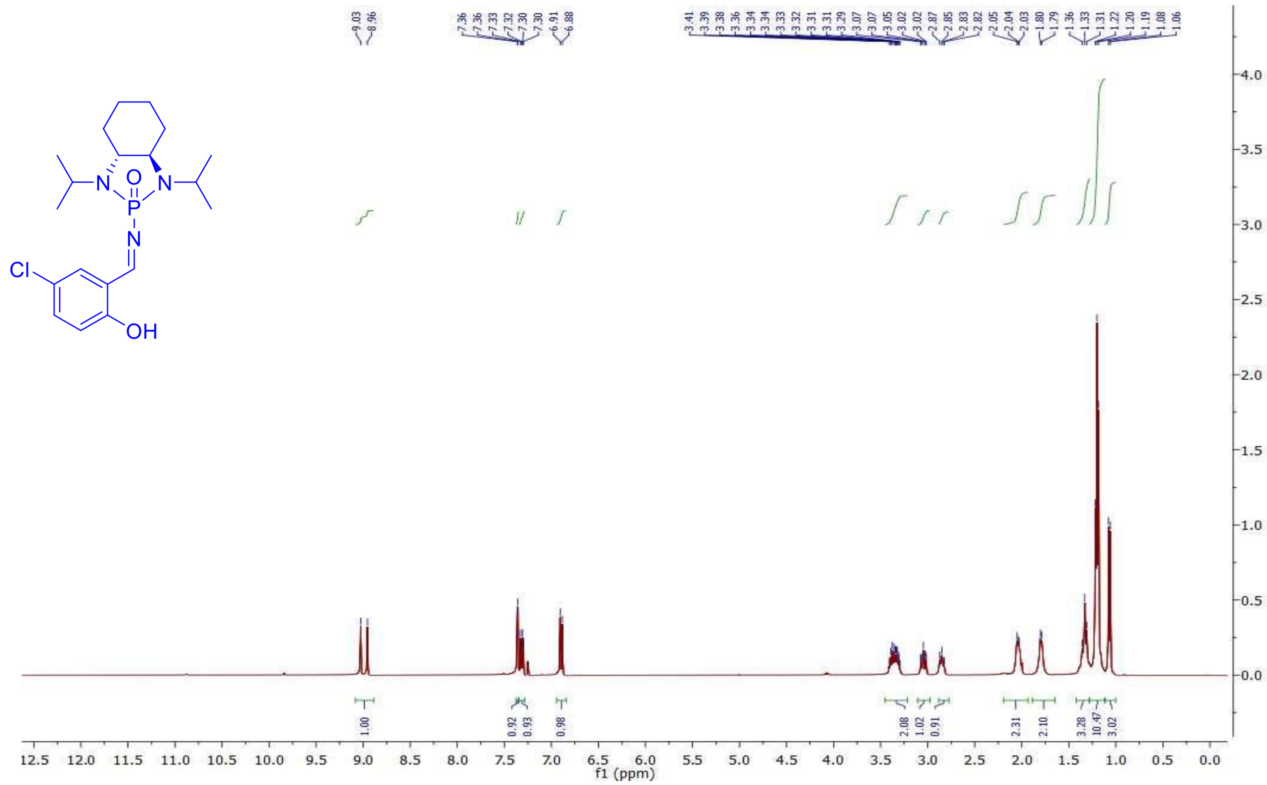

^{1}H NMR of imine **1c** ($CDCl_3$, 400 Hz)

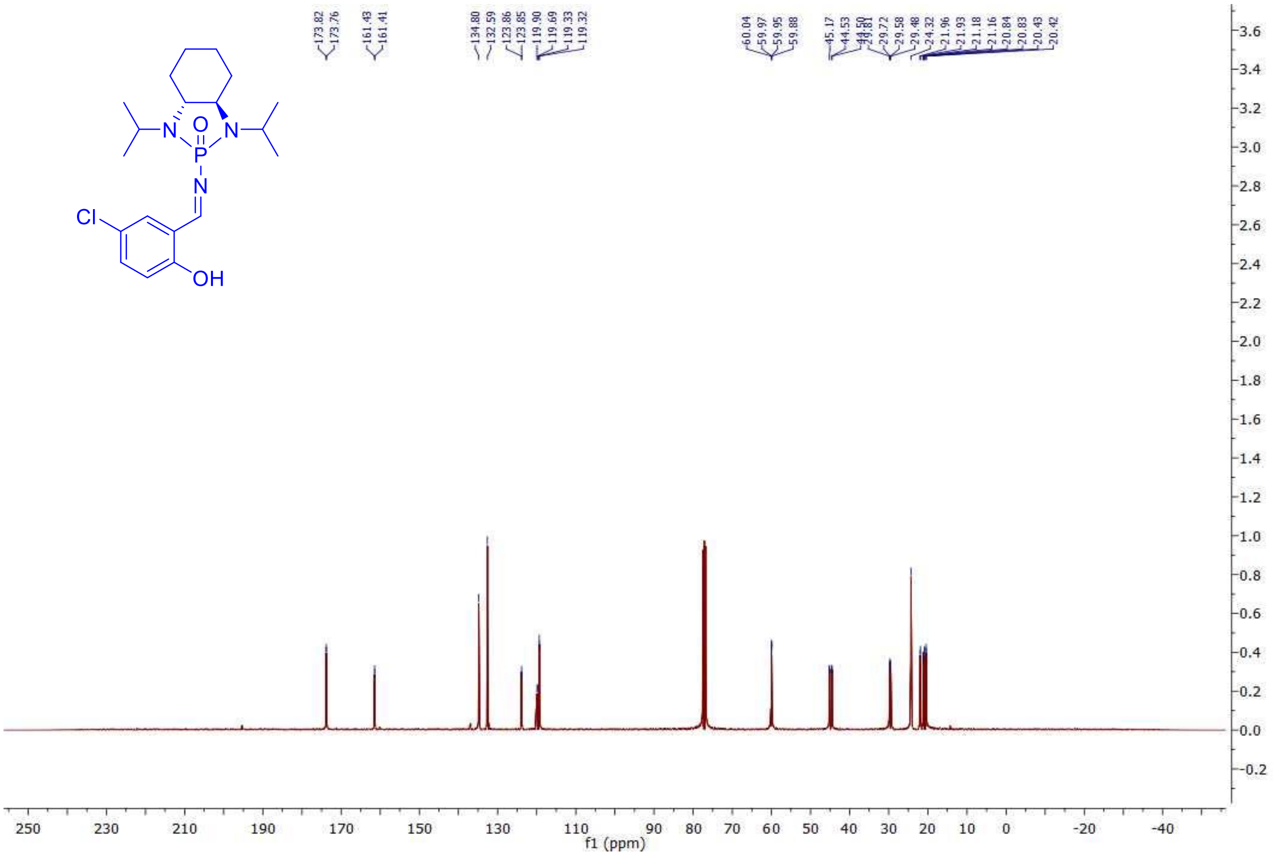

^{13}C NMR of imine **1c** ($CDCl_3$, 100 Hz)

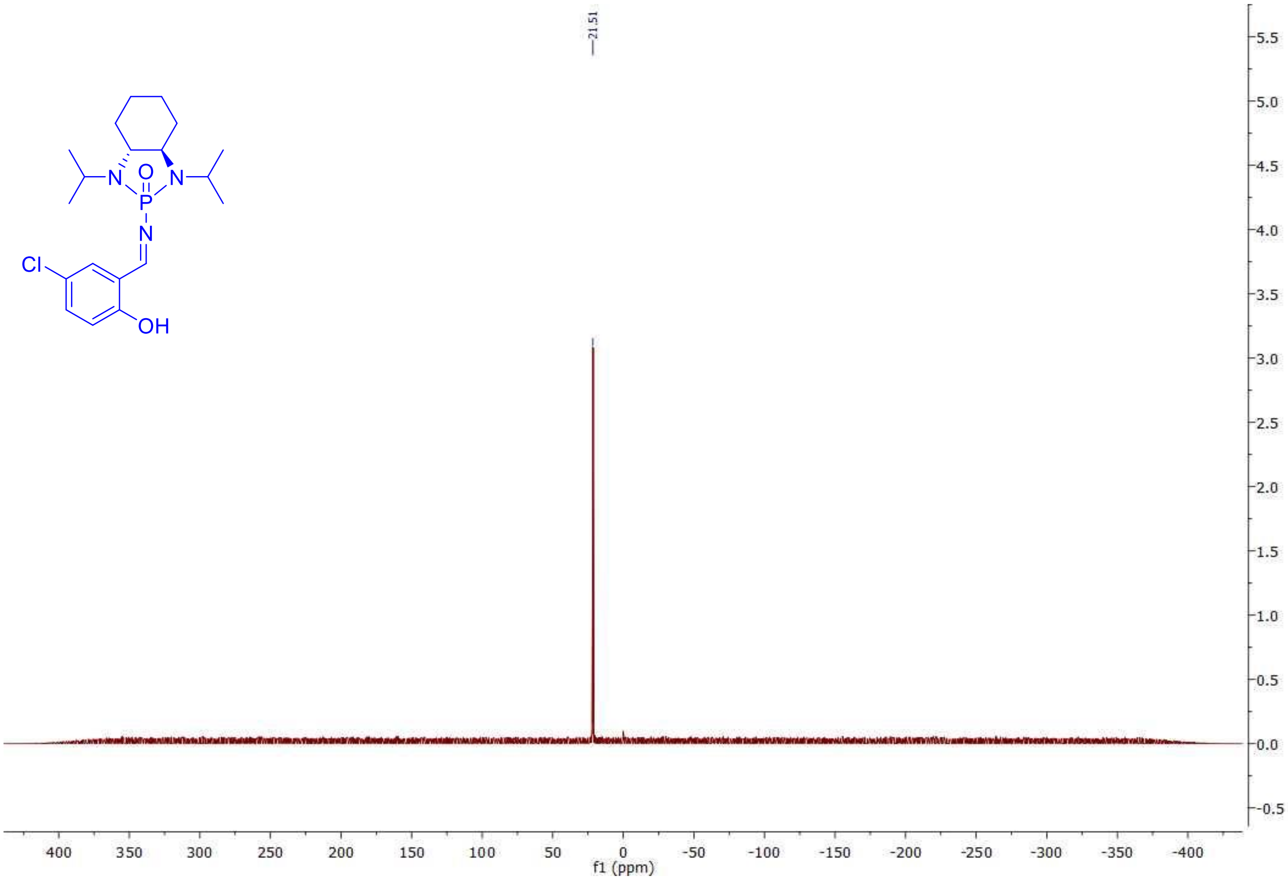

^{31}P NMR of imine **1c** ($CDCl_3$, 162 Hz)

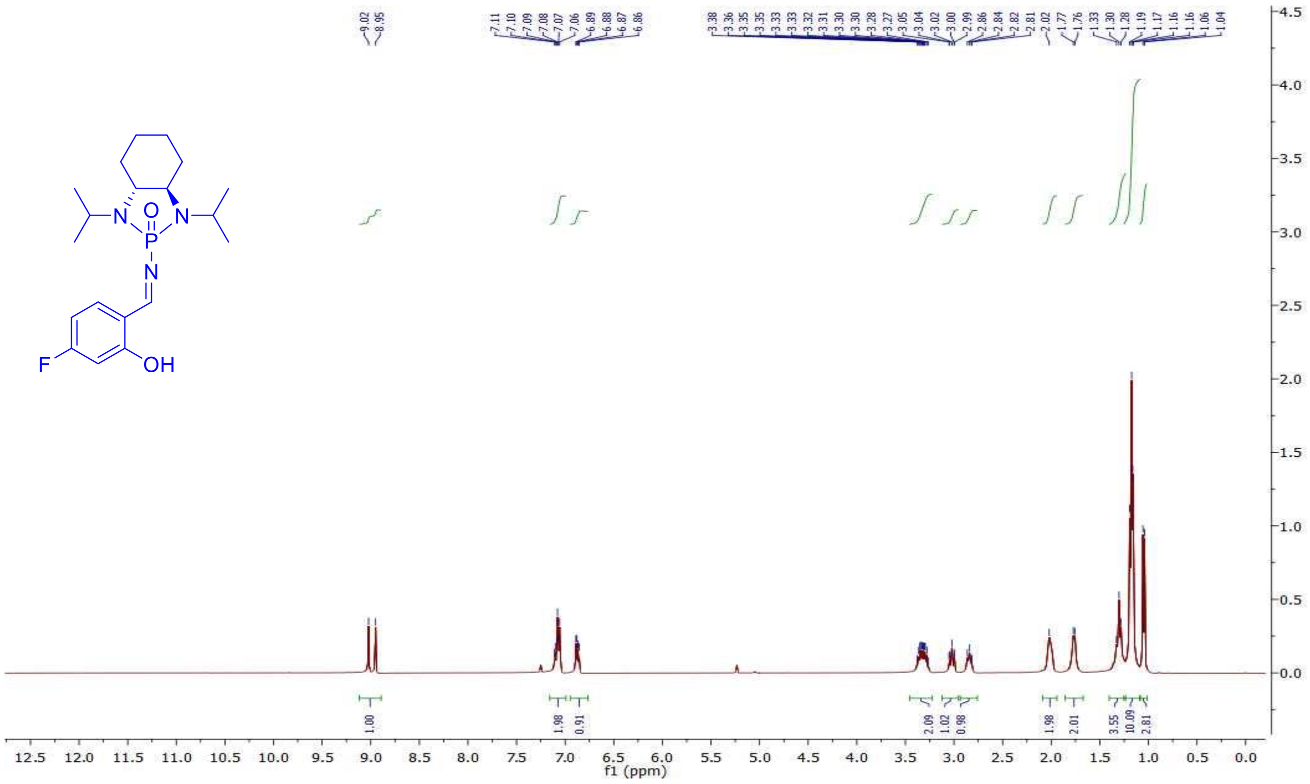

^{1}H NMR of imine **1d** ($CDCl_3$, 400 Hz)

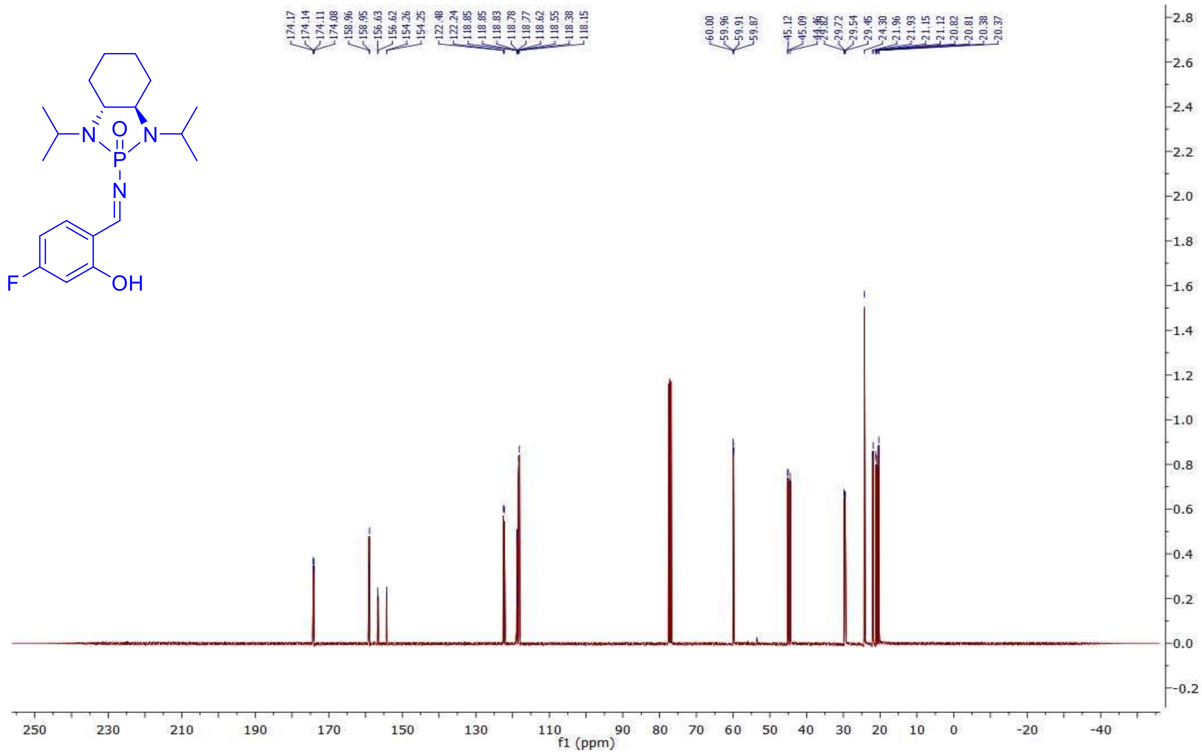

^{13}C NMR of imine **1d** ($CDCl_3$, 100 Hz)

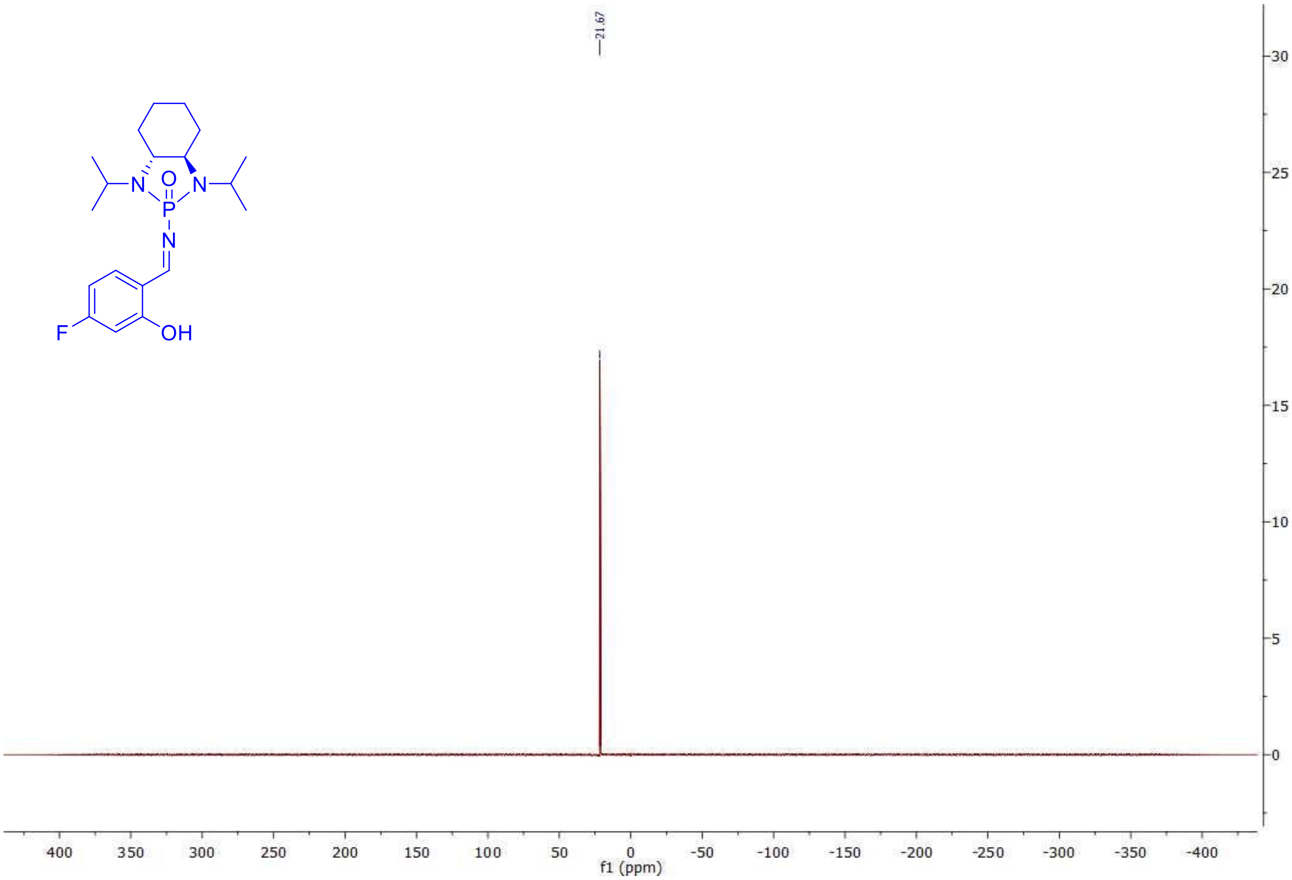

^{31}P NMR of imine **1d** ($CDCl_3$, 162 Hz)

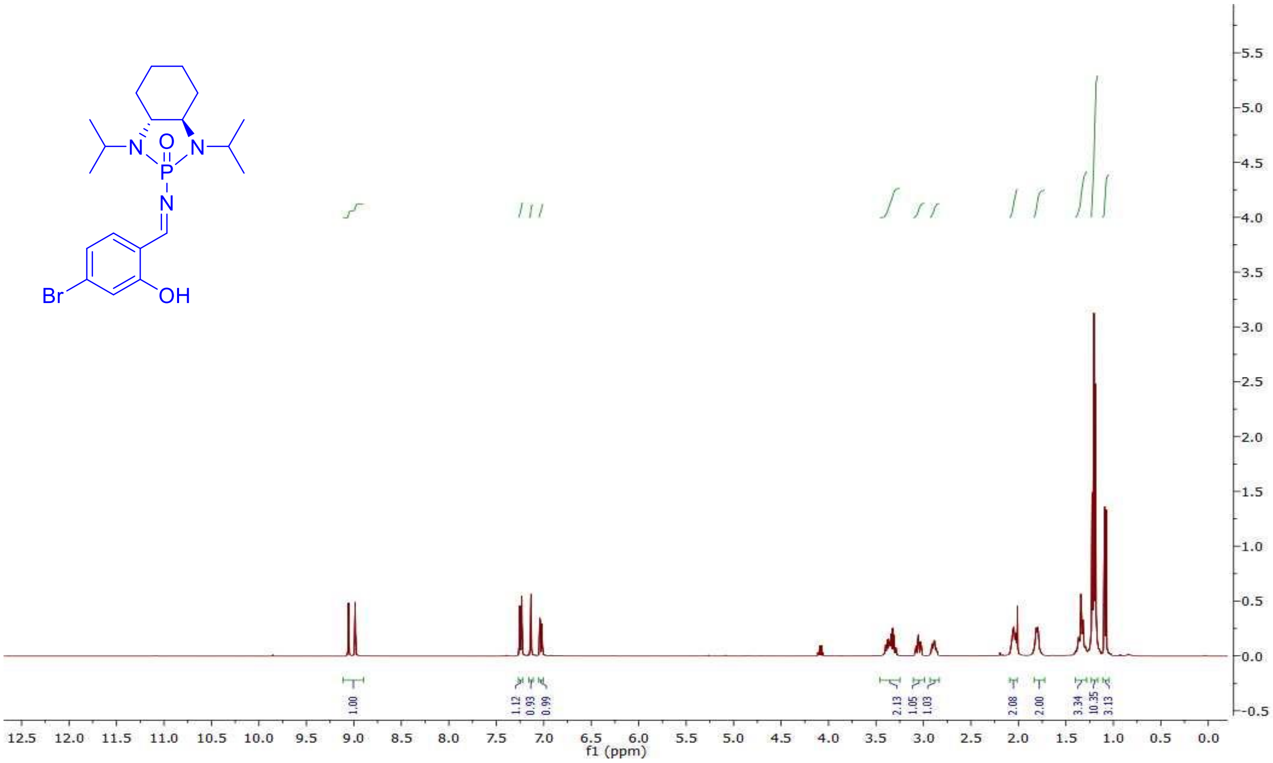

^{1}H NMR of imine **1e** (CDCl₃, 400 Hz)

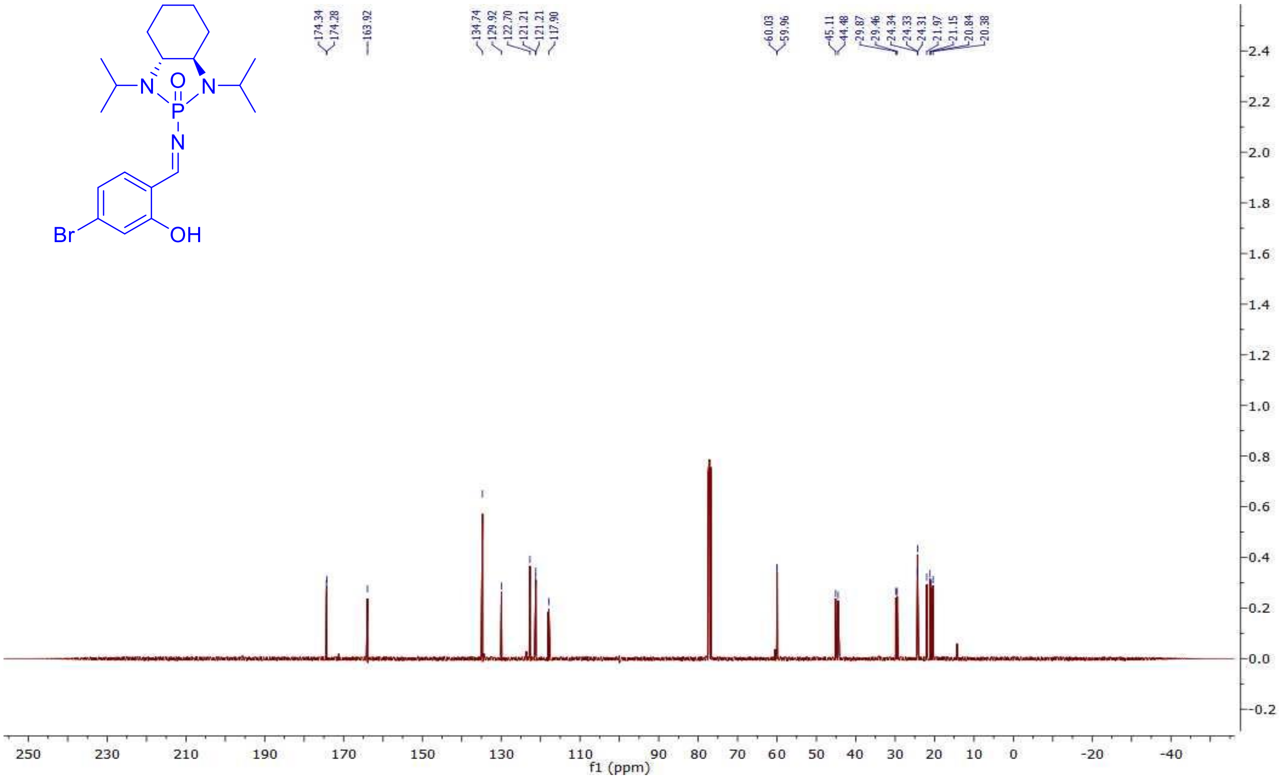

^{13}C NMR of imine **1e** (CDCl₃, 100 Hz)

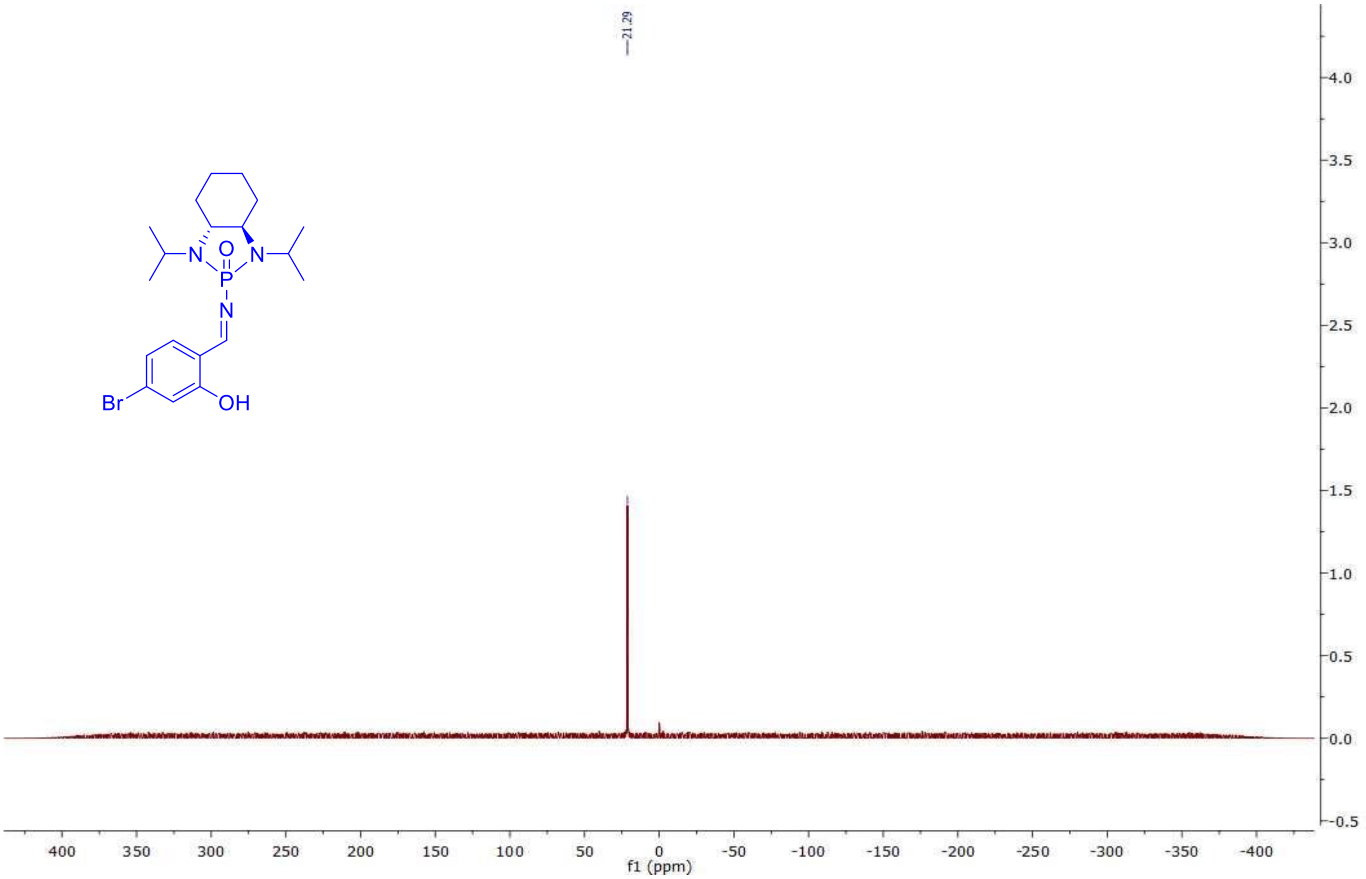

^{31}P NMR of imine **1e** ($CDCl_3$, 162 Hz)

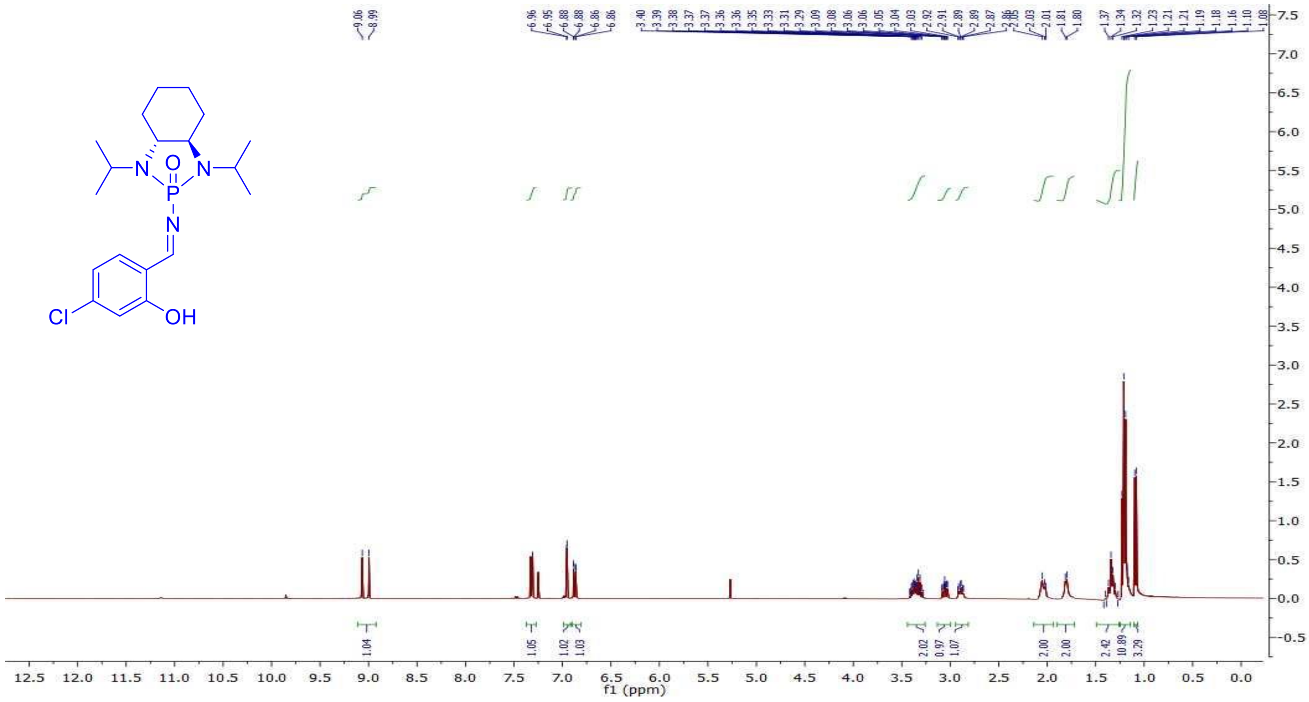

^{1}H NMR of imine **1f** ($CDCl_3$, 400 Hz)

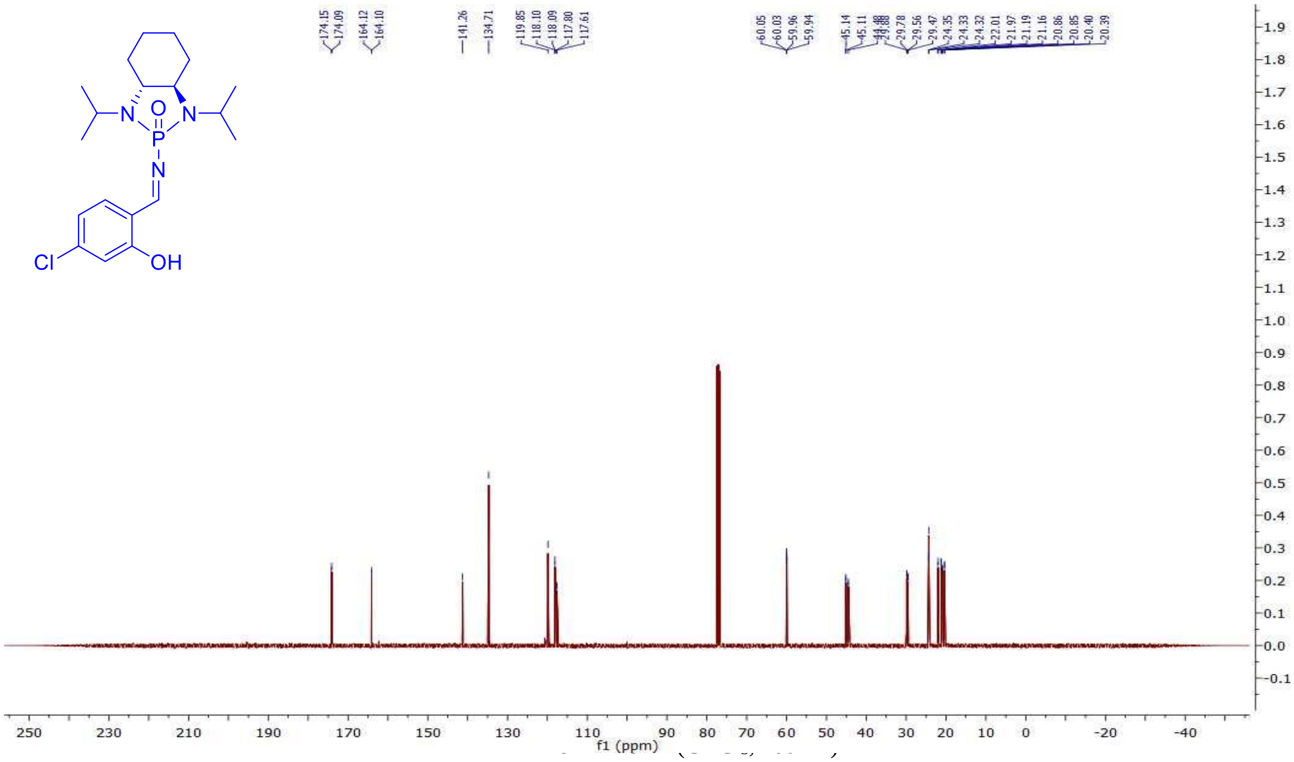

^{13}C NMR of imine **1f** ($CDCl_3$, 100 Hz)

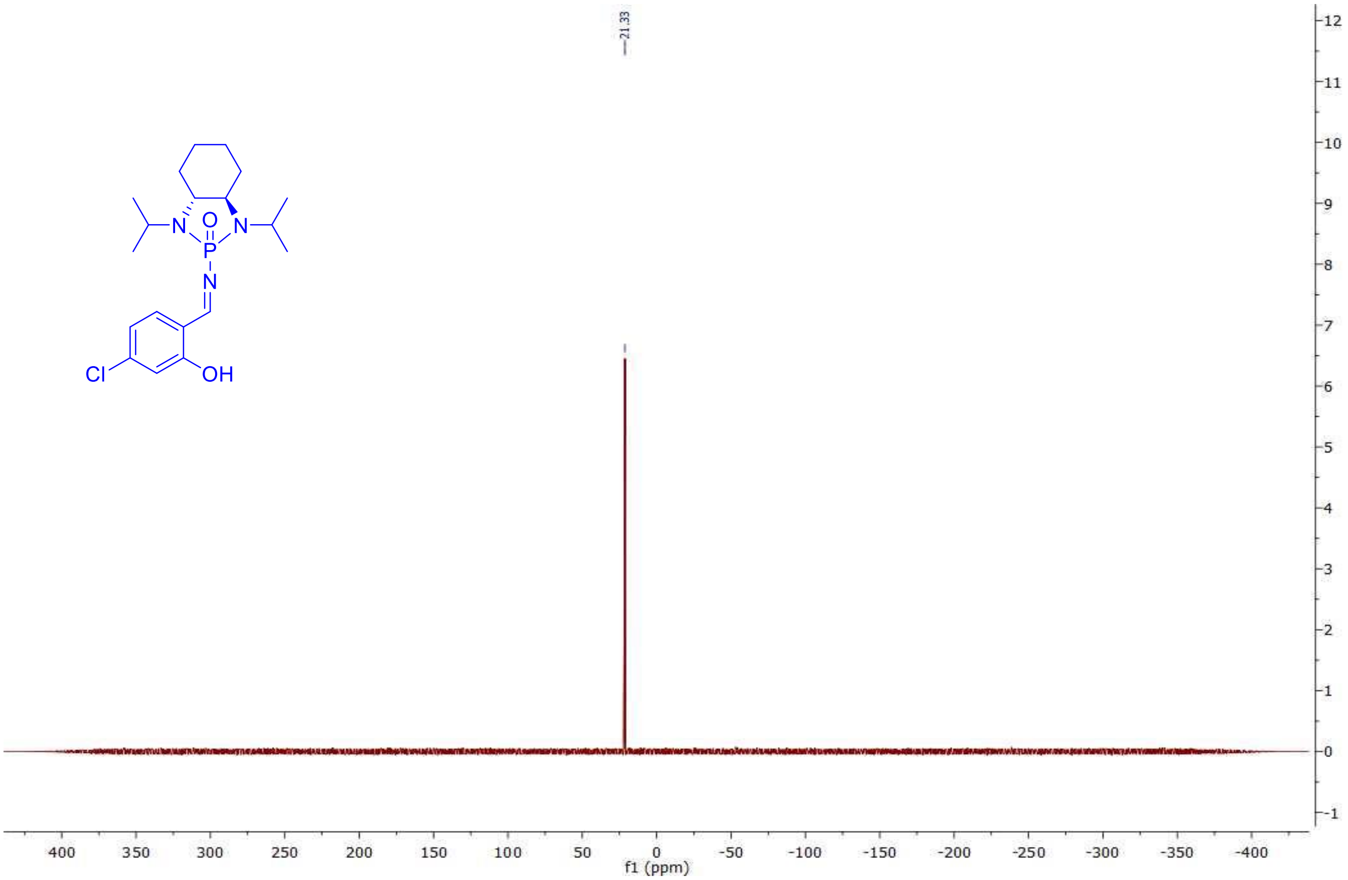

^{31}P NMR of imine **1f** ($CDCl_3$, 162 Hz)

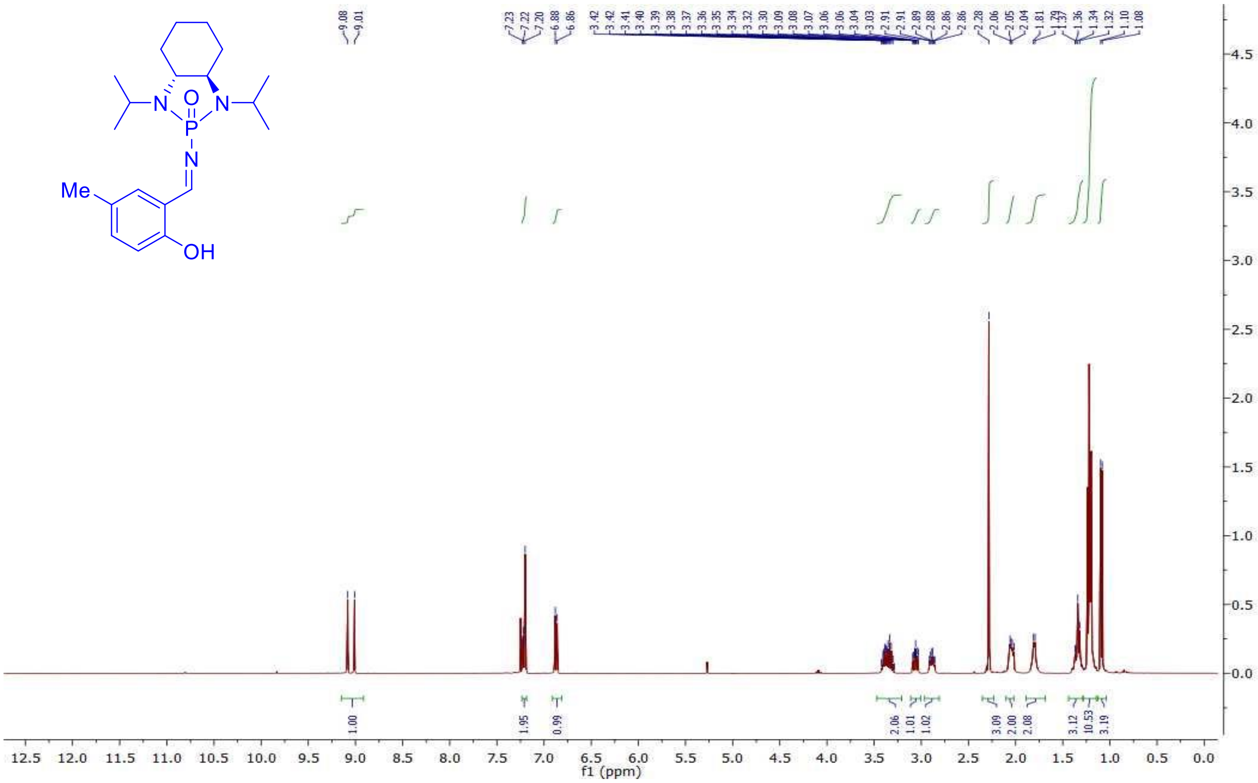

^{1}H NMR of imine **1g** (CDCl$_3$, 400 Hz)

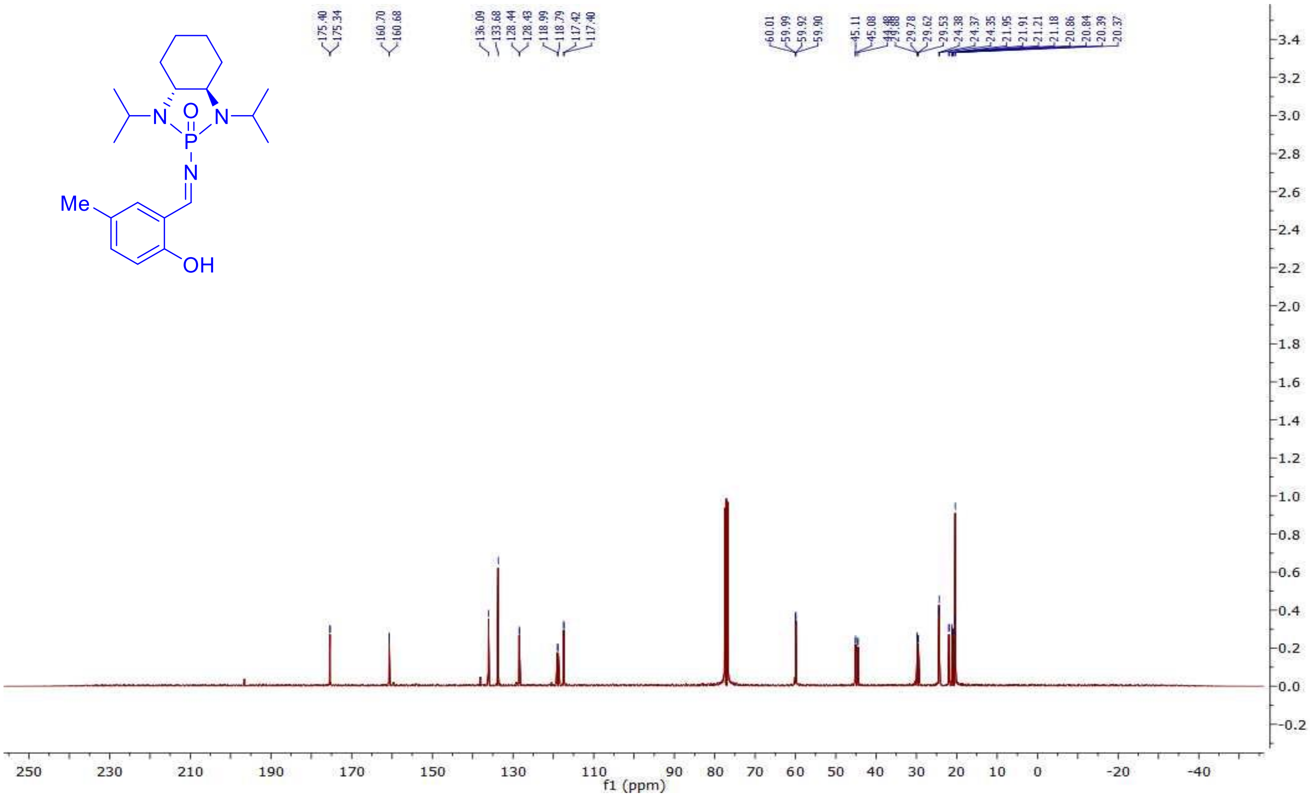

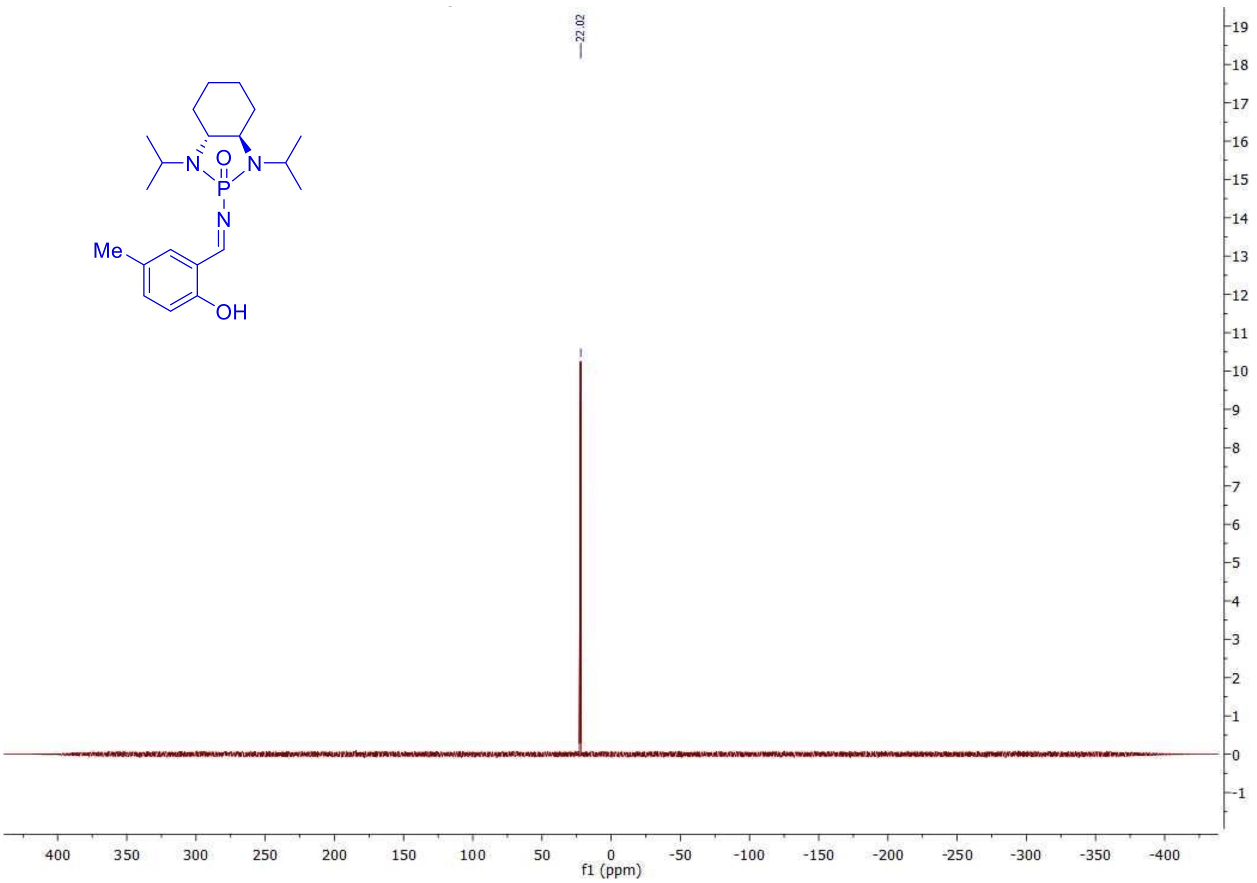

^{31}P NMR of imine **1g** ($CDCl_3$, 162 Hz)

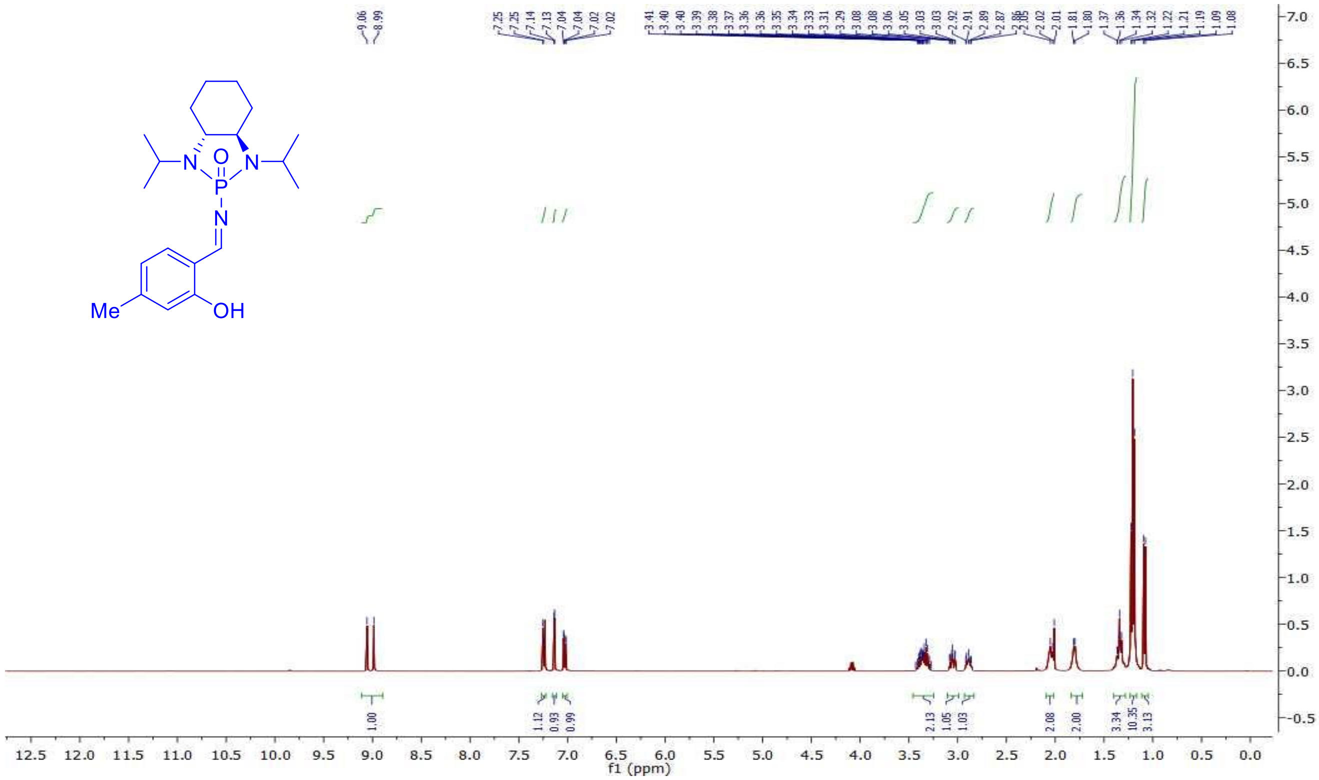

^{1}H NMR of imine **1h** ($CDCl_3$, 400 Hz)

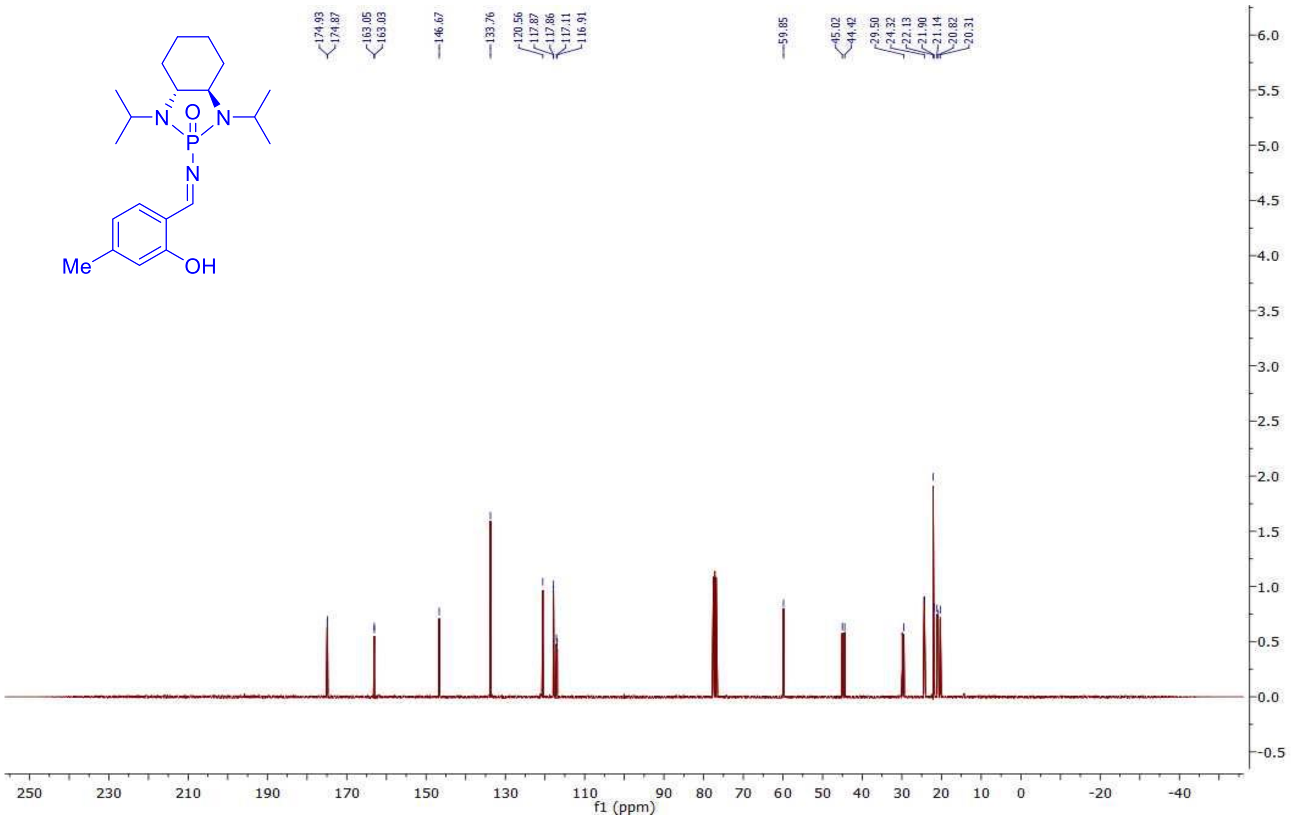

^{13}C NMR of imine **1h** ($CDCl_3$, 100 Hz)

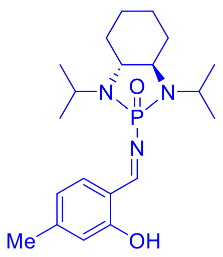

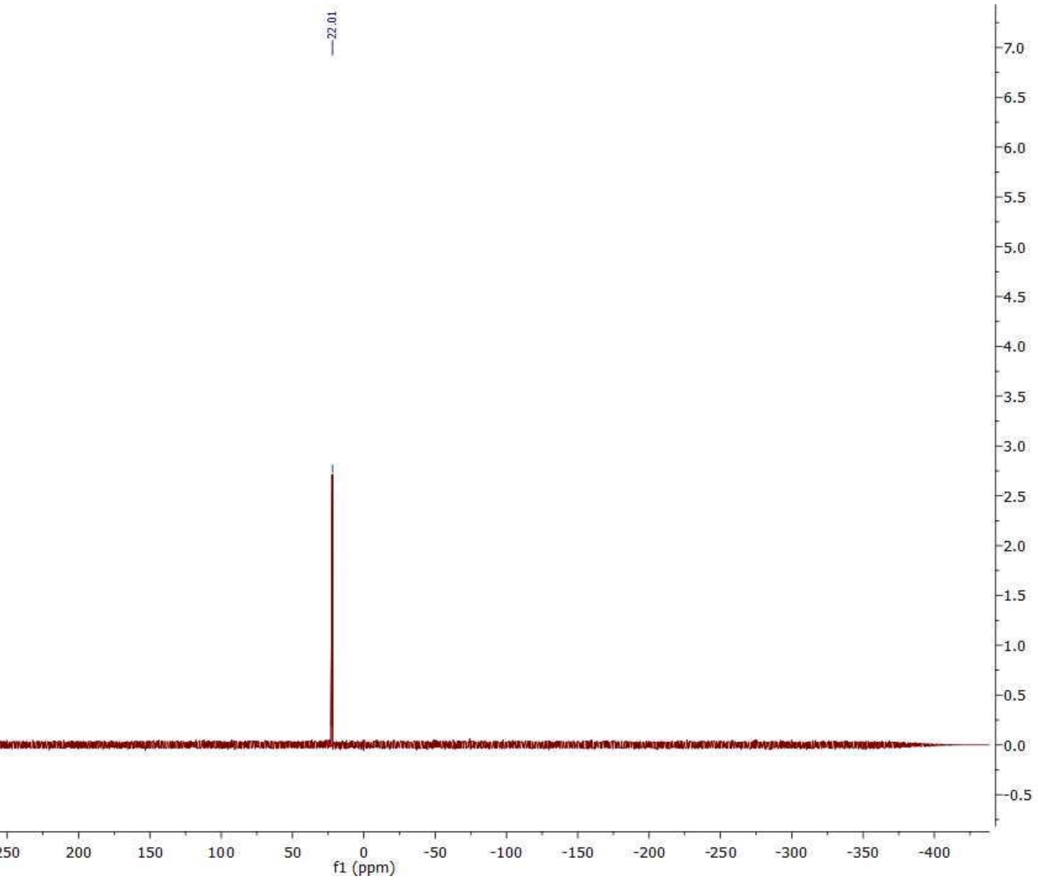

^{31}P NMR of imine **1h** ($CDCl_3$, 162 Hz)

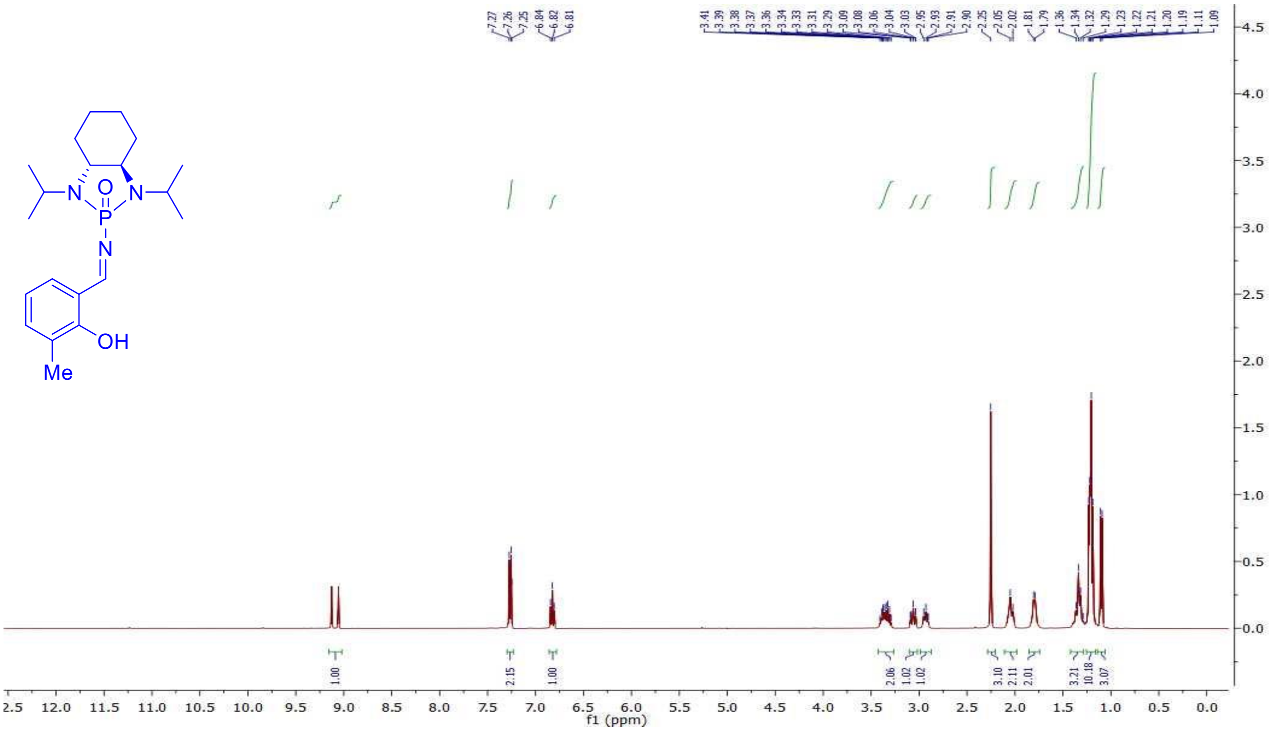

^{1}H NMR of imine **1i** ($CDCl_3$, 400 Hz)

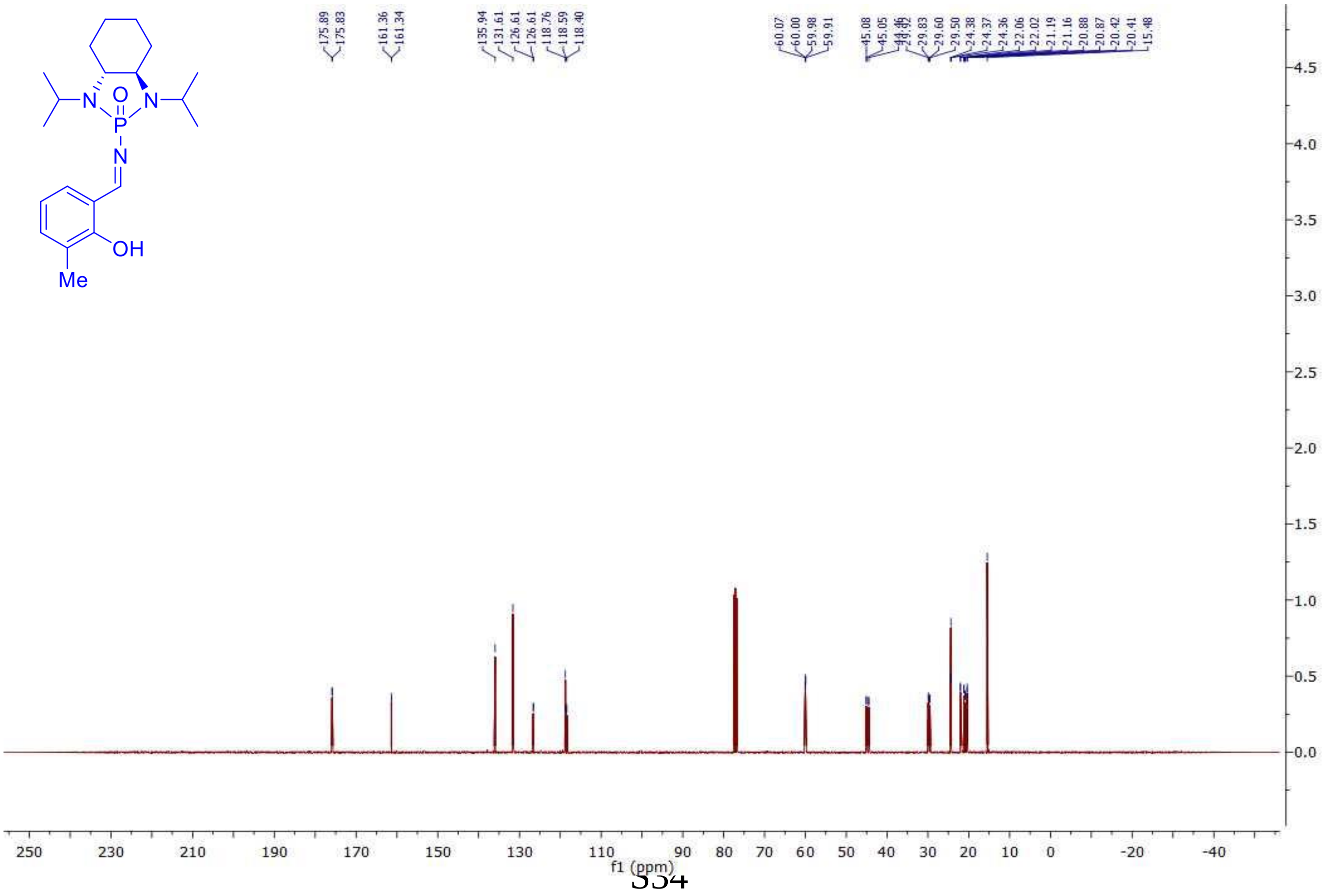

^{13}C NMR of imine **1i** ($CDCl_3$, 100 Hz)

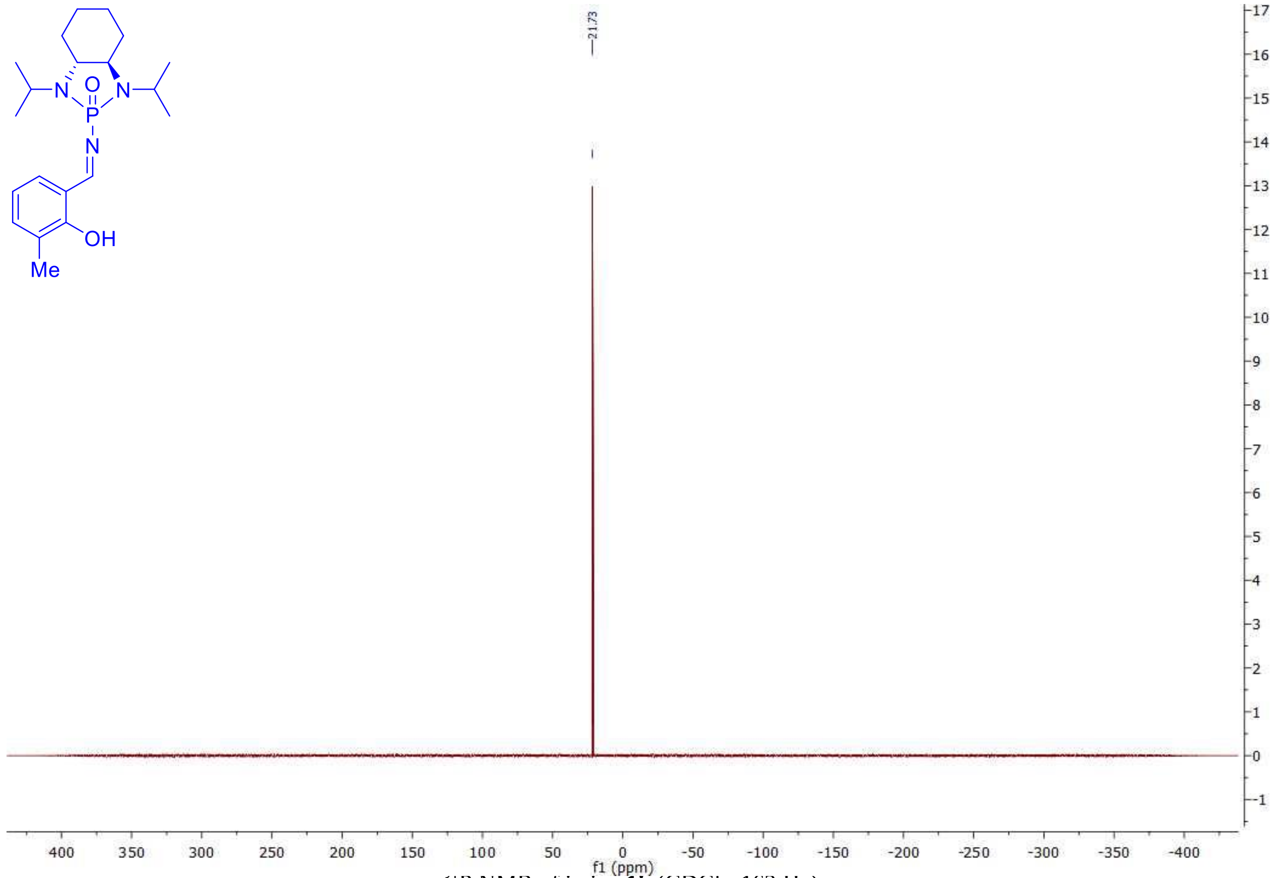

^{31}P NMR of imine **1i** ($CDCl_3$, 162 Hz)

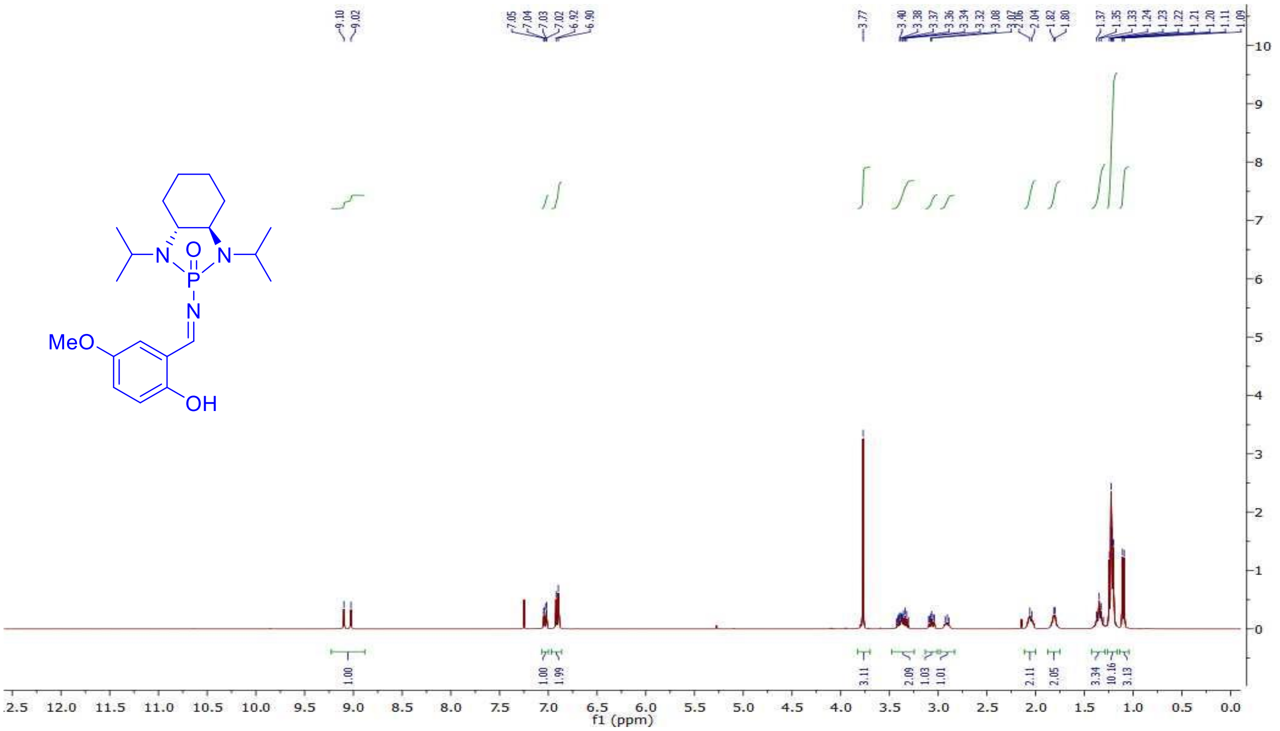

^{1}H NMR of imine **1j** ($CDCl_3$, 400 Hz)

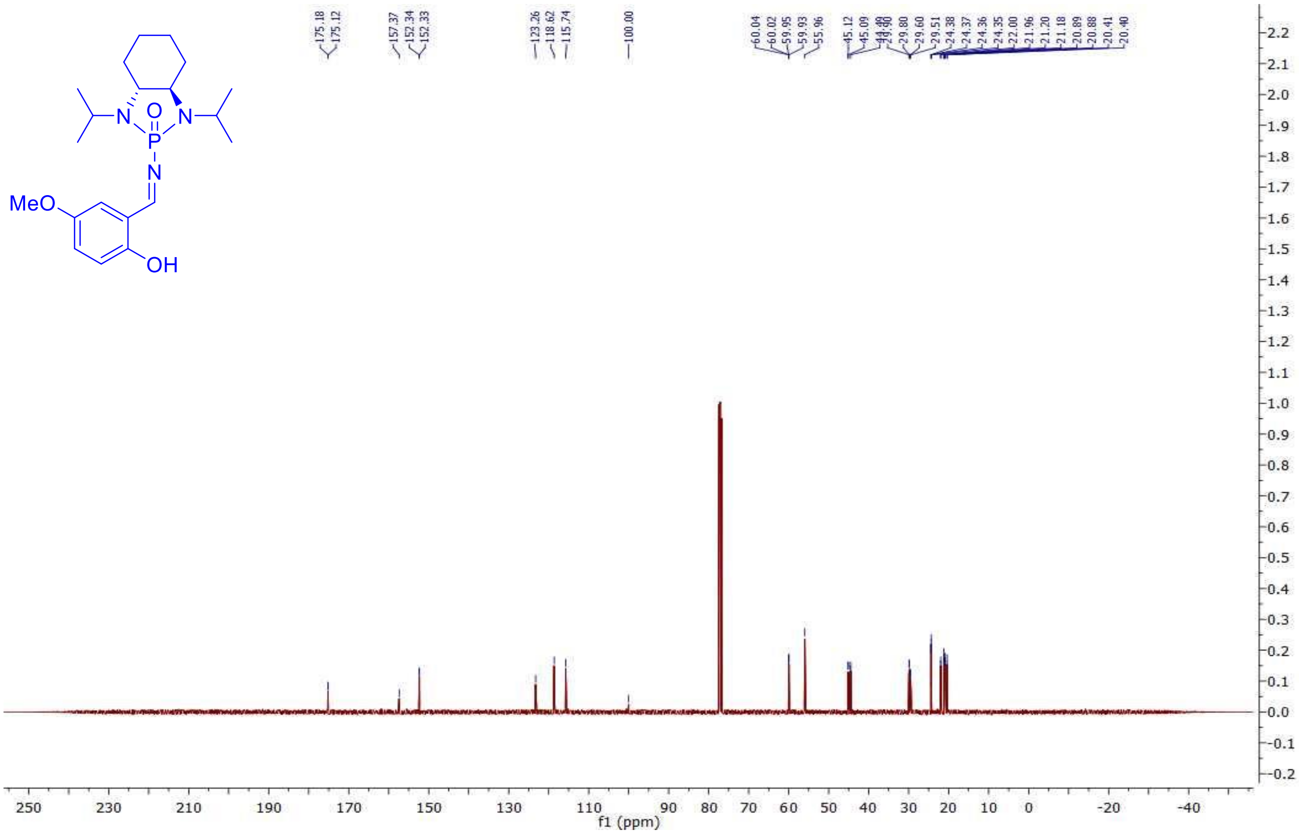

[3]C NMR of imine **1j** ($CDCl_3$, 100 Hz)

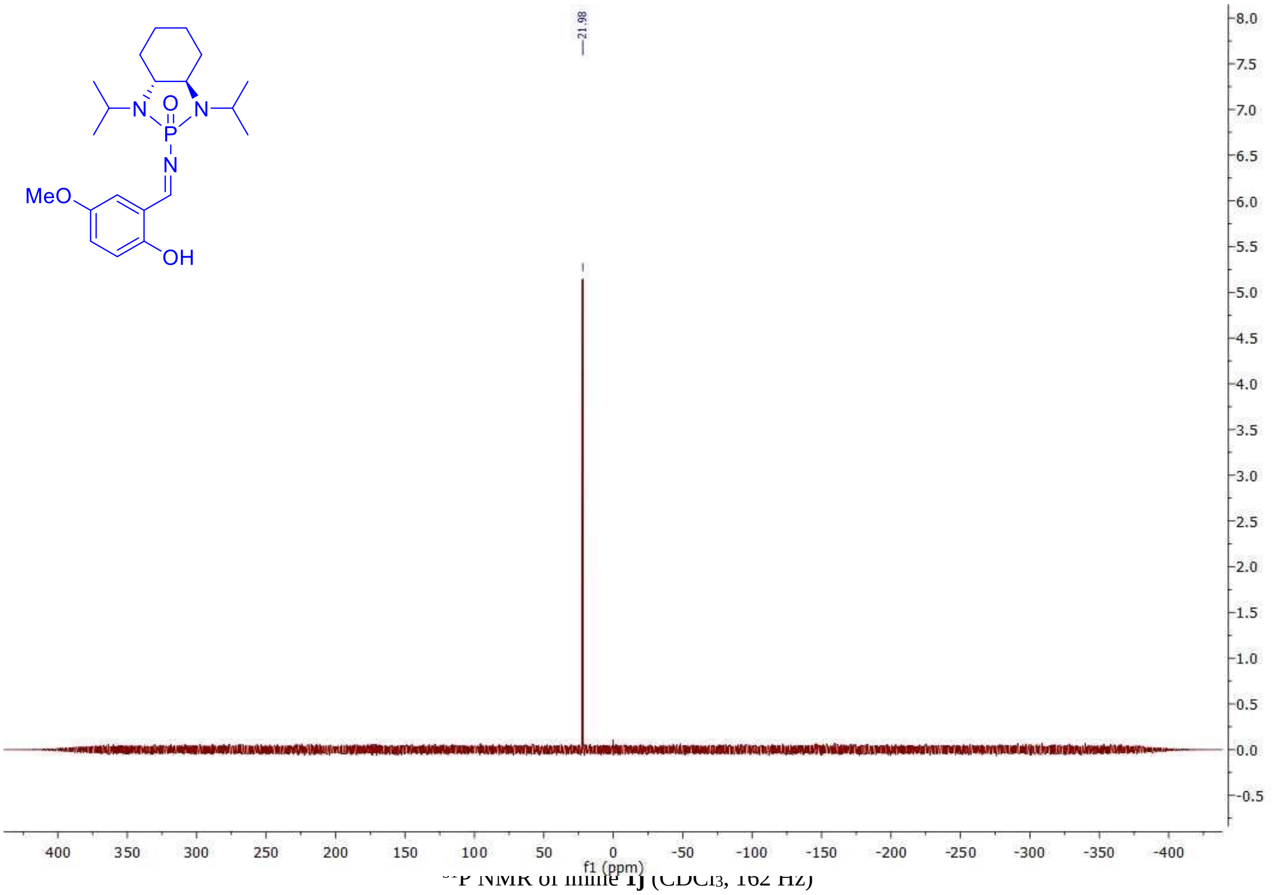

^{31}P NMR of imine **1j** ($CDCl_3$, 162 Hz)

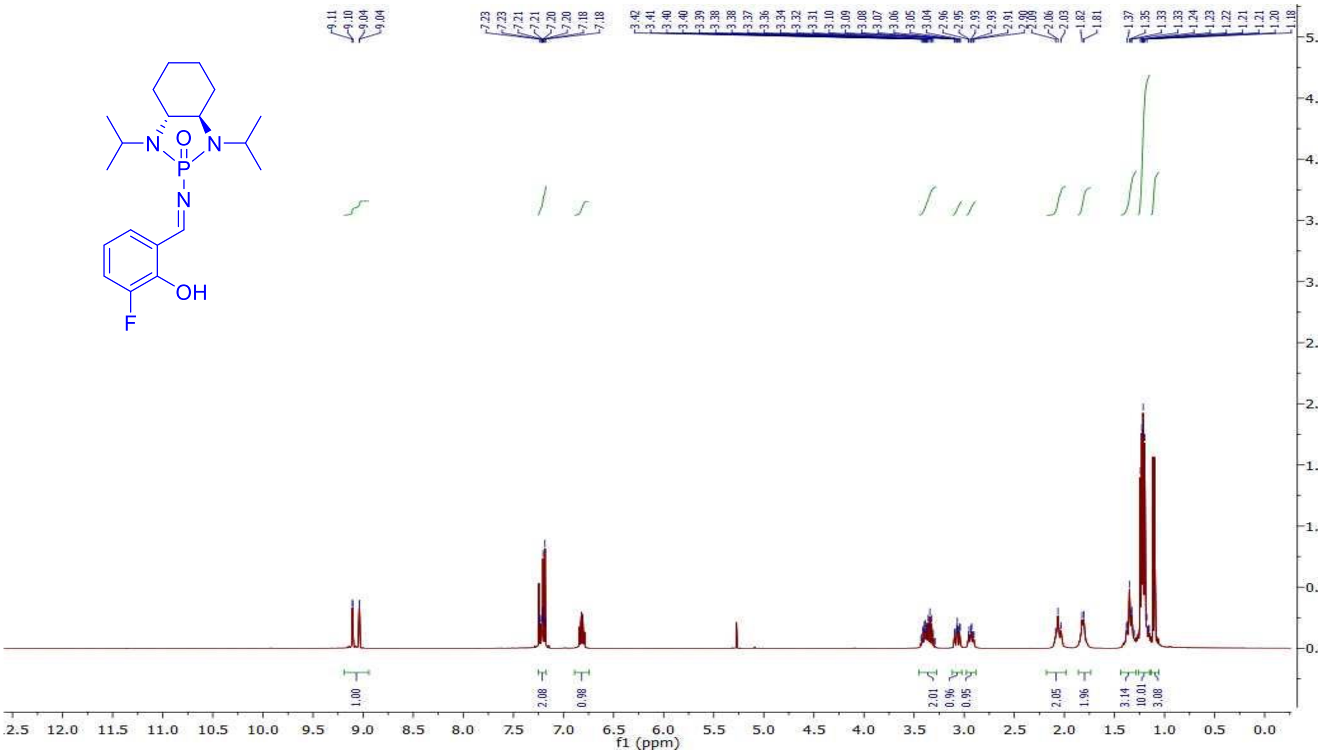

^{1}H NMR of imine **1k** ($CDCl_3$, 400 Hz)

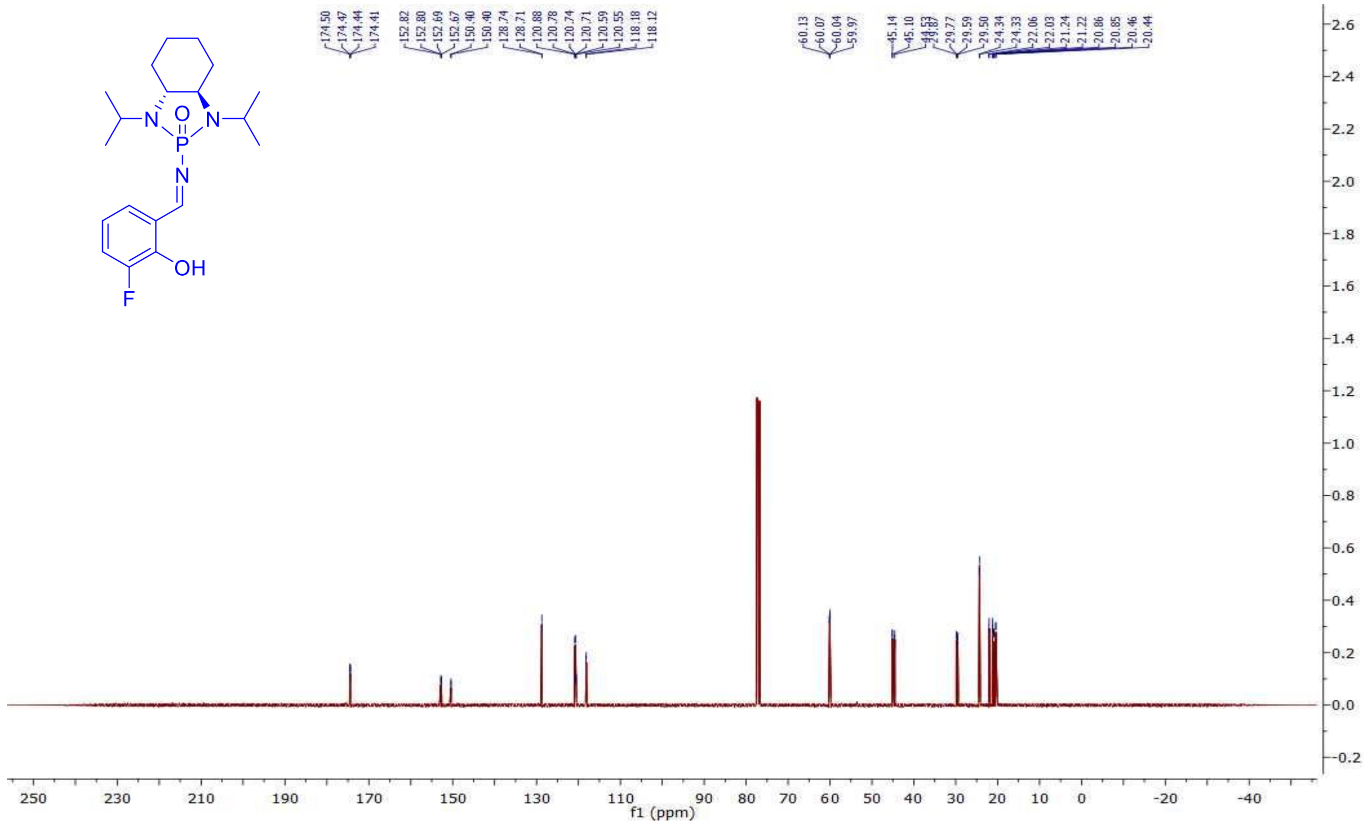

^{13}C NMR of imine **1k** ($CDCl_3$, 100 Hz)

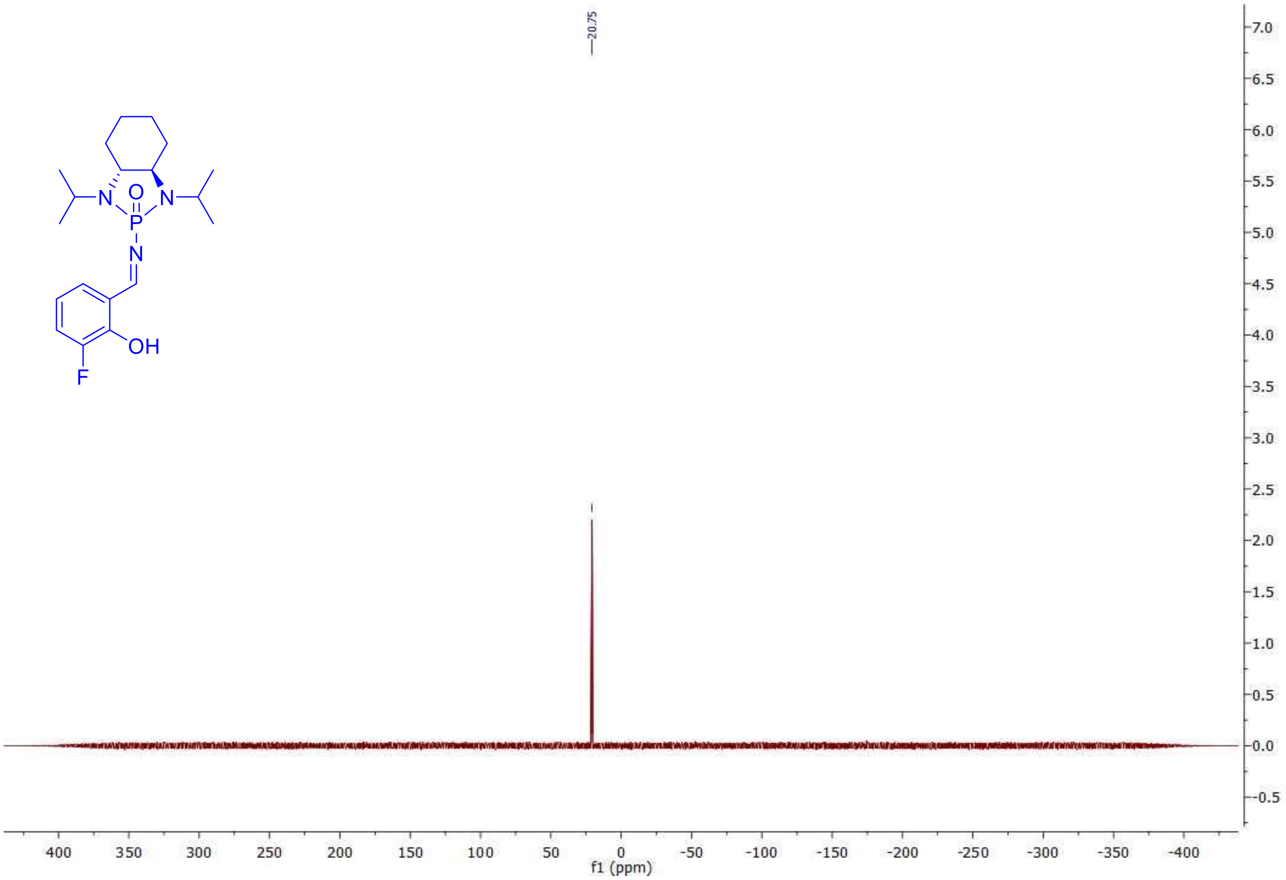

^{31}P NMR of imine **1k** ($CDCl_3$, 162 Hz)

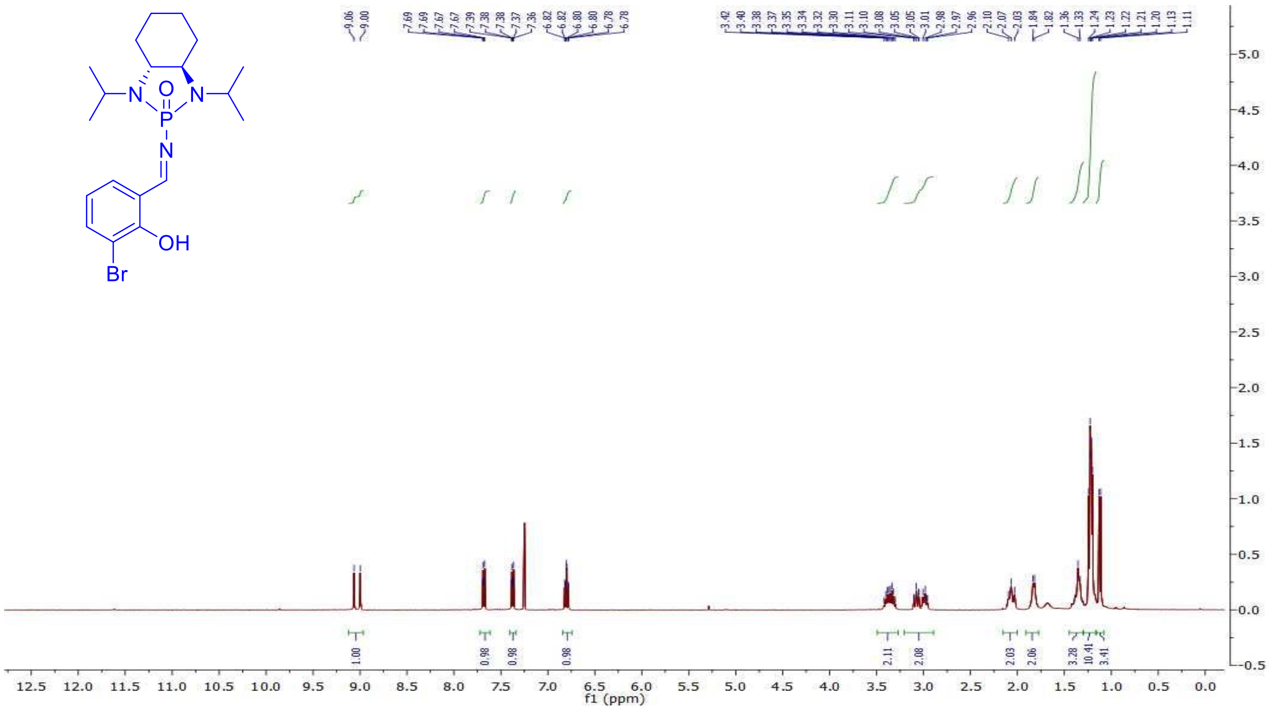

^{1}H NMR of imine **1l** ($CDCl_3$, 400 Hz)

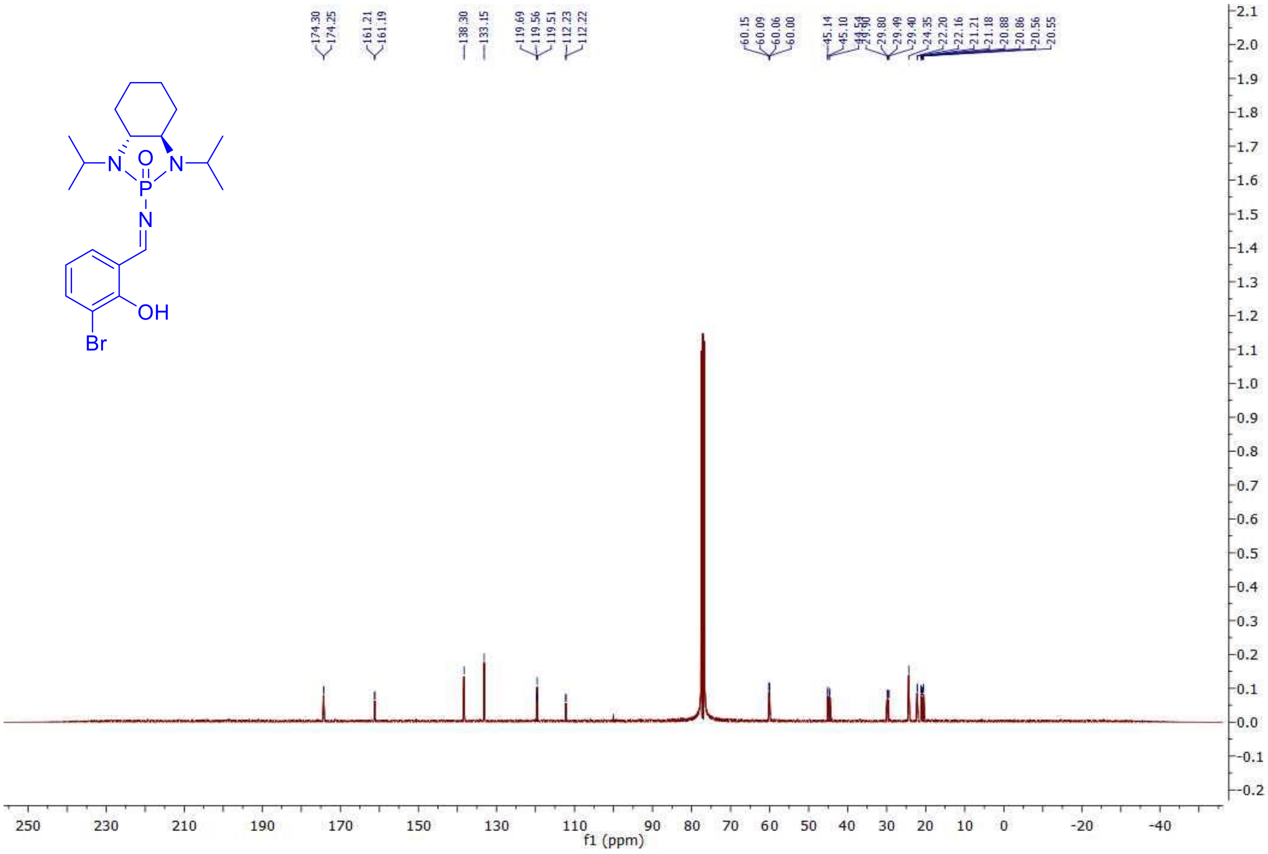

^{13}C NMR of imine **1l** ($CDCl_3$, 100 Hz)

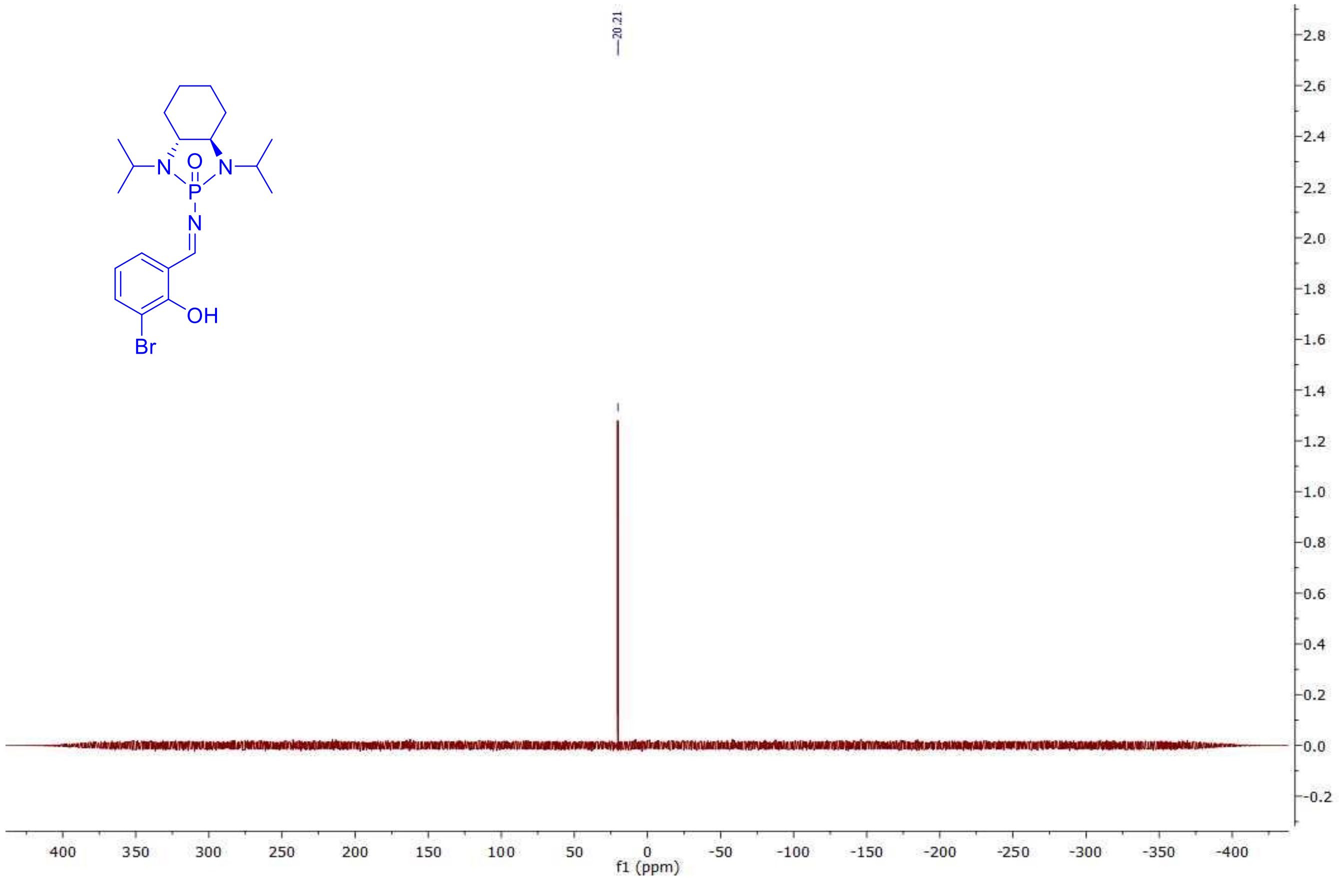

^{31}P NMR of imine **1l** ($CDCl_3$, 162 Hz)

Copies of ^{1}H, ^{13}C NMR and ^{31}P NMR, Spectra for Compounds 3a-3q

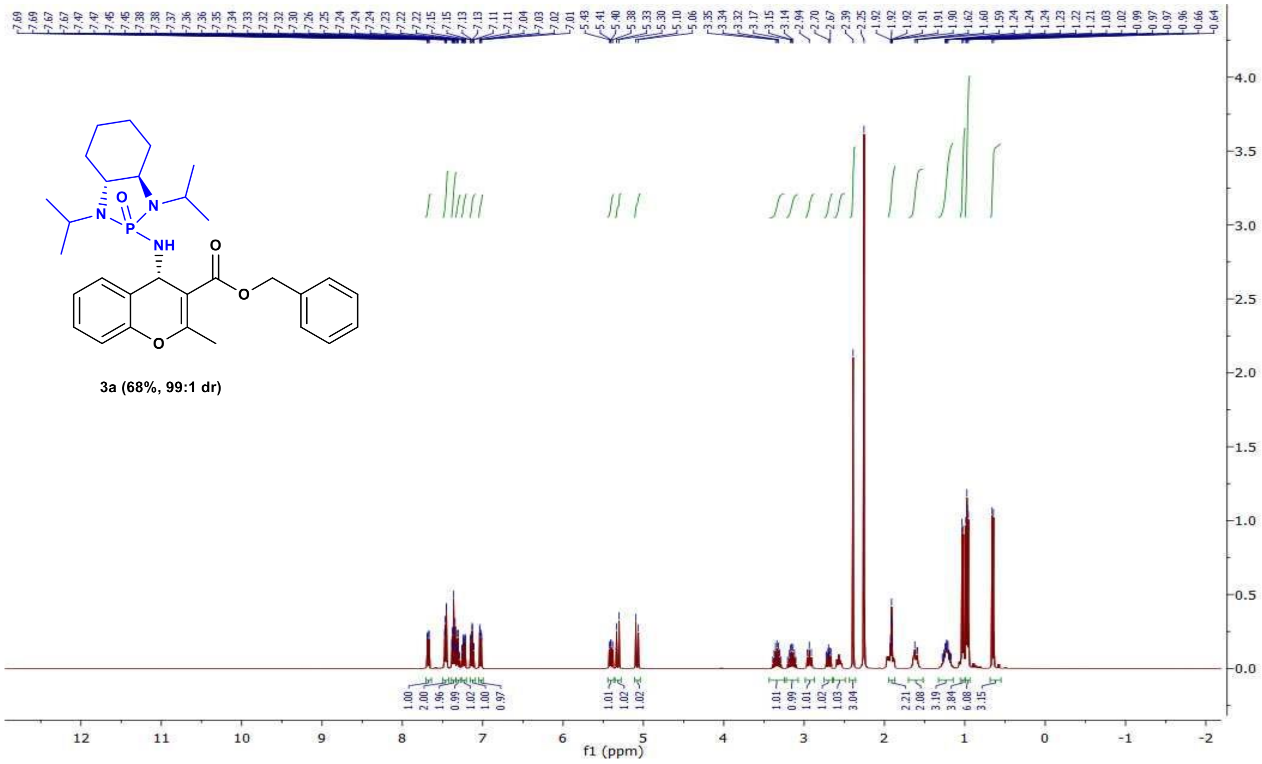

^{1}H NMR of Chromene **3a** (CD_3CN, 400 Hz)

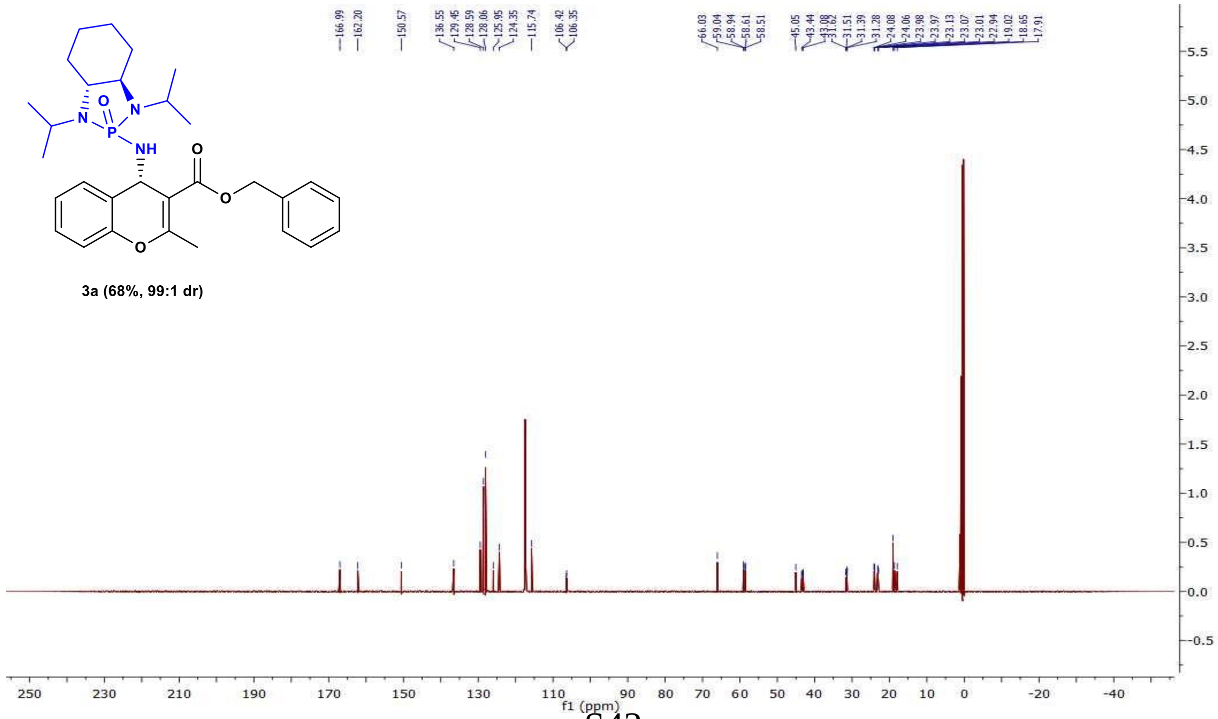

^{31}C NMR of Chromene **3a** (CD_3CN, 101 Hz)

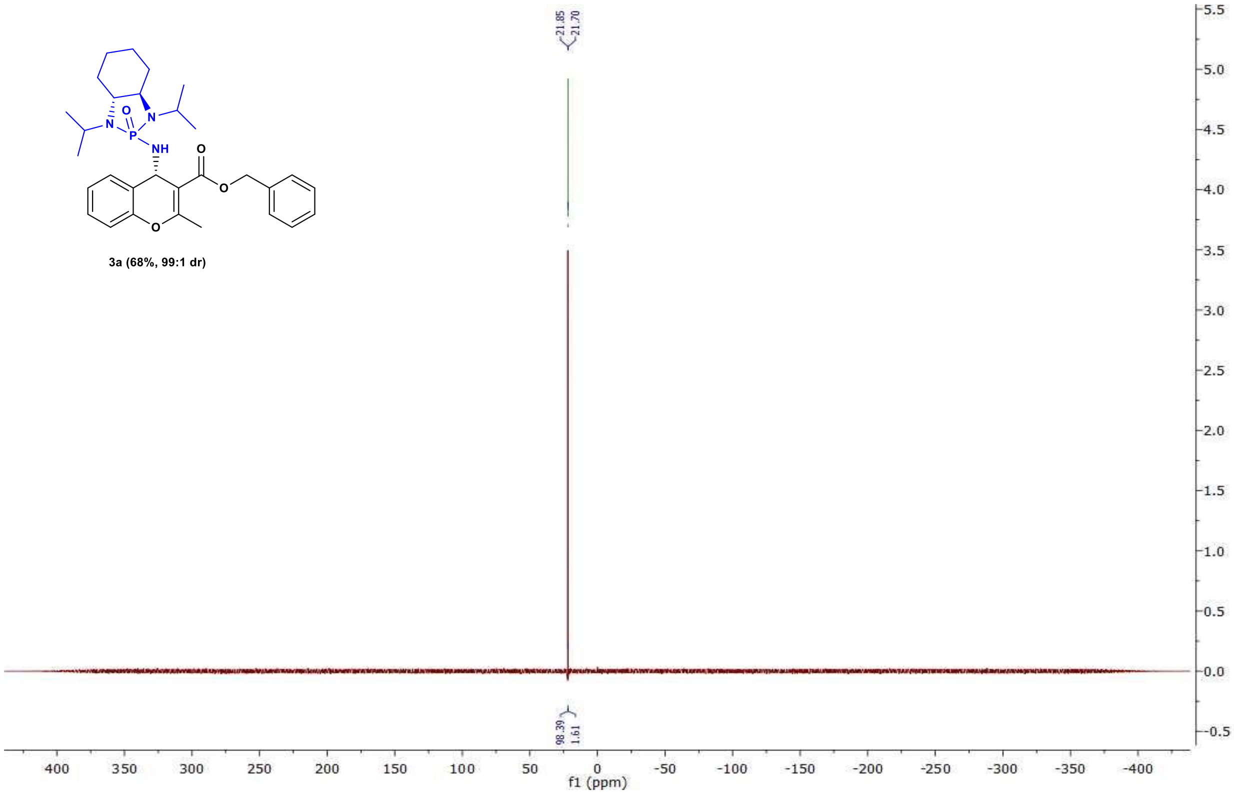

^{31}P NMR of Chromene **3a** (CD_3CN, 162 Hz)

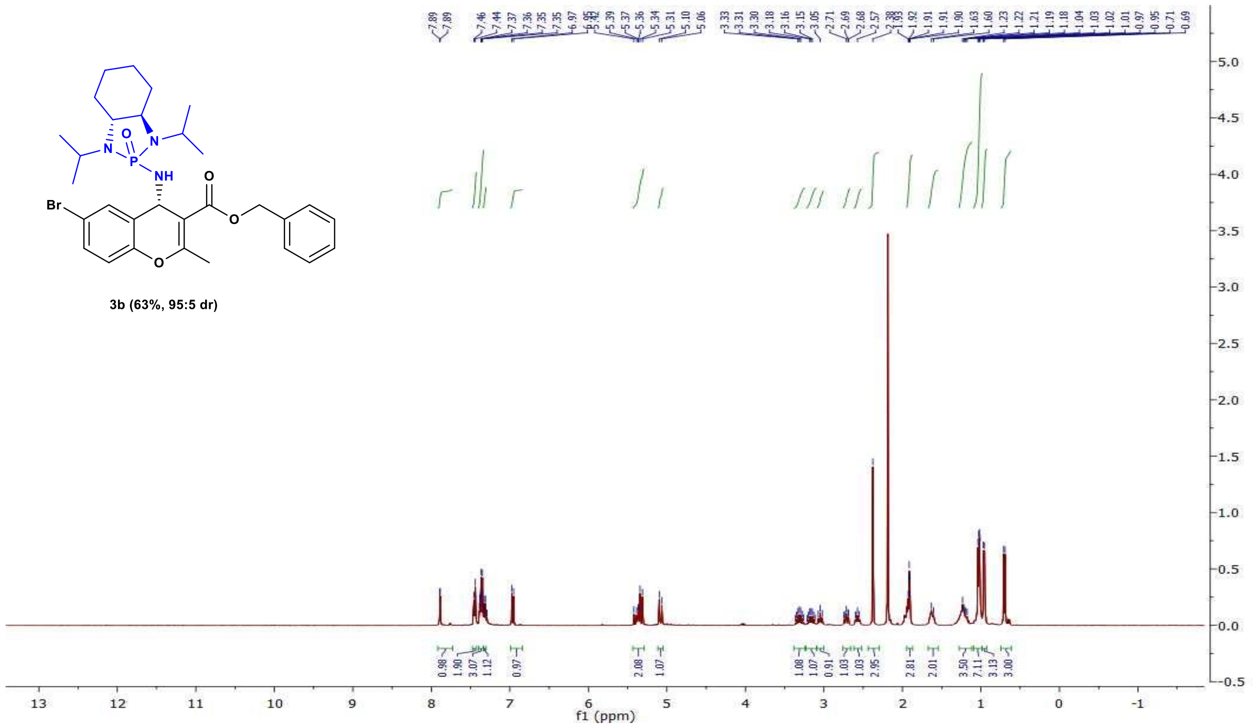

^{1}H NMR of Chromene **3b** (CD_3CN, 400 Hz)

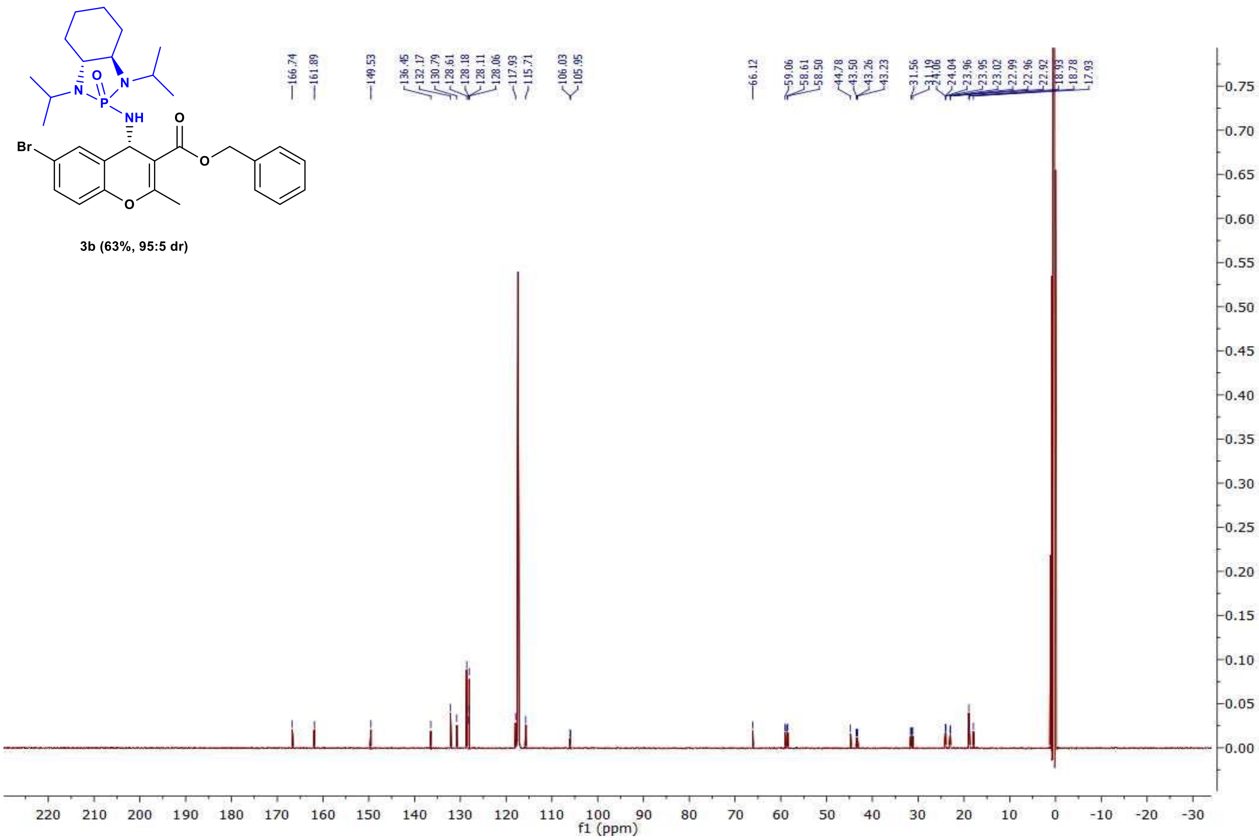

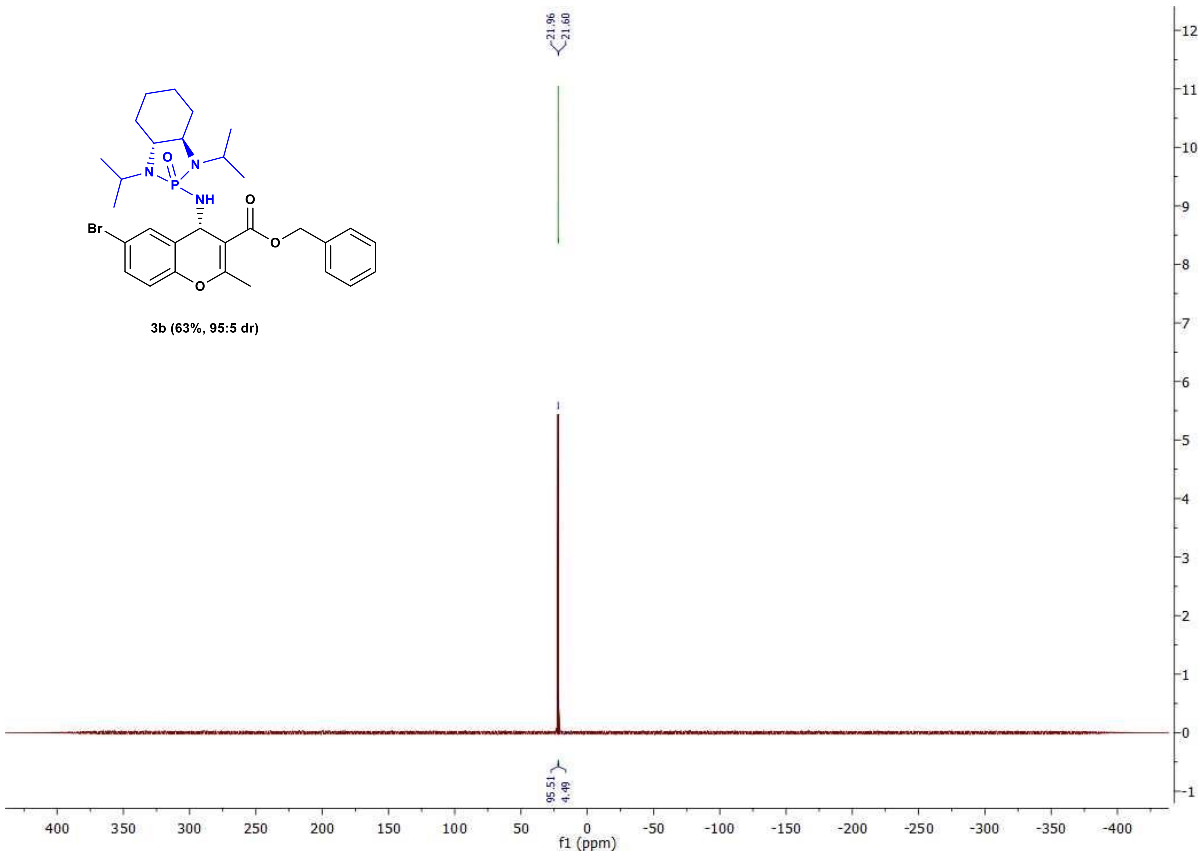
21.96
21.60
95.51
4.49
400
350
300
250
200
150
100
50
0
-50
-100
-150
-200
-250
-300
-350
-400
f1 (ppm)
12
11
10
9
8
7
6
5
4
3
2
1
0
-1

3b (63%, 95:5 dr)

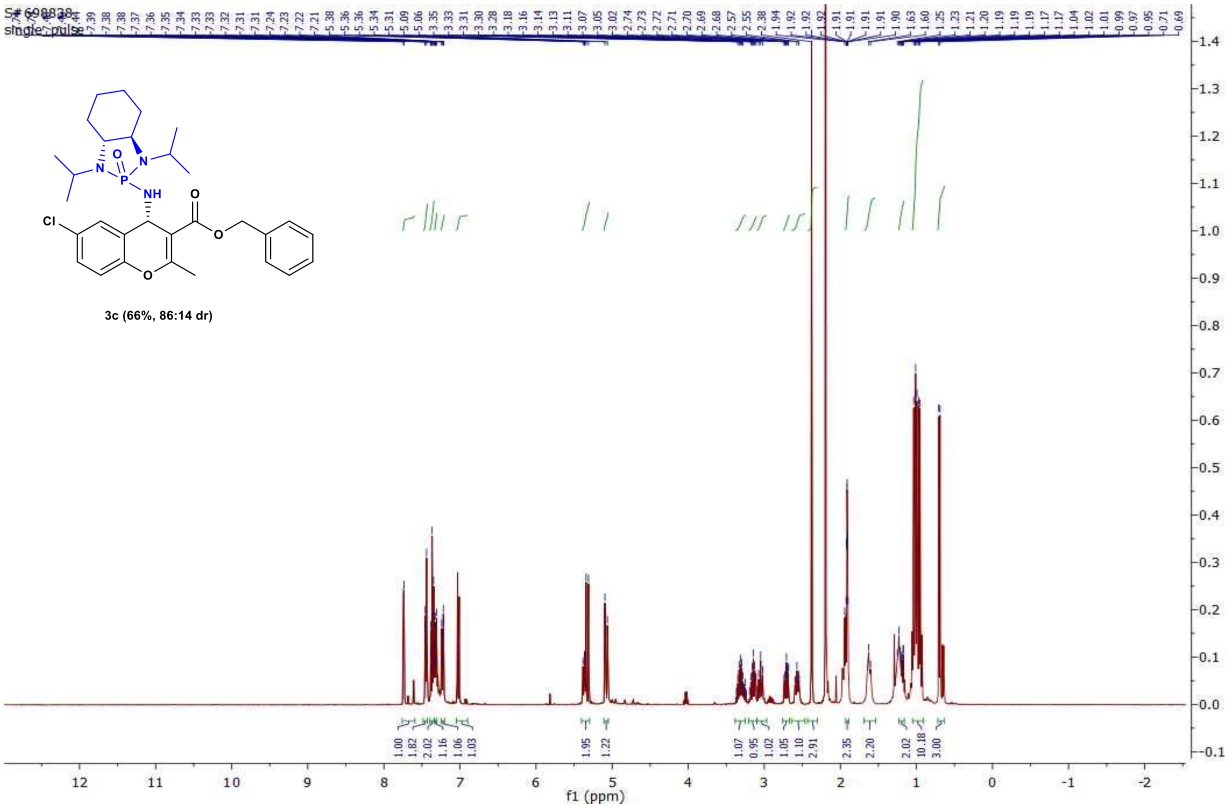

^{1}H NMR of Chromene **3c** (CD_3CN, 400 Hz)

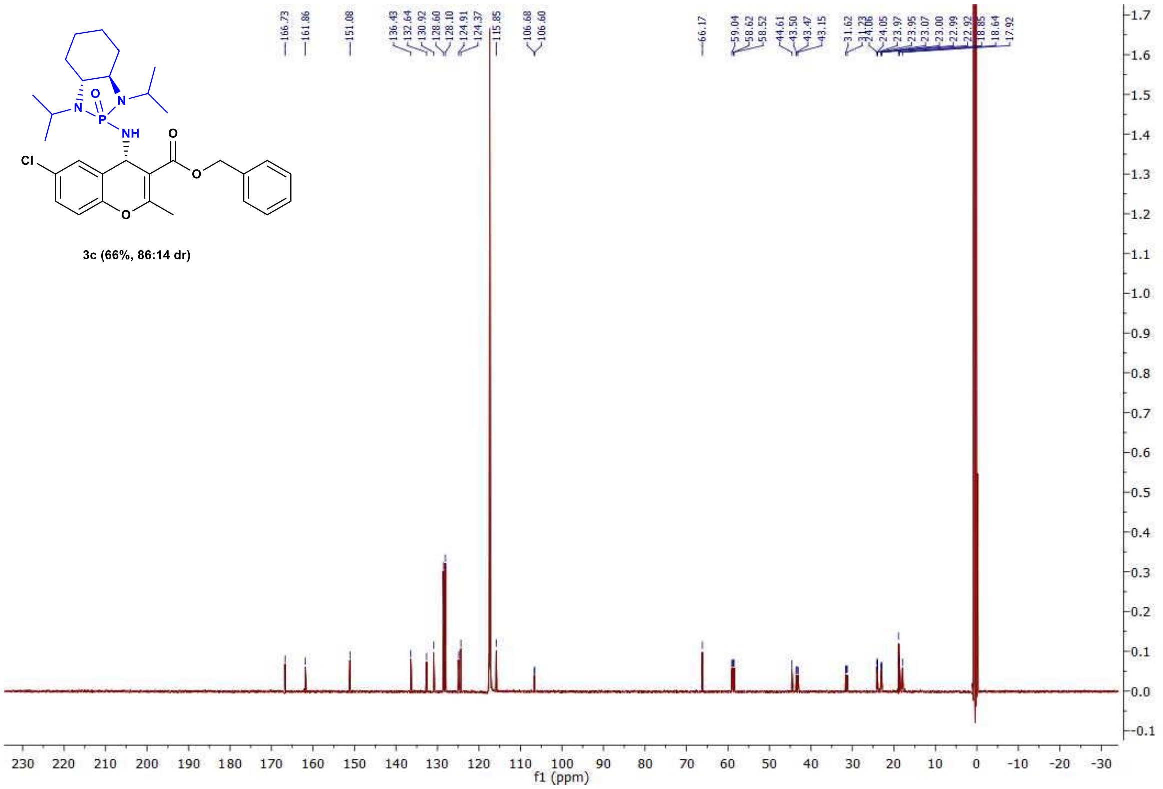

^{31}C NMR of Chromene **3c** (CD_3CN, 101 Hz)

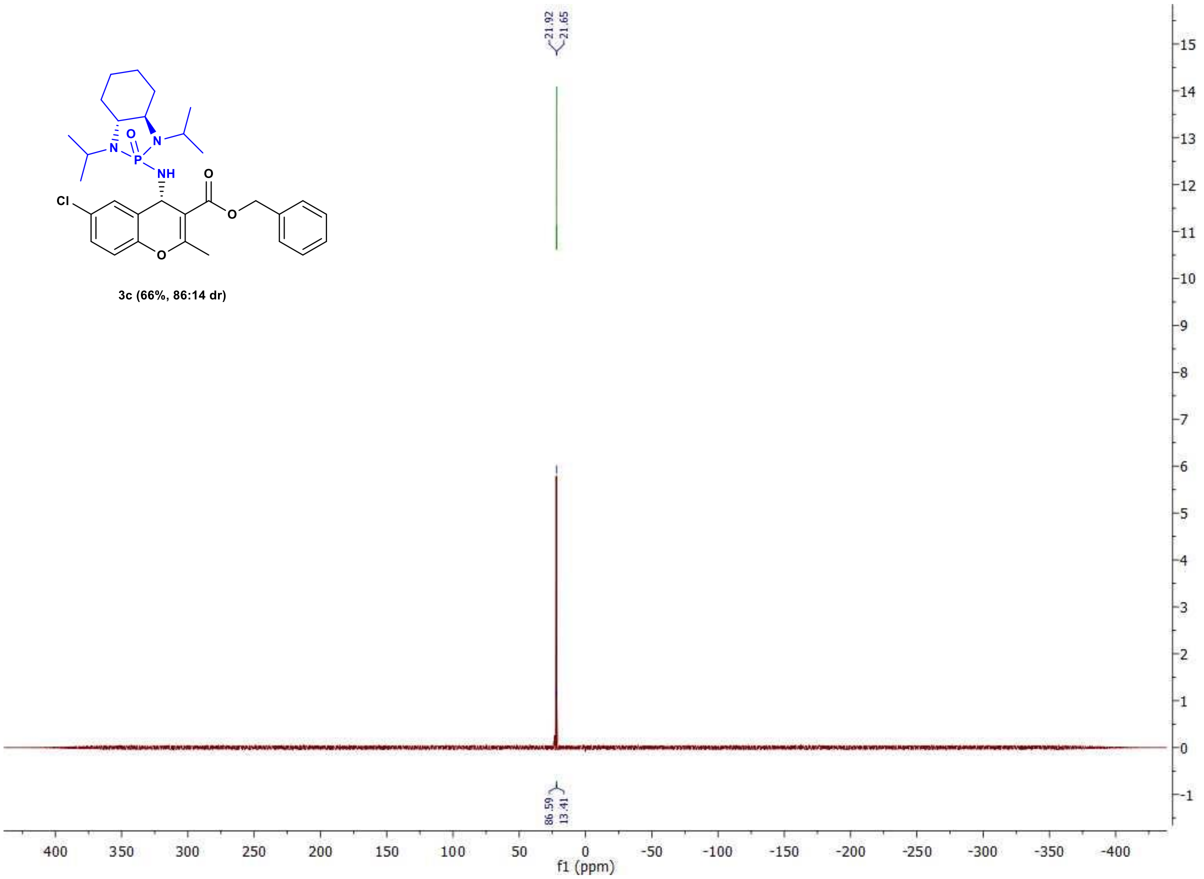

3c (66%, 86:14 dr)

^{31}P NMR of Chromene **3c** (CD_3CN, 162 Hz)

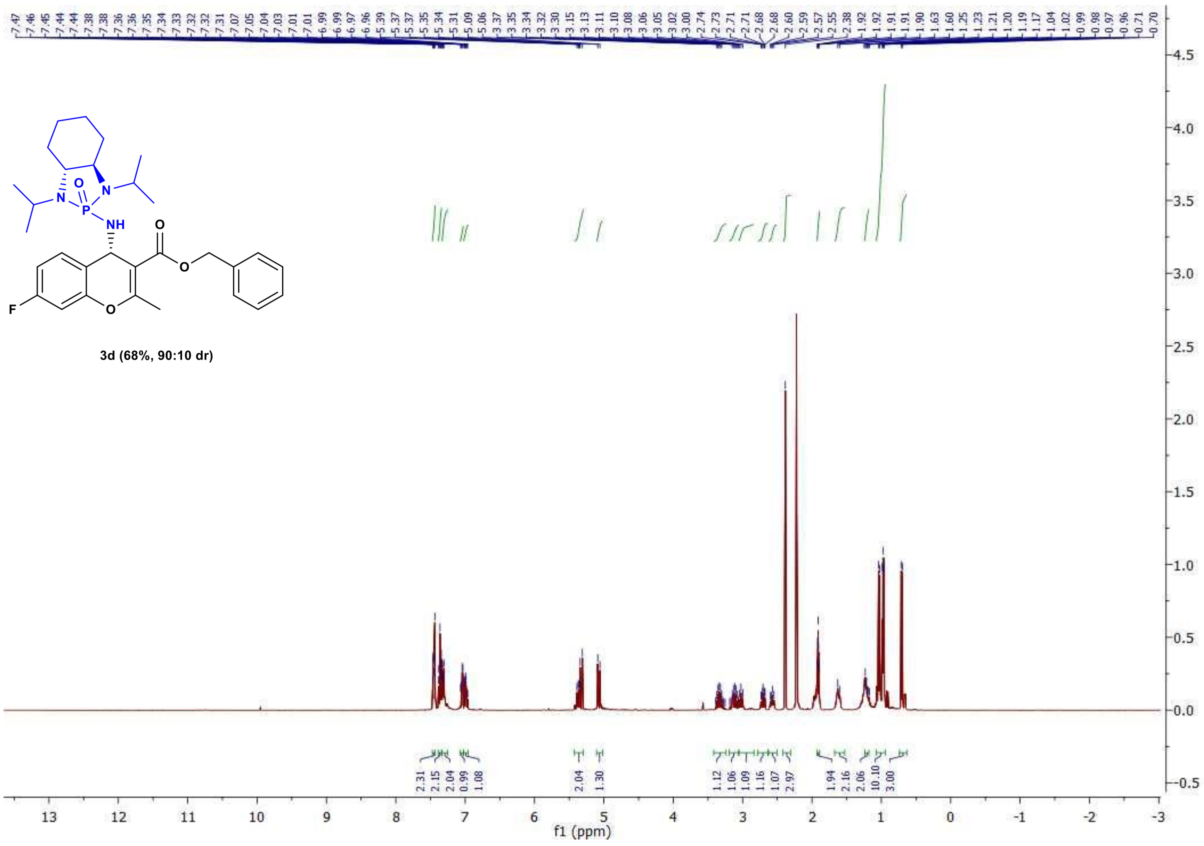

^{1}H NMR of Chromene **3d** (CD_3CN, 400 Hz)

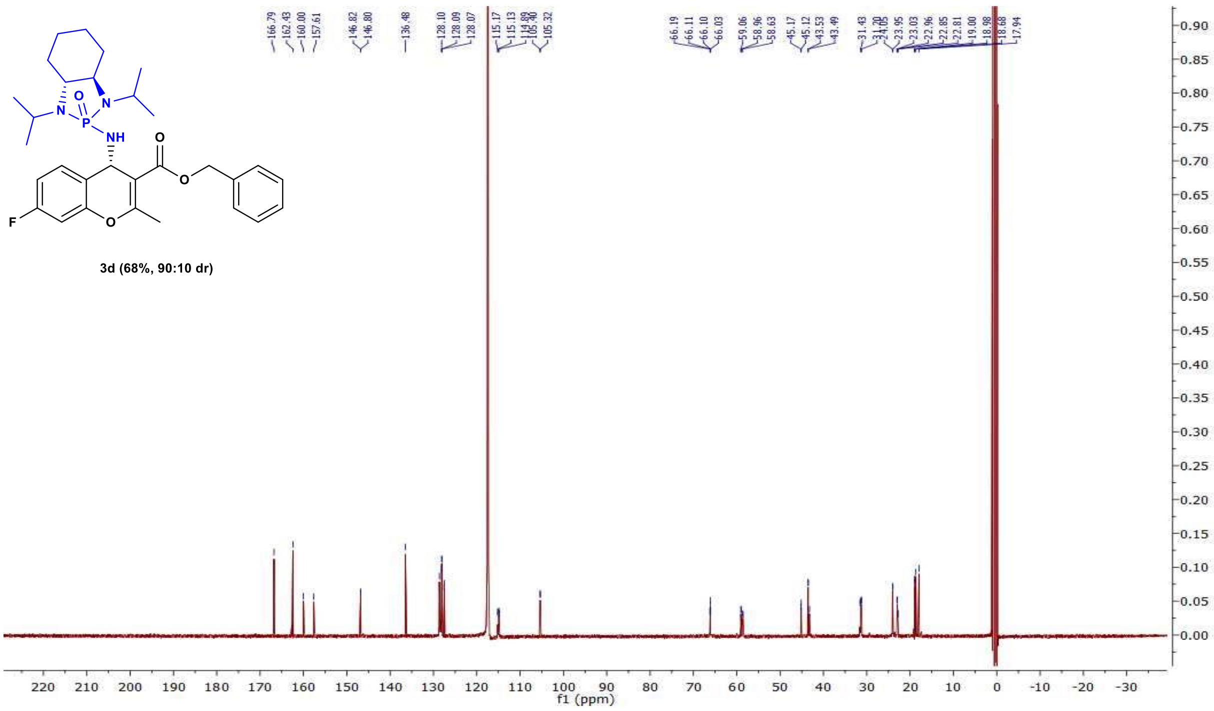

[31]C NMR of Chromene **3d** (CD_3CN, 101 Hz)

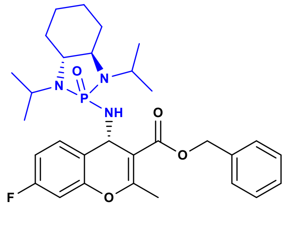
O
N
P
N
NH
O
F
O
O

3d (68%, 90:10 dr)

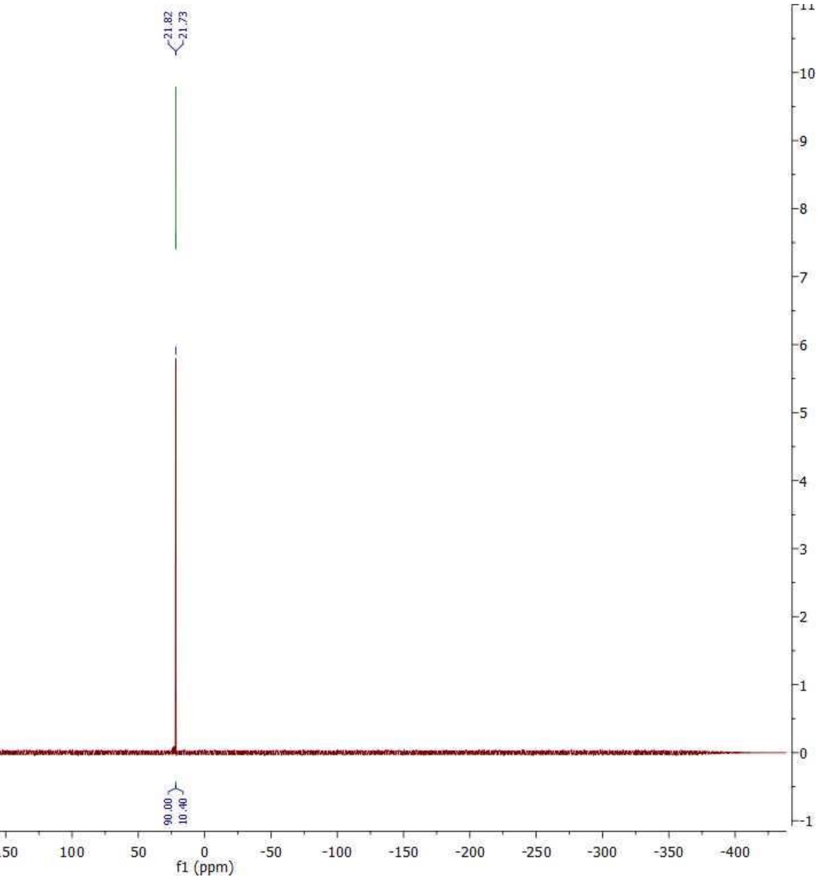
21.82
21.73
90.00
10.40
400
350
300
250
200
150
100
50
0
-50
-100
-150
-200
-250
-300
-350
-400
f1 (ppm)
11
10
9
8
7
6
5
4
3
2
1
0
-1

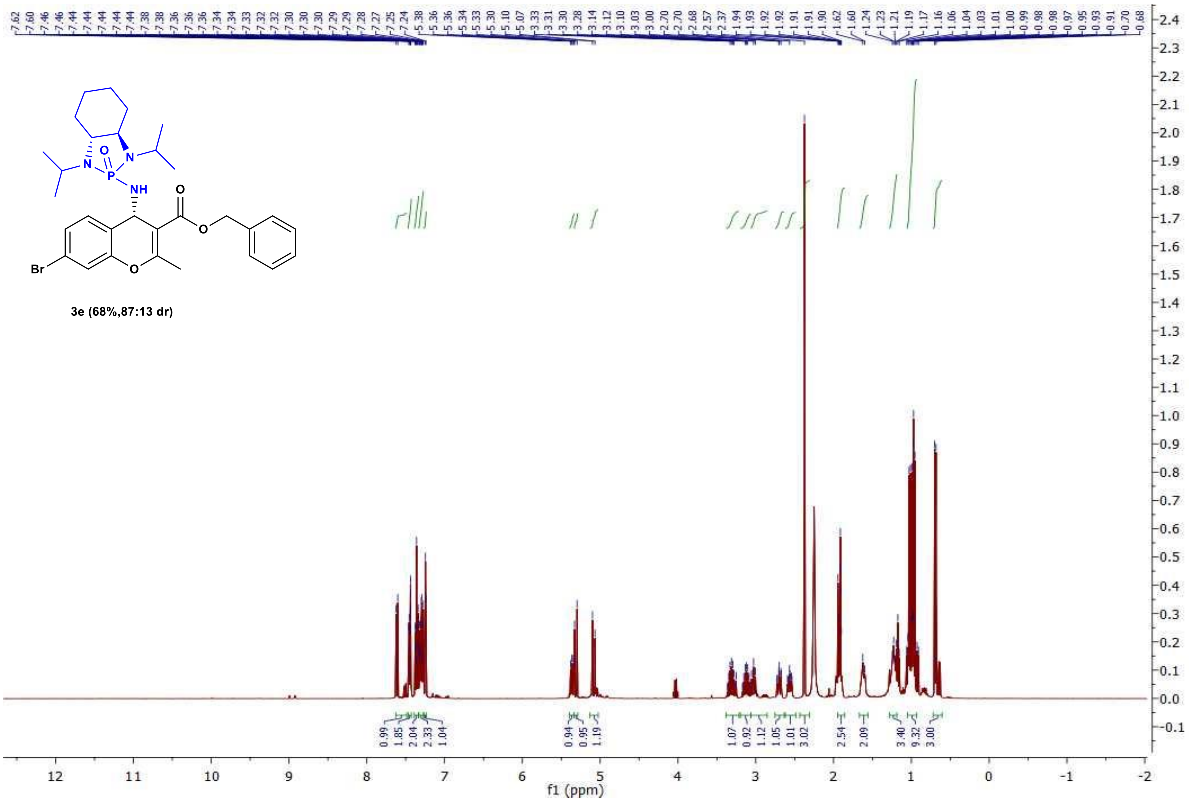

^{1}H NMR of Chromene **3e** (CD_3CN, 400 Hz)

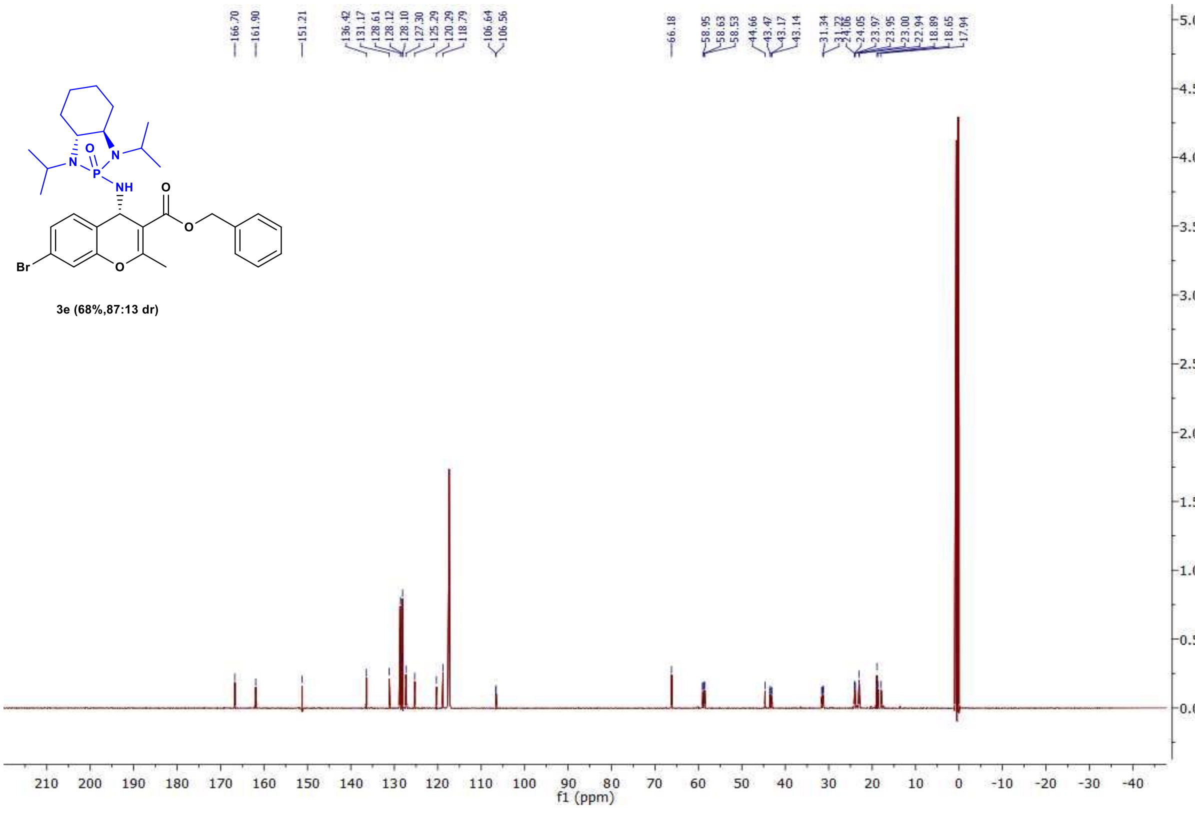

^{31}C NMR of Chromene **3e** (CD_3CN, 101 Hz)

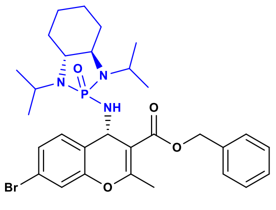

3e (68%,87:13 dr)

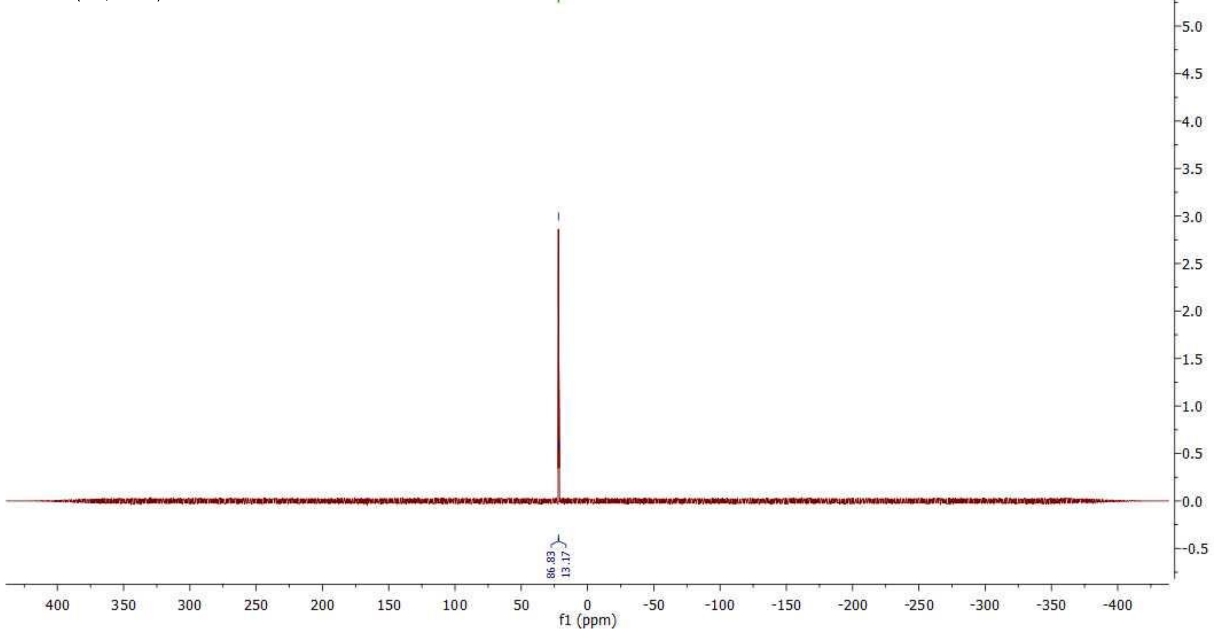

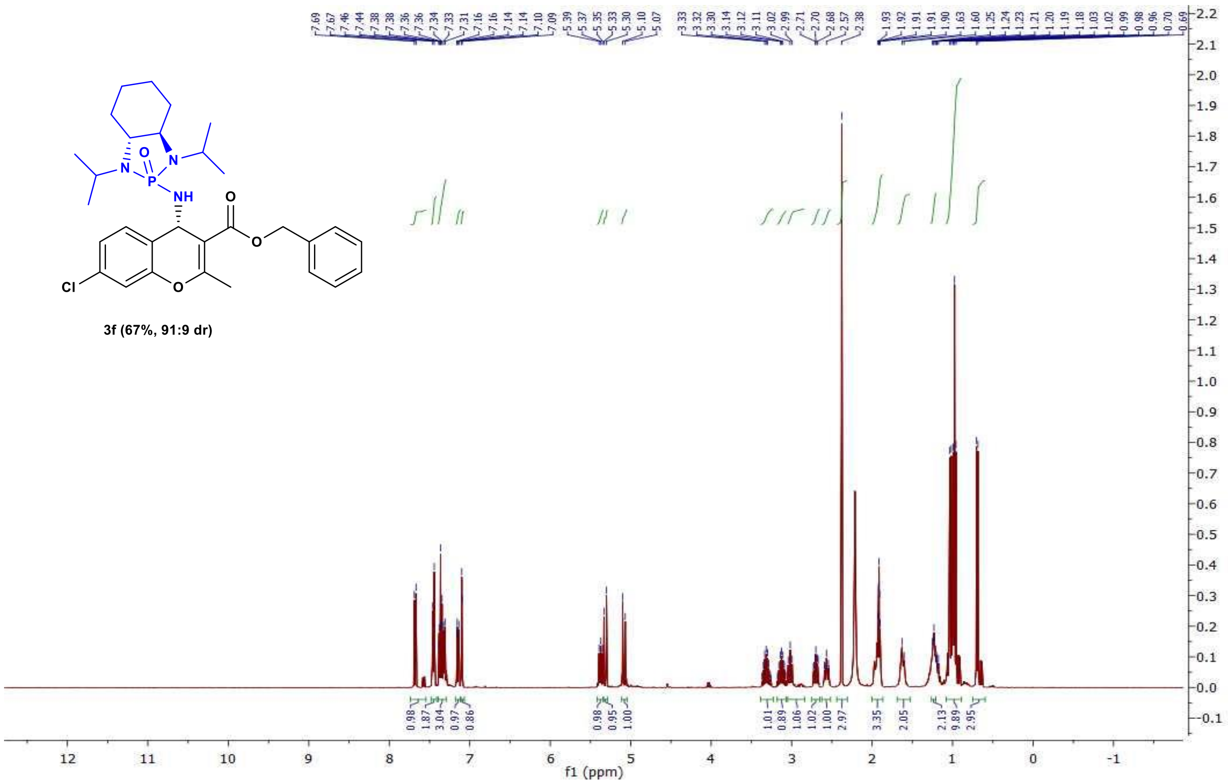

^{1}H NMR of Chromene **3f** (CD$_3$CN, 400 Hz)

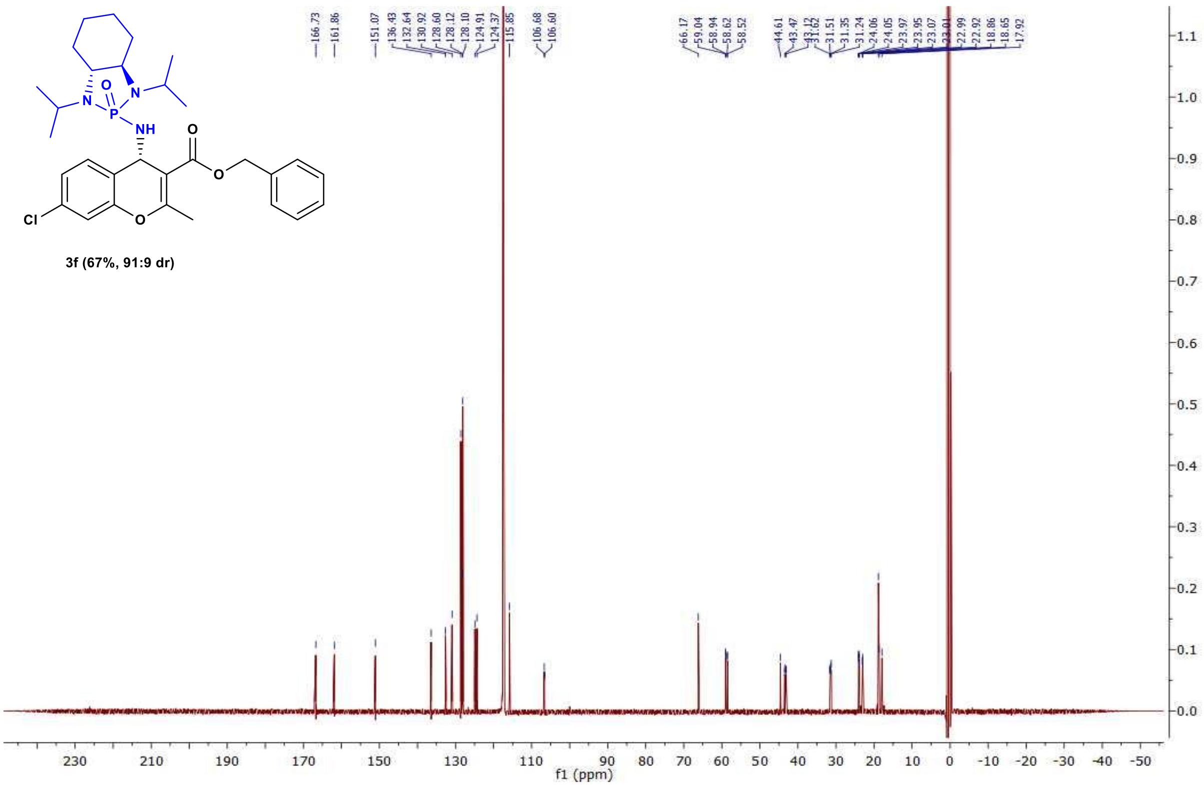

^{31}C NMR of Chromene **3f** (CD$_3$CN, 101 Hz)

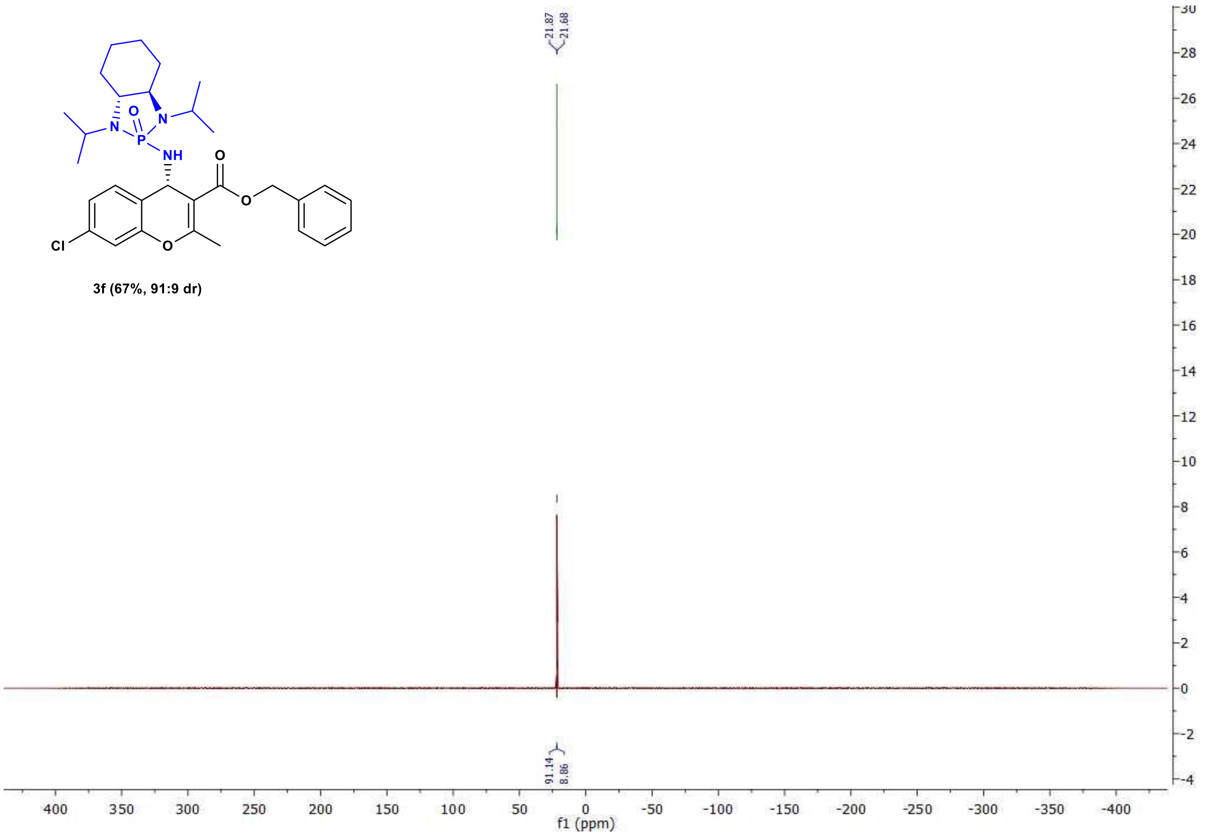
3f (67%, 91:9 dr)
21.87
21.68
91.14
8.86
f1 (ppm)

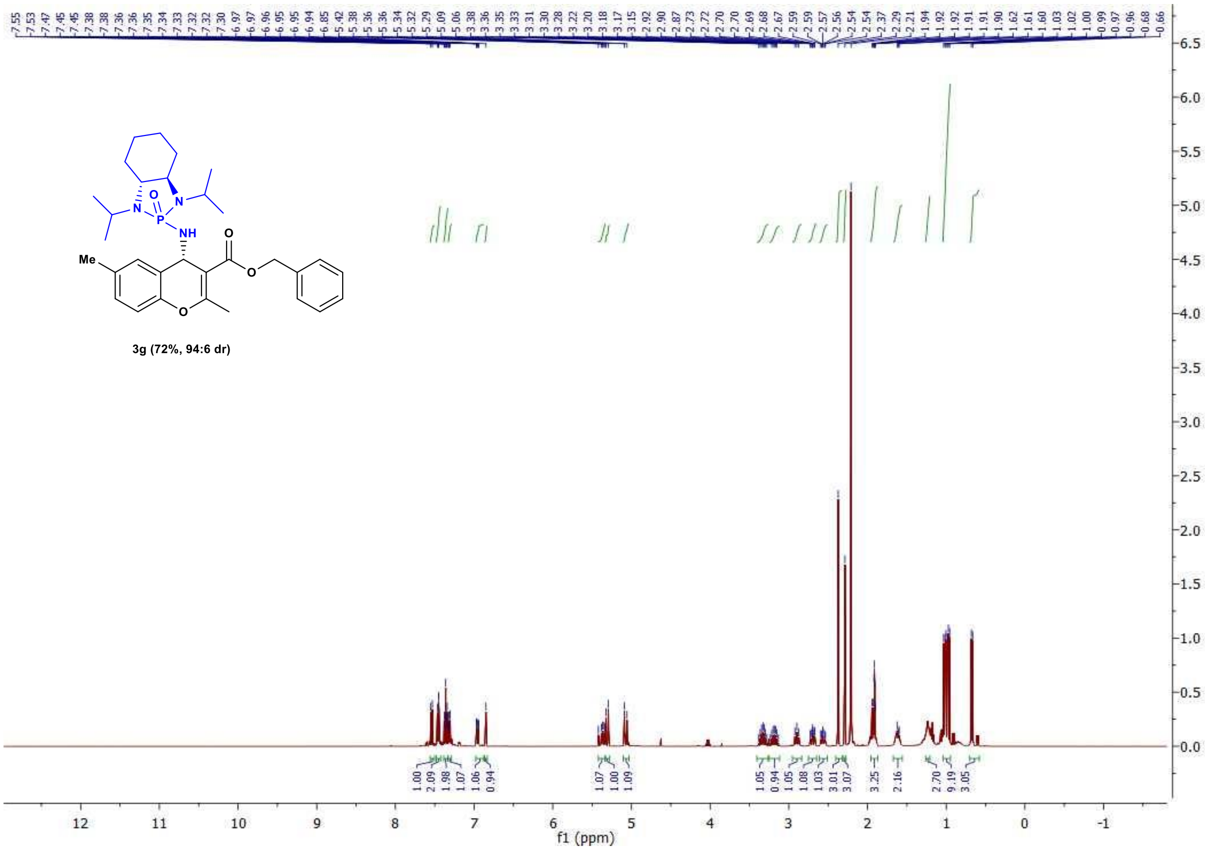

^{1}H NMR of Chromene **3g** (CD3CN, 400 Hz)

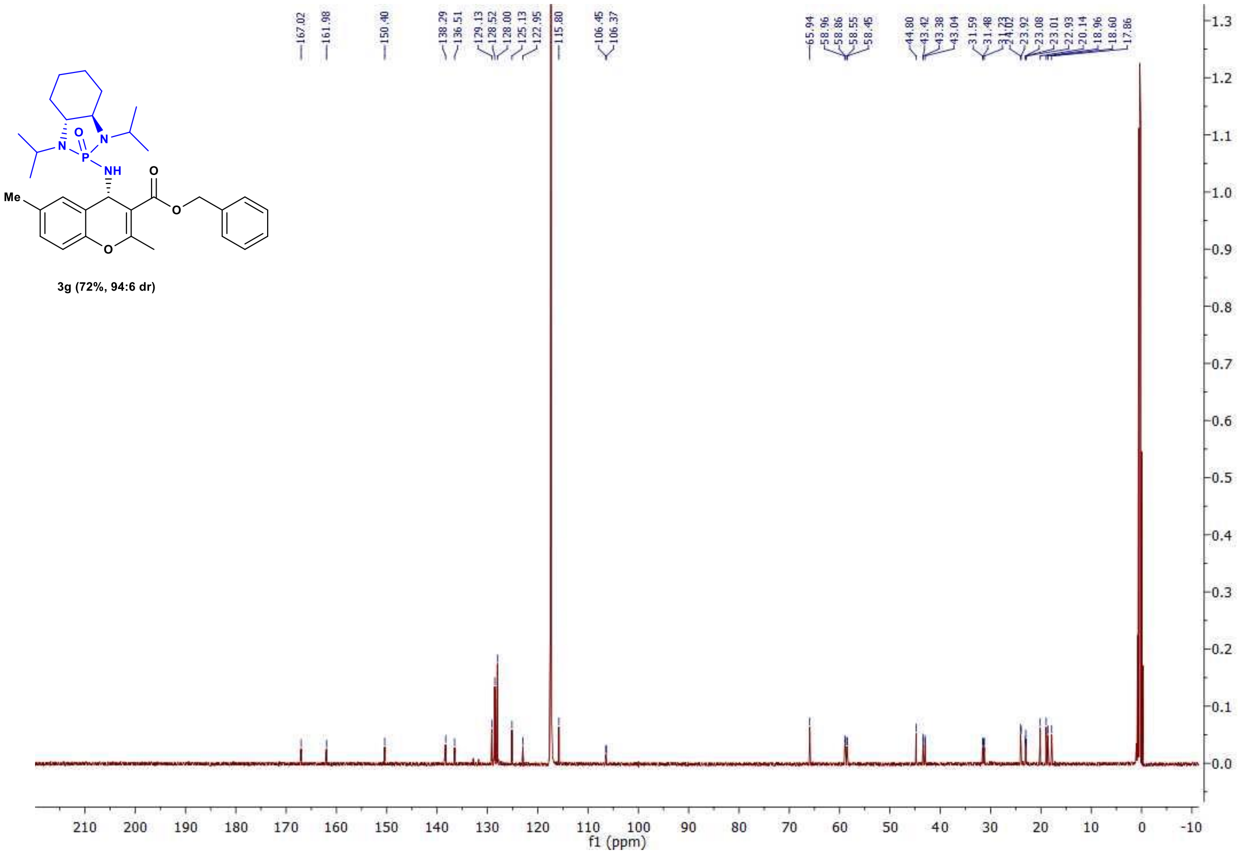

[31]C NMR of Chromene **3g** (CD3CN, 101 Hz)

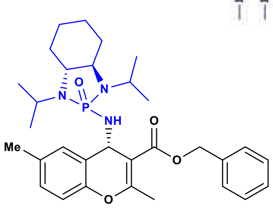
Me
O
N
P
NH
O
O

3g (72%, 94:6 dr)

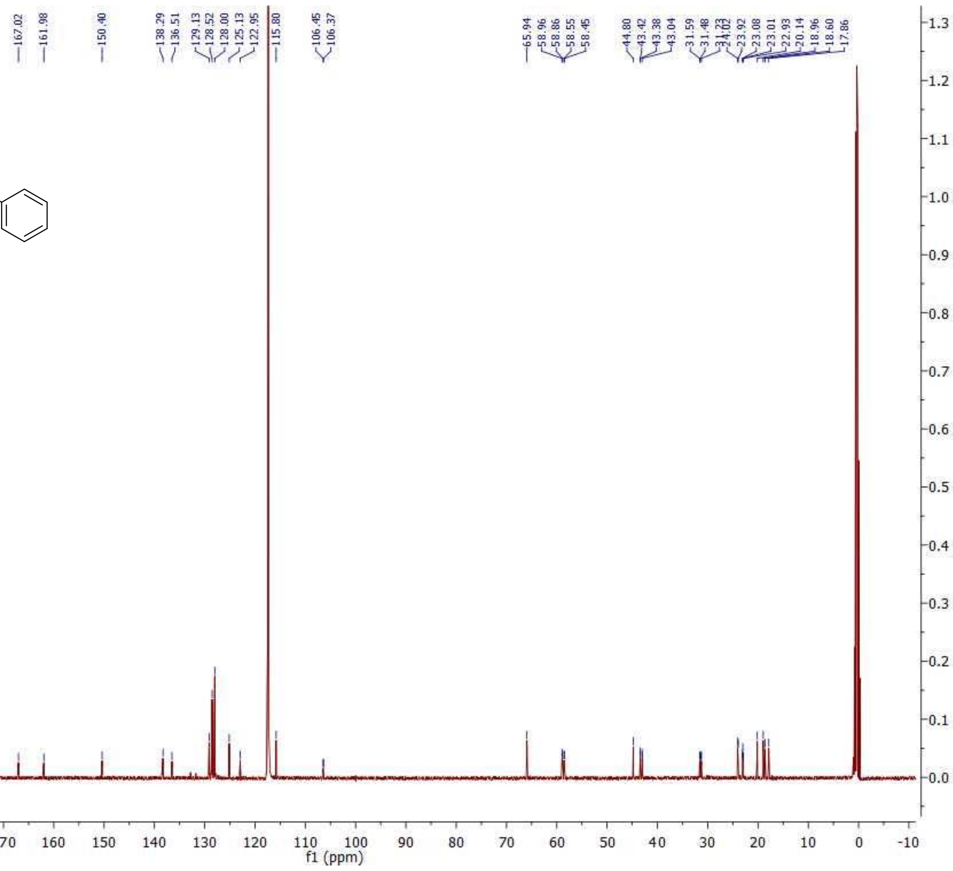
167.02
161.98
150.40
138.29
136.51
129.13
128.52
128.00
125.13
122.95
115.80
106.45
106.37
65.94
58.96
58.86
58.55
58.45
44.80
43.42
43.38
43.04
31.59
31.48
23.92
23.08
23.01
22.93
20.14
18.96
18.60
17.86
f1 (ppm)

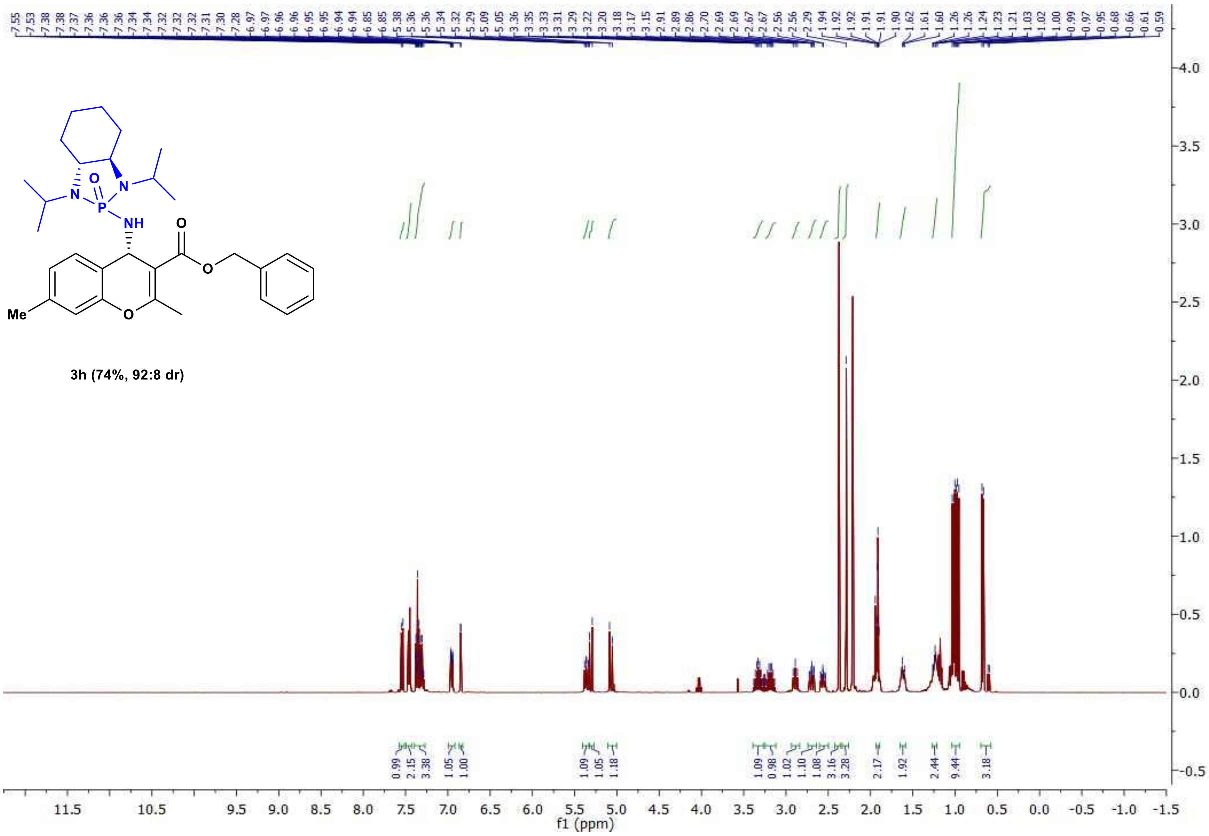

^{1}H NMR of Chromene **3h** (CD_3CN, 400 Hz)

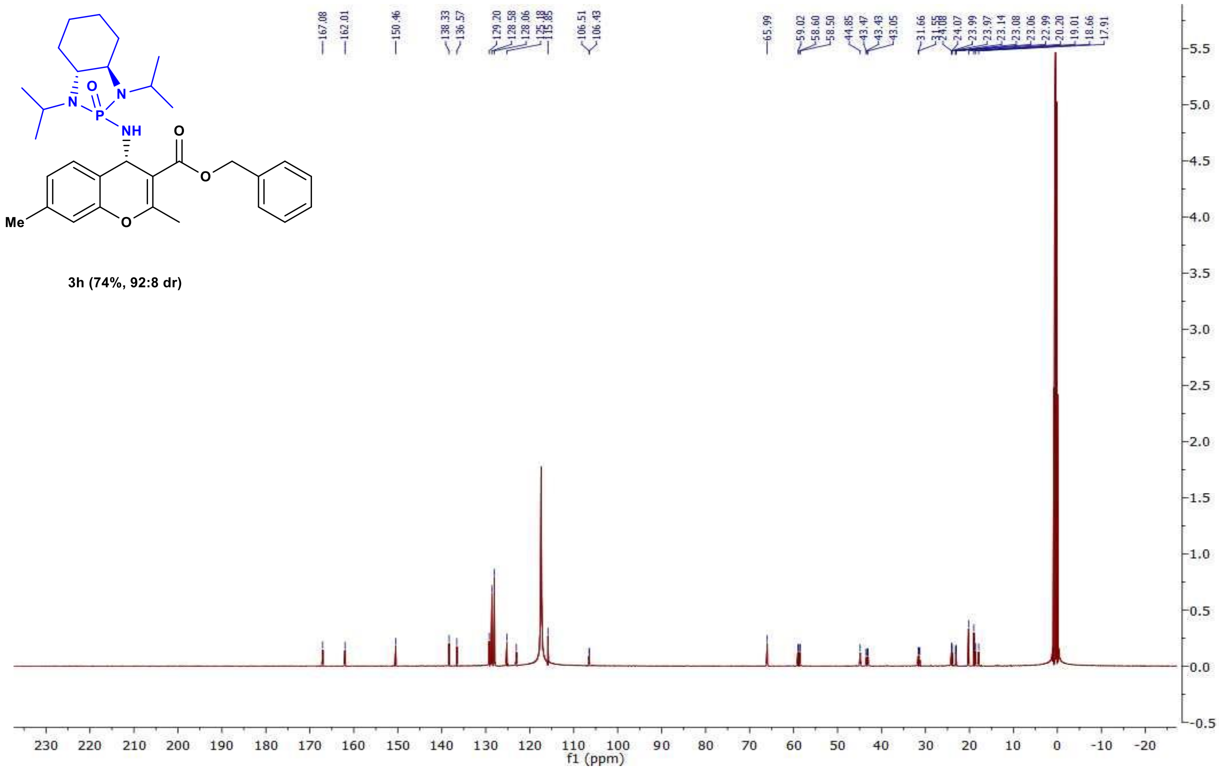

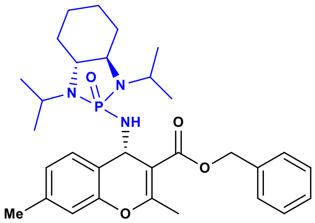

3h (74%, 92:8 dr)

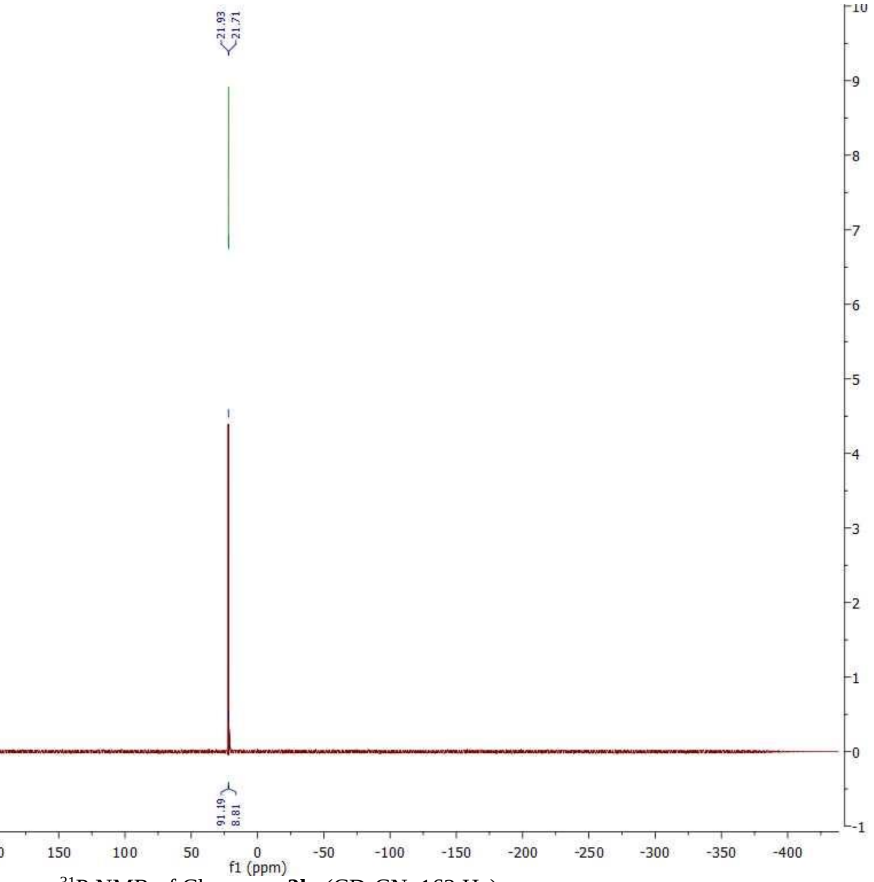

^{31}P NMR of Chromene **3h** (CD_3CN, 162 Hz)

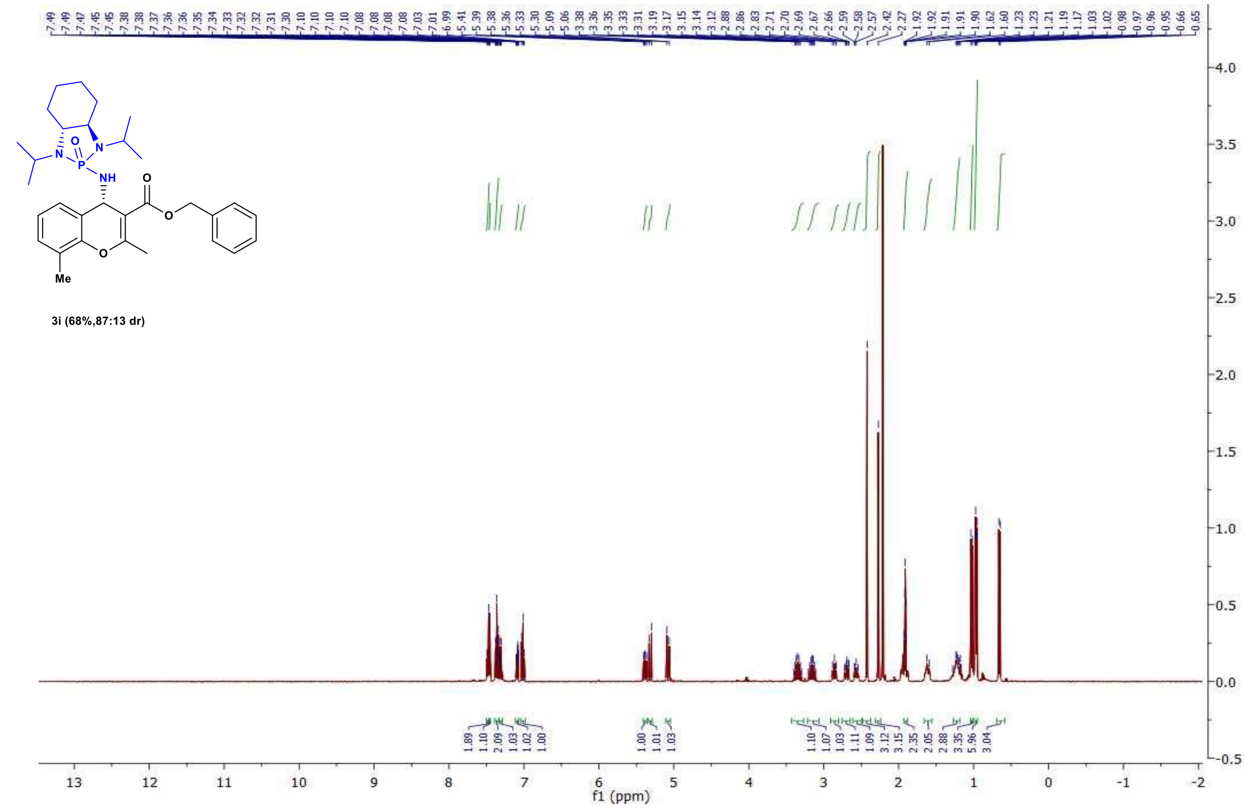

^{1}H NMR of Chromene **3i** (CD_3CN, 400 Hz)

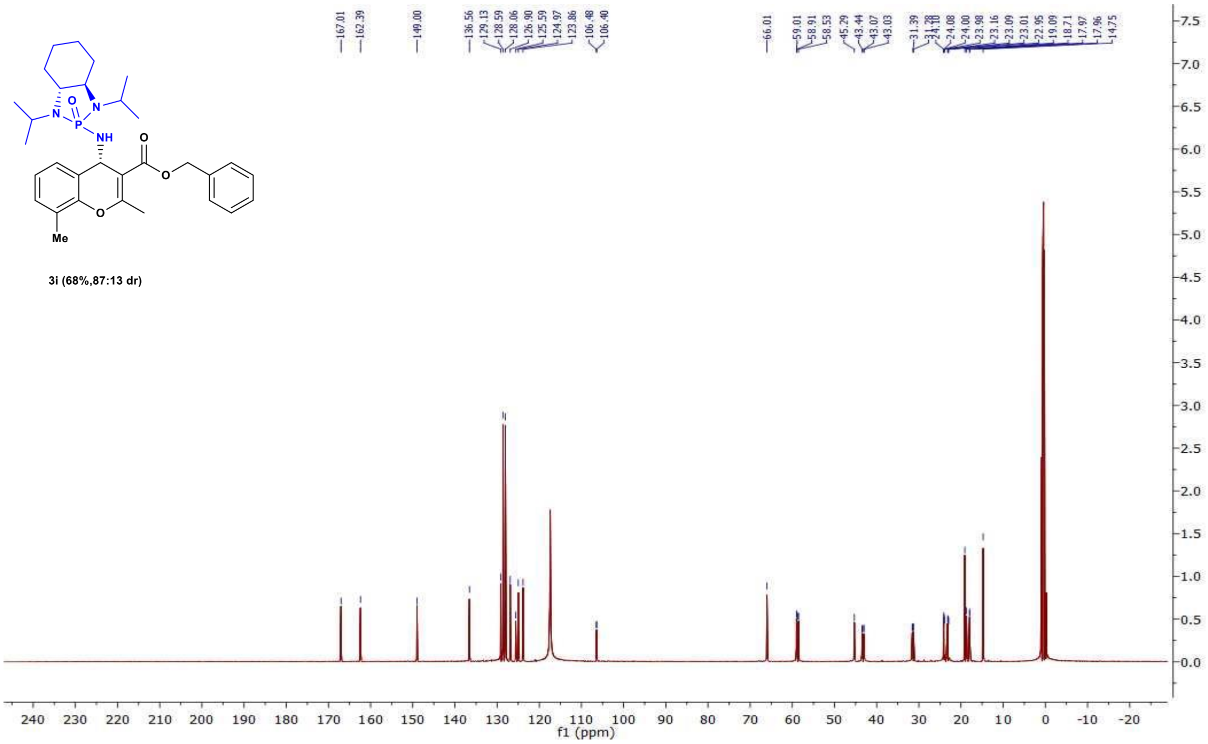

^{13}C NMR of Chromene **3i** (CD_3CN, 101 Hz)

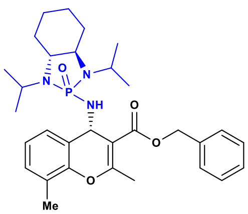

3i (68%,87:13 dr)

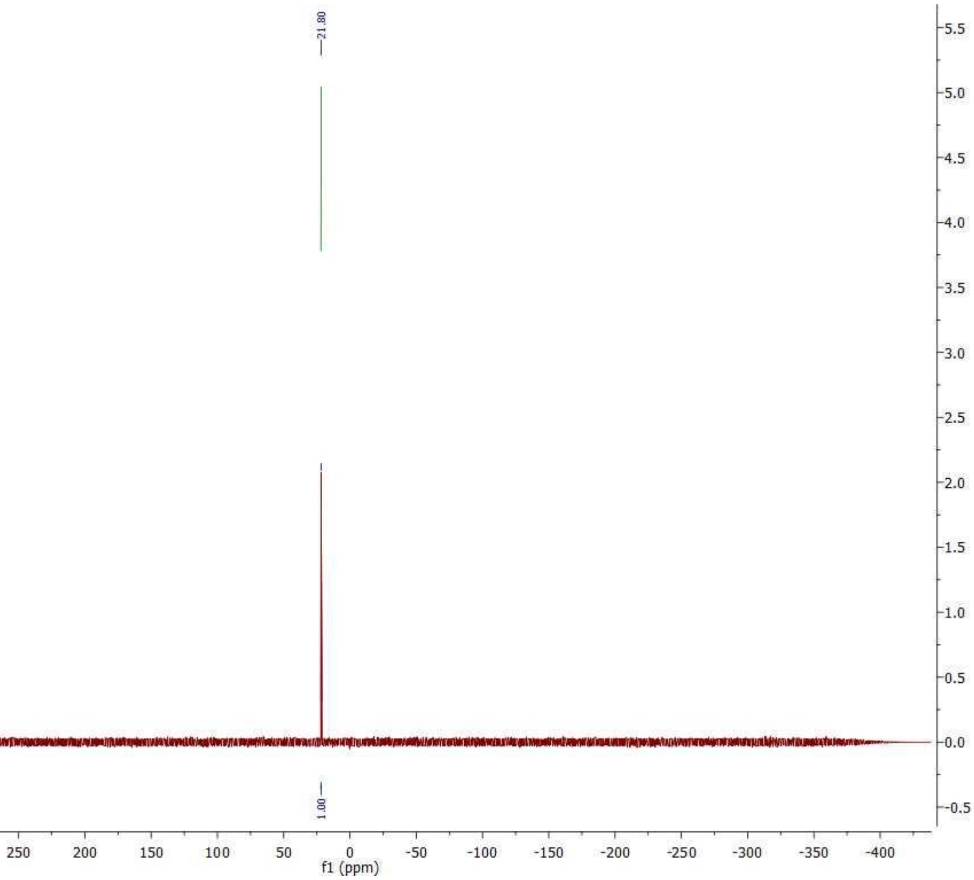

^{31}P NMR of Chromene **3i** (CD_3CN, 162 Hz)

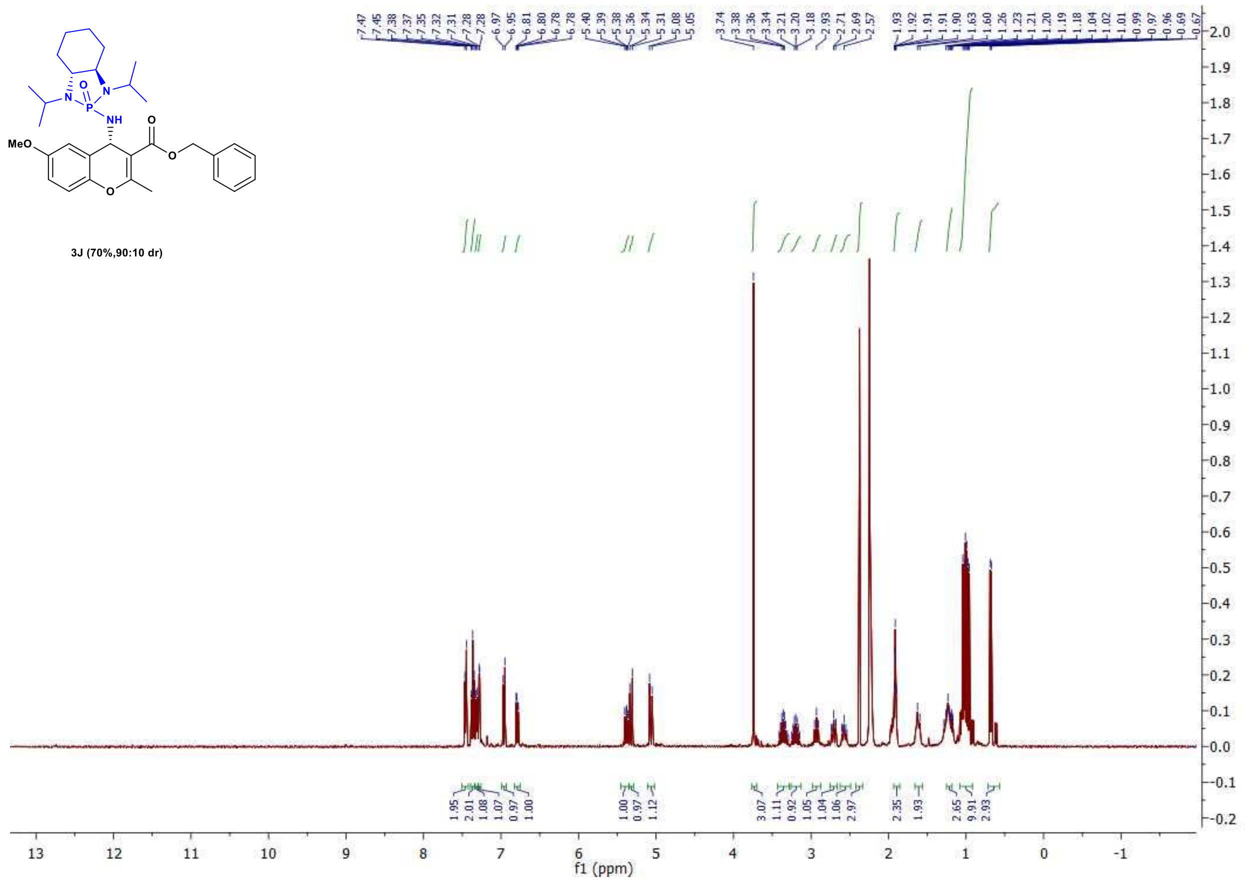

^{1}H NMR of Chromene **3j** (CD_3CN, 400 Hz)

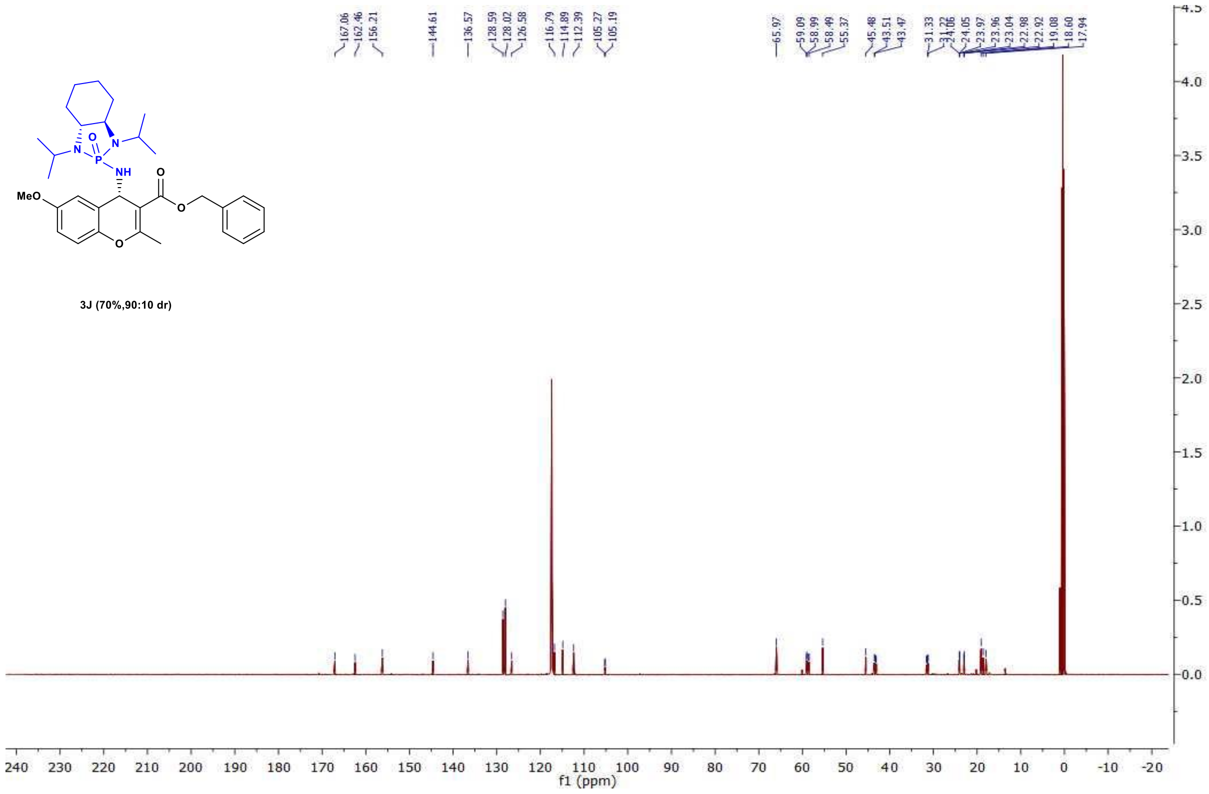

^{13}C NMR of Chromene **3j** (CD$_3$CN, 101 Hz)

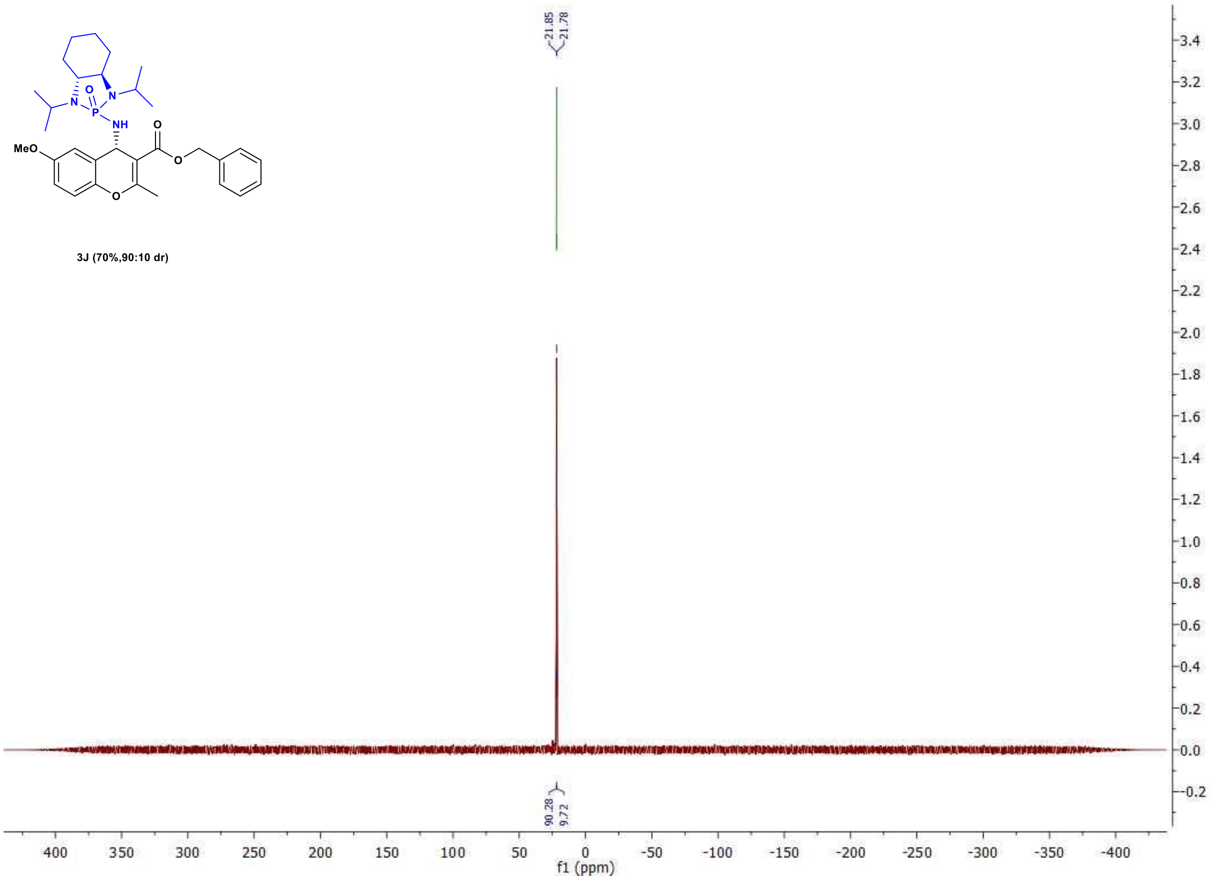

^{31}P NMR of Chromene **3j** (CD$_3$CN, 162 Hz)

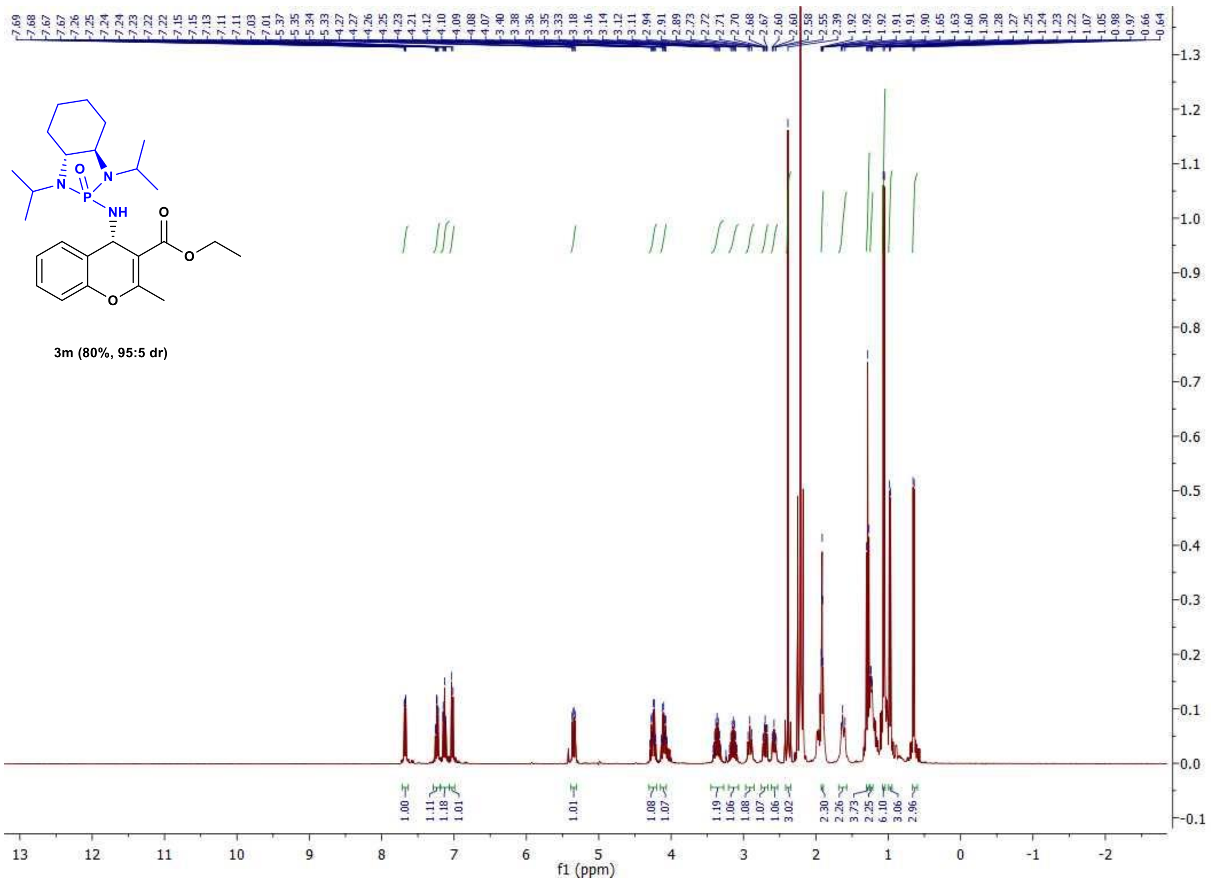

^{1}H NMR of Chromene **3m** (CD_3CN, 400 Hz)

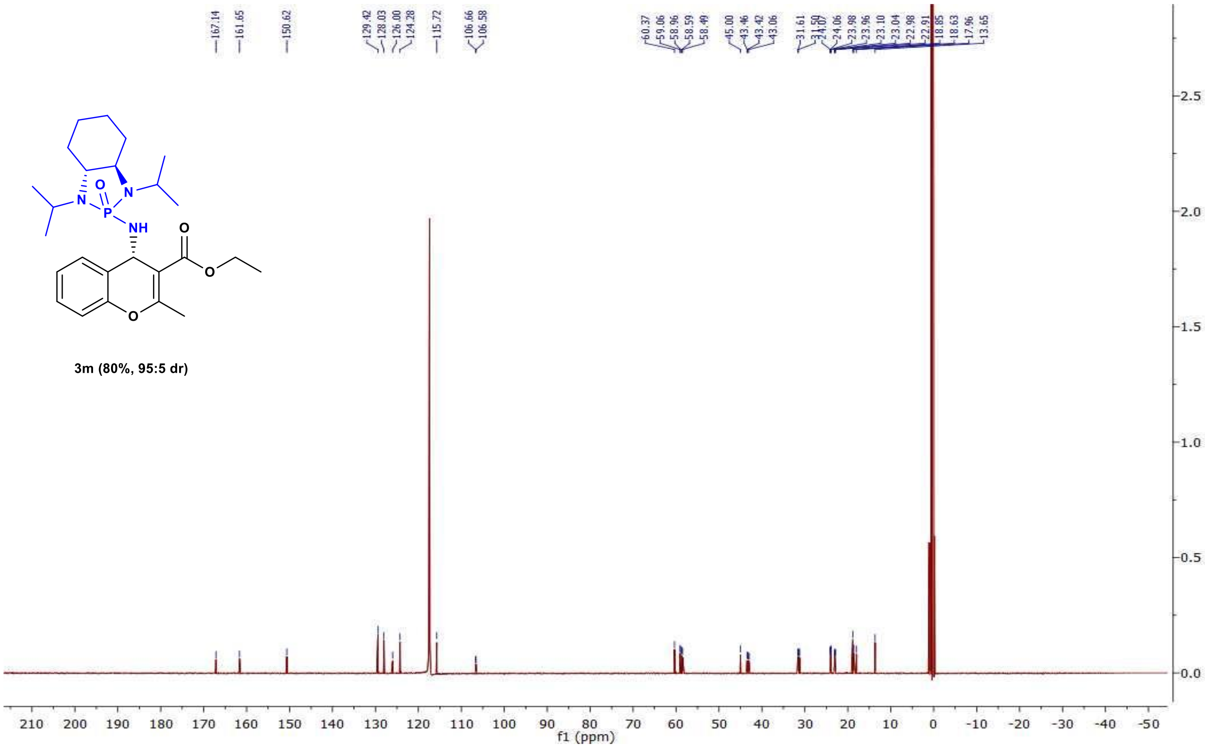

^{13}C NMR of Chromene **3m** (CD_3CN, 101 Hz)

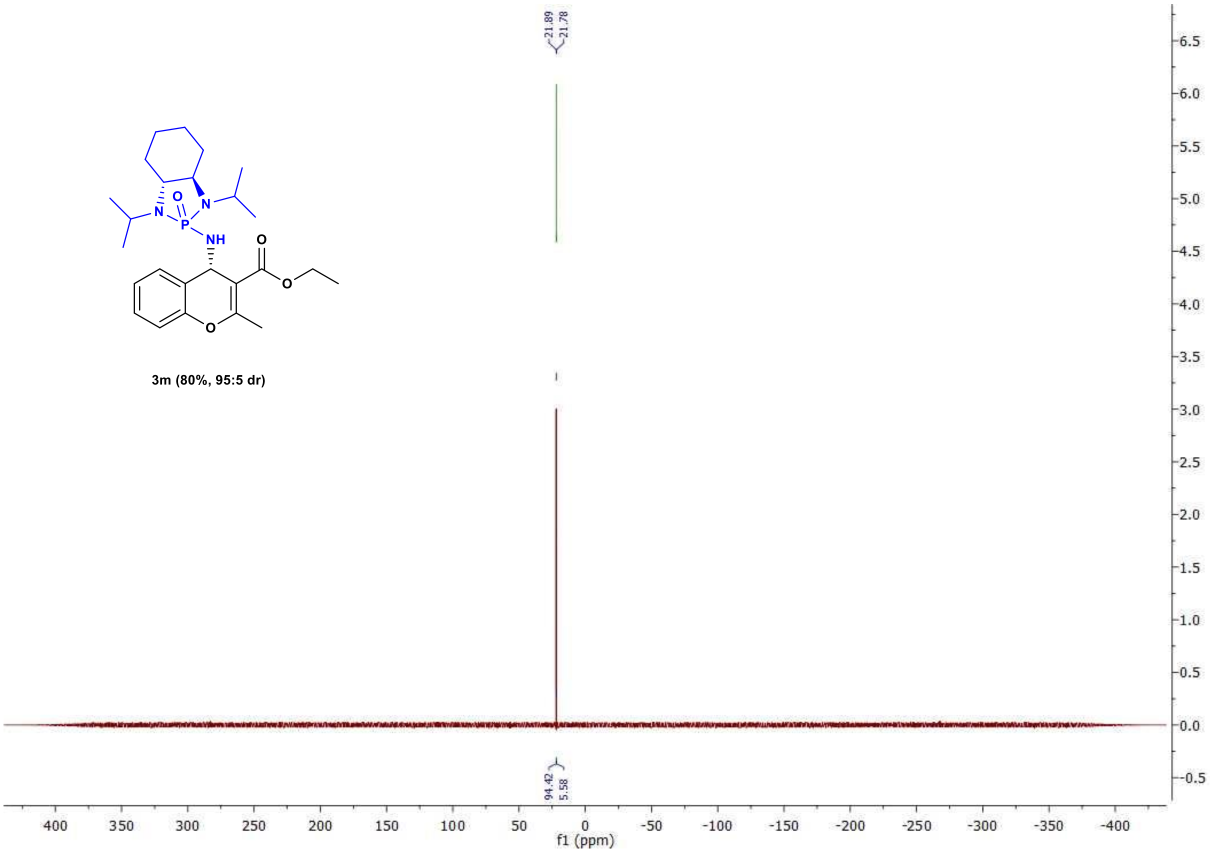

^{31}P NMR of Chromene **3m** (CD_3CN, 162 Hz)

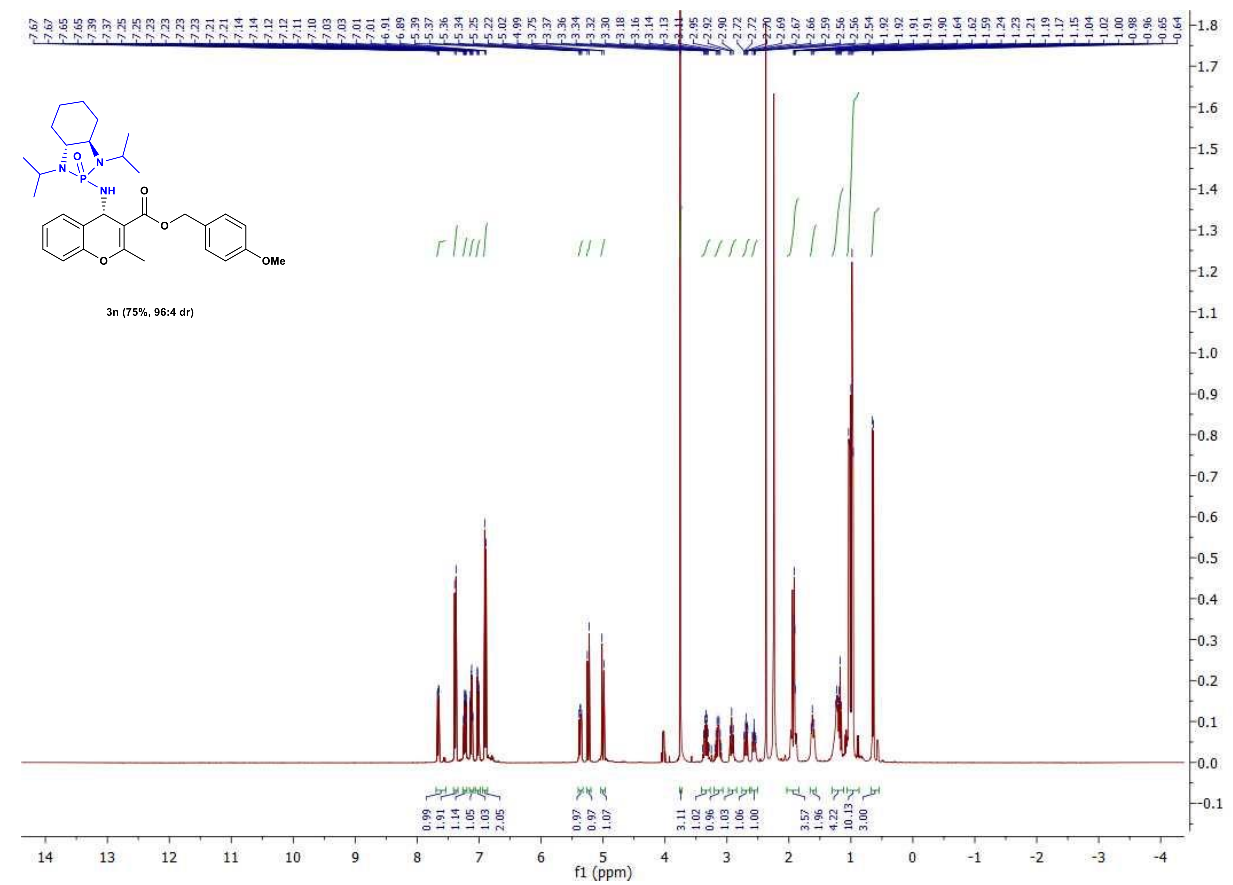

^{1}H NMR of Chromene **3n** (CD_3CN, 400 Hz)

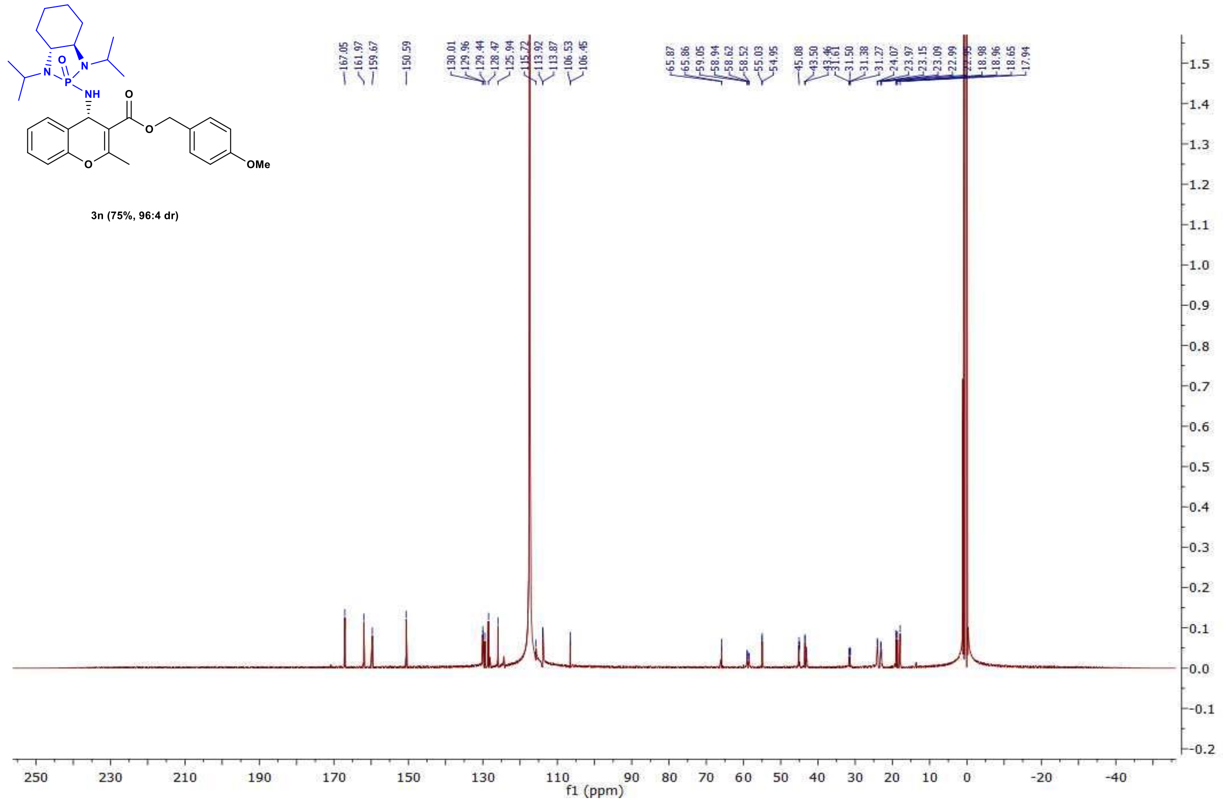

^{13}C NMR of Chromene **3n** (CD_3CN, 101 Hz)

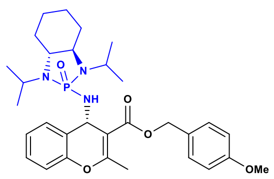

3n (75%, 96:4 dr)

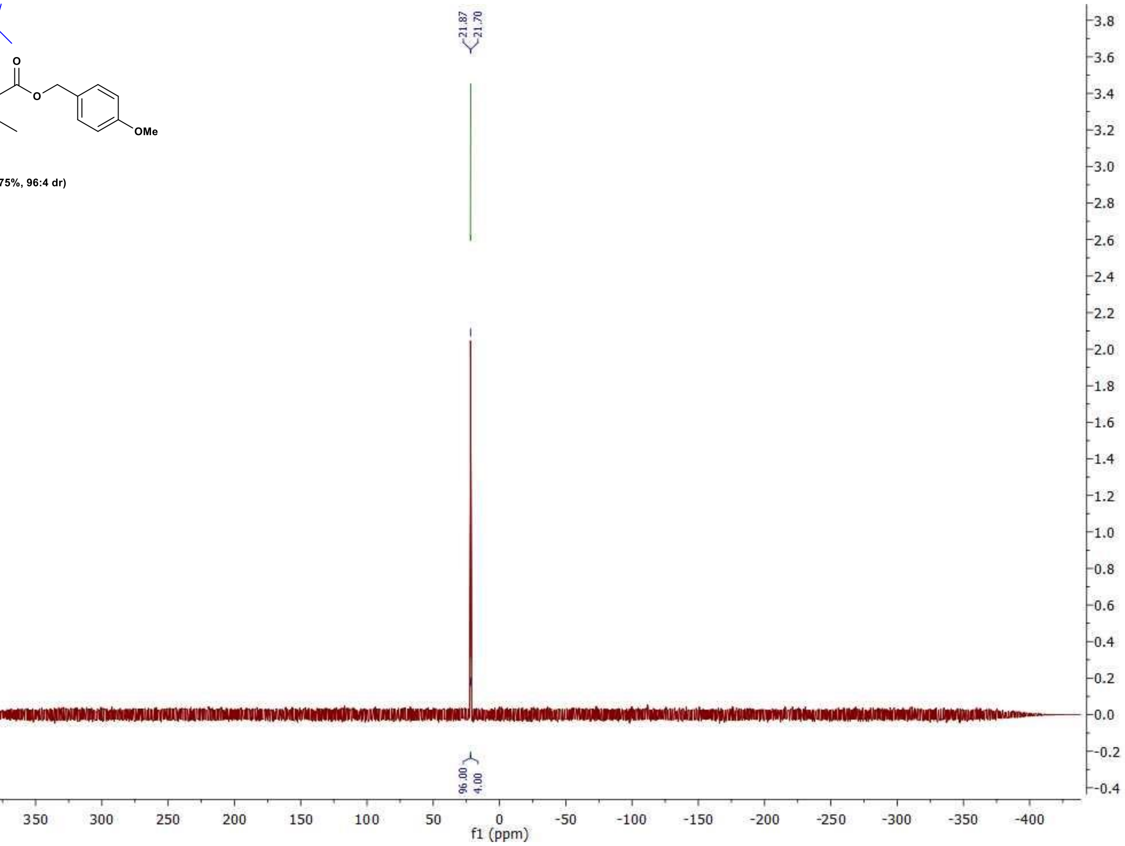

^{31}P NMR of Chromene **3n** (CD_3CN, 162 Hz)

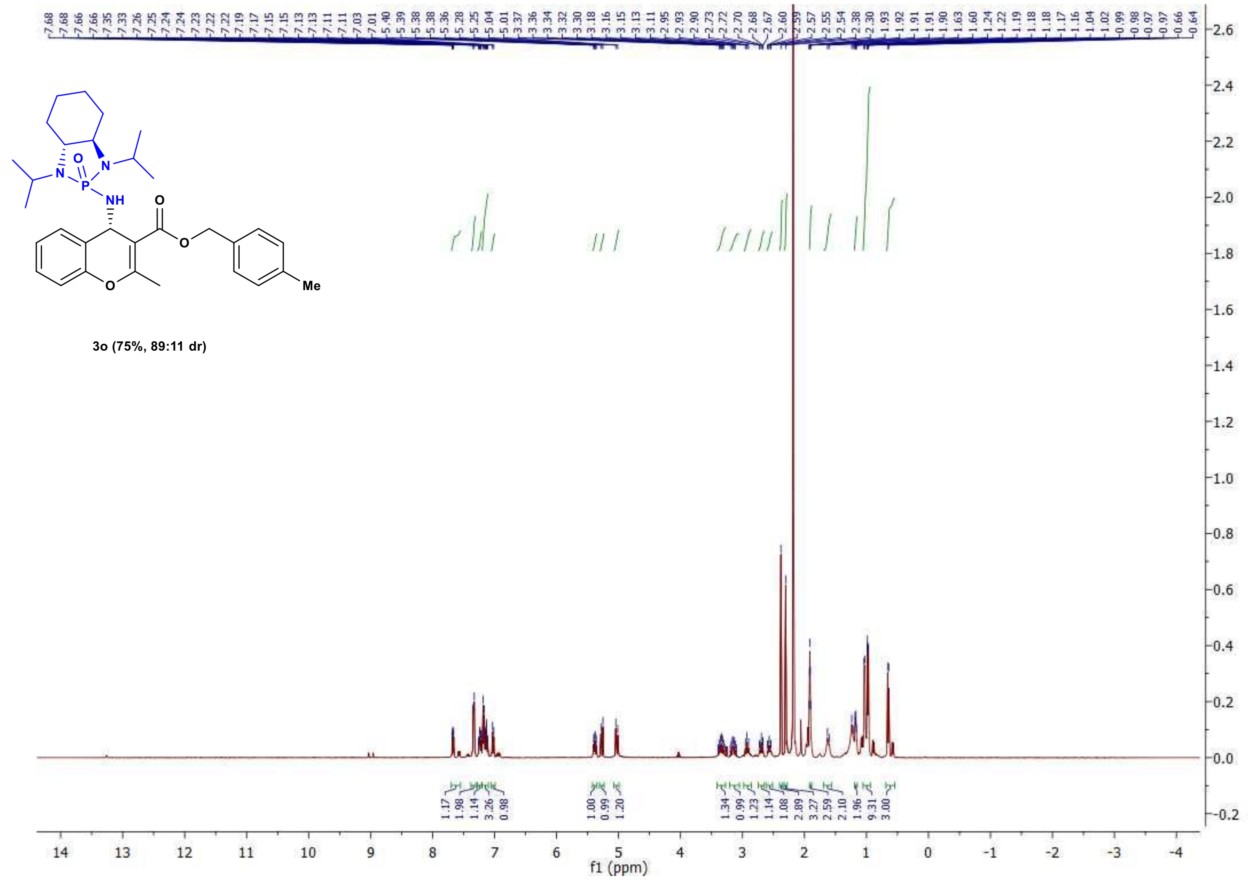

^{1}H NMR of Chromene **3o** (CD_3CN, 400 Hz)

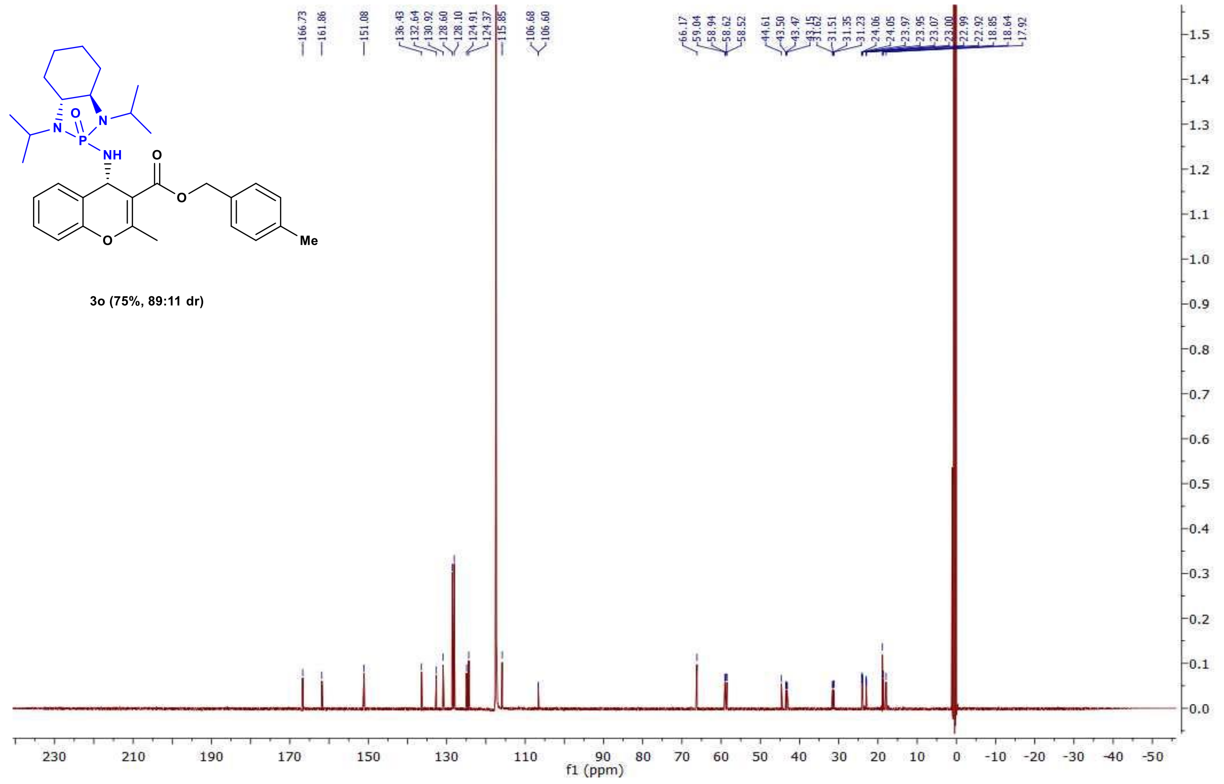

^{13}C NMR of Chromene **3o** (CD_3CN, 101 Hz)

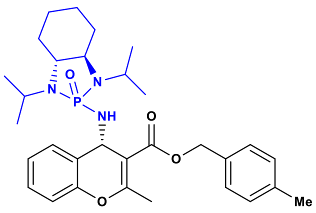

3o (75%, 89:11 dr)

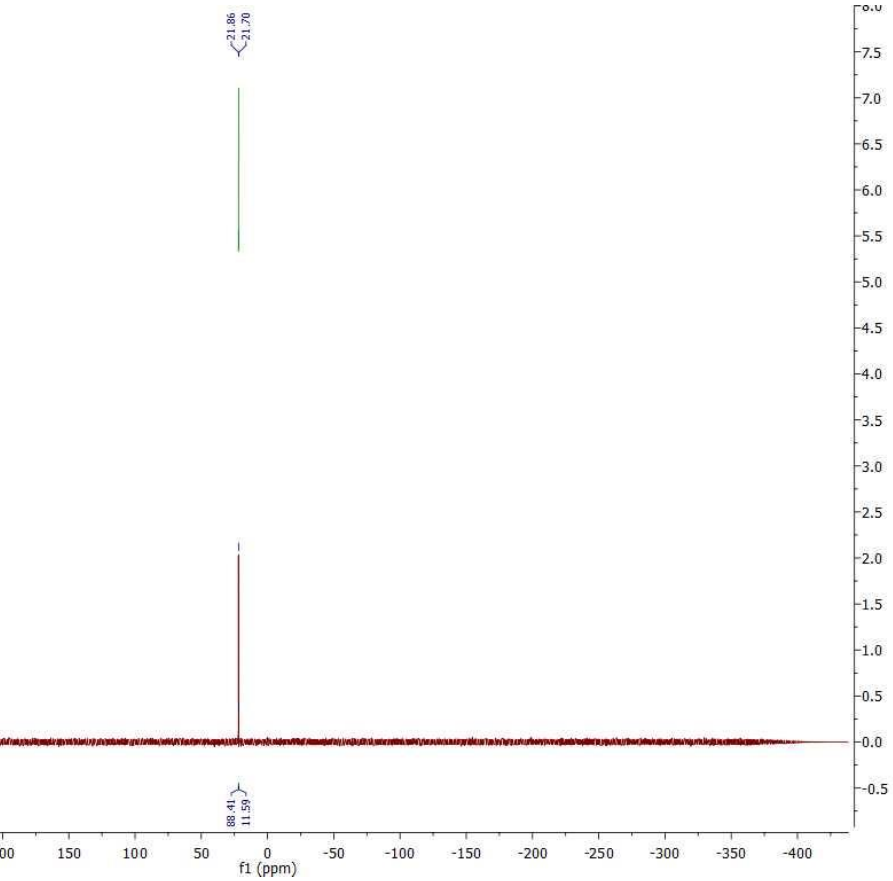

^{31}P NMR of Chromene **3o** (CD_3CN, 162 Hz)

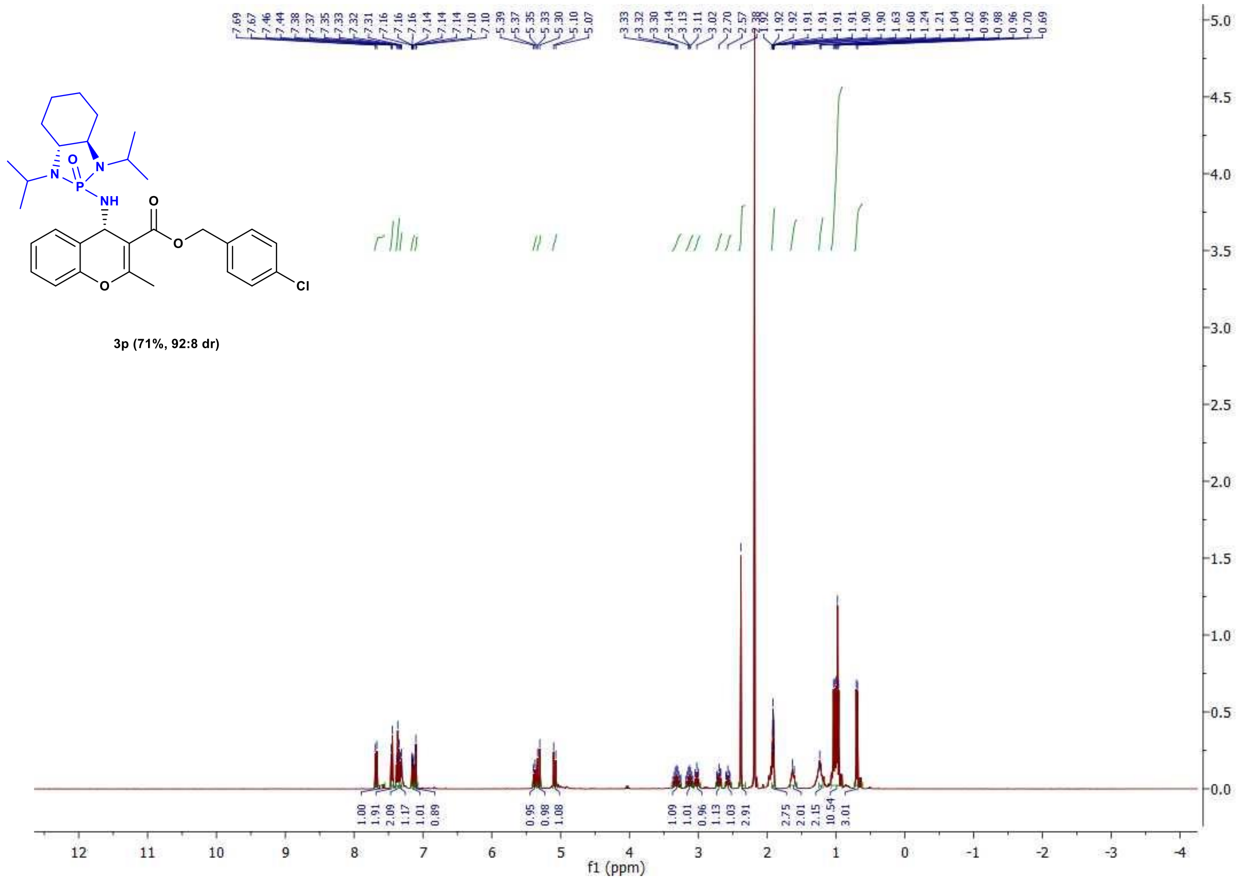

^{1}H NMR of Chromene **3p** (CD_3CN, 400 Hz)

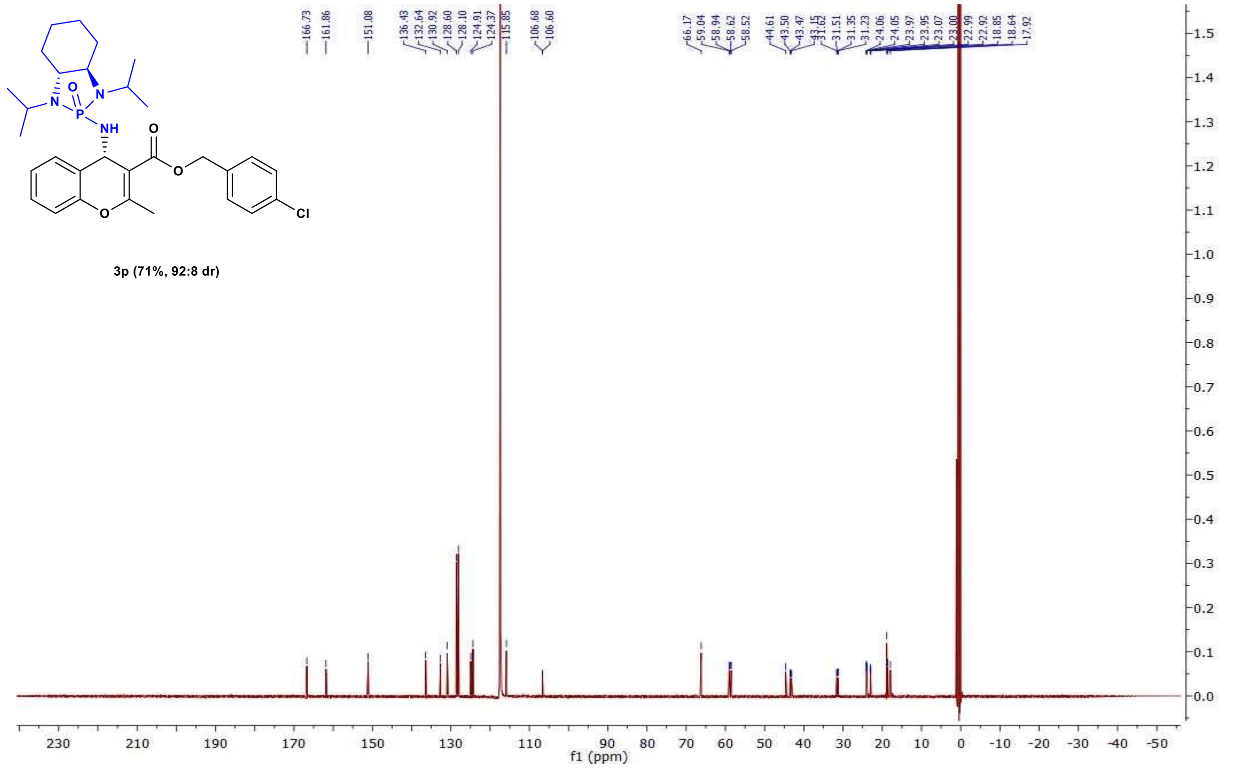

^{13}C NMR of Chromene **3p** (CD_3CN, 101 Hz)

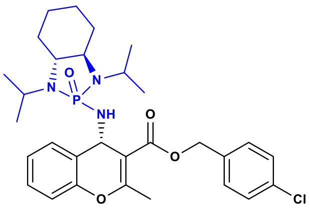

3p (71%, 92:8 dr)

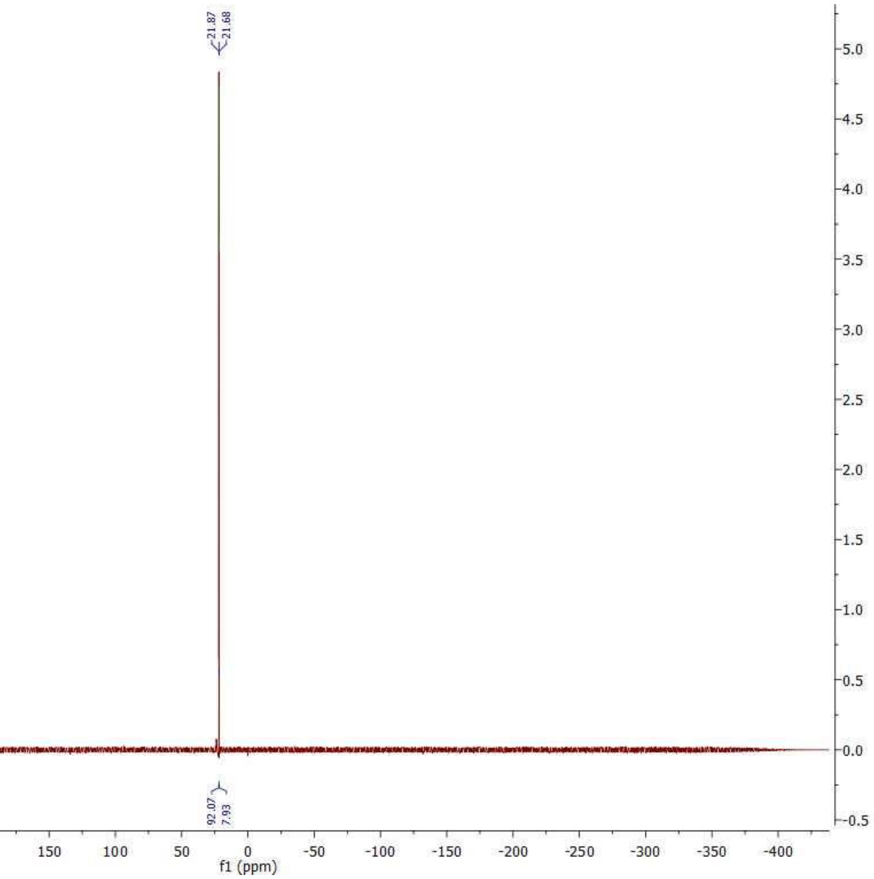

^{31}P NMR of Chromene **3p** (CD_3CN, 162 Hz)

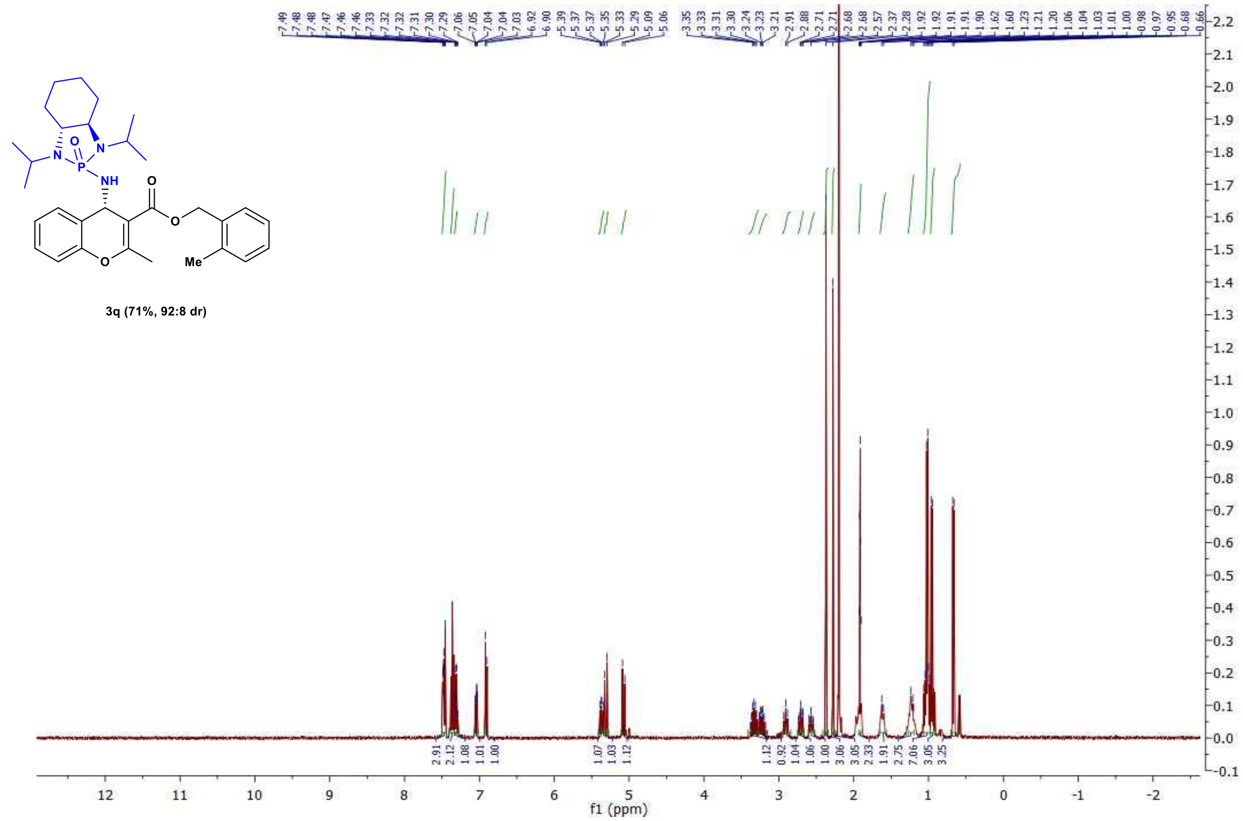

^{1}H NMR of Chromene **3q** (CD_3CN, 400 Hz)

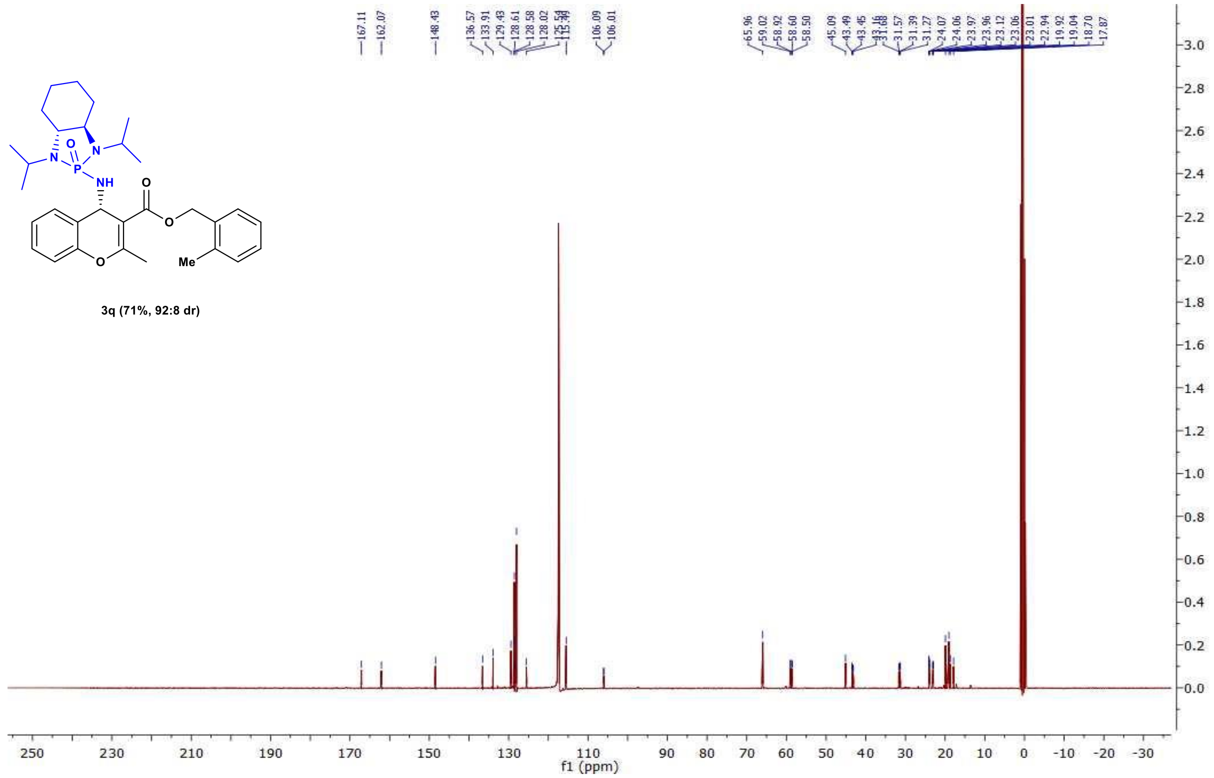

^{13}C NMR of Chromene **3q** (CD_3CN, 101 Hz)

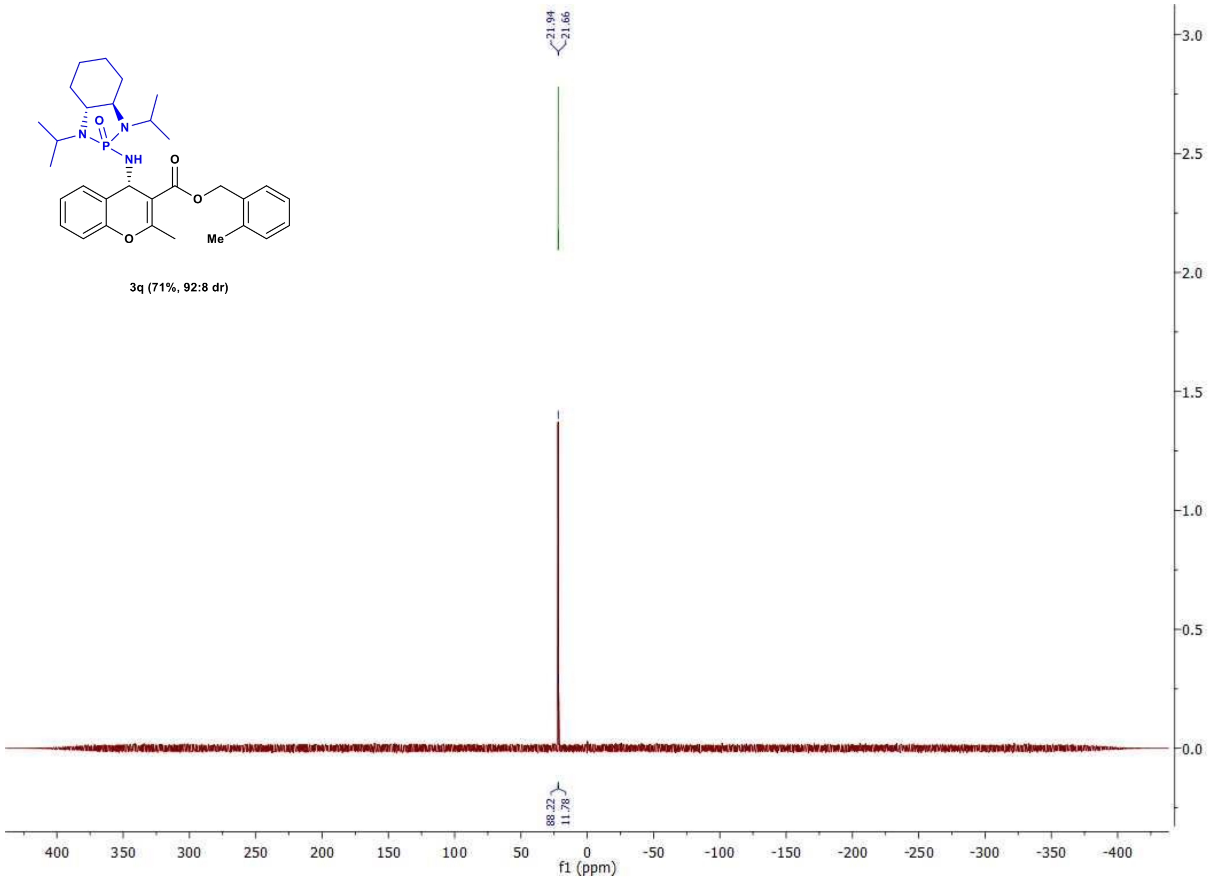

^{31}P NMR of Chromene **3q** (CD_3CN, 162 Hz)

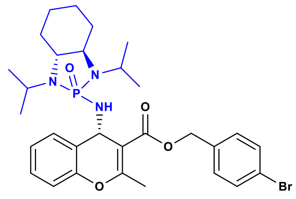

3r (69%, 91:9 dr)

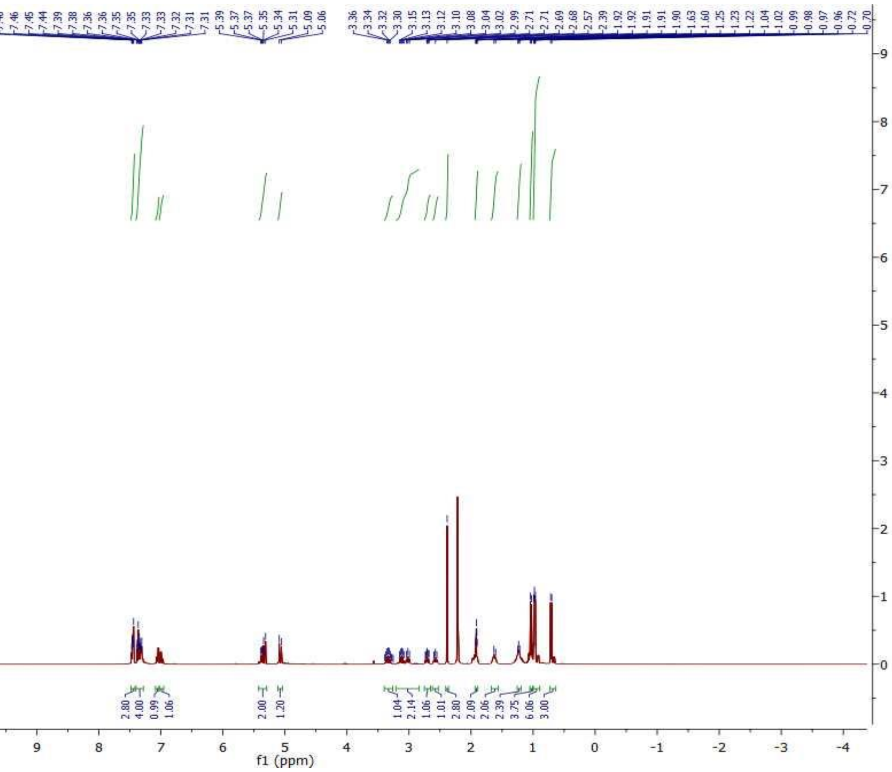

^{1}H NMR of Chromene **3r** (CD_3CN, 400 Hz)

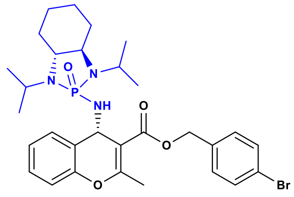

3r (69%, 91:9 dr)

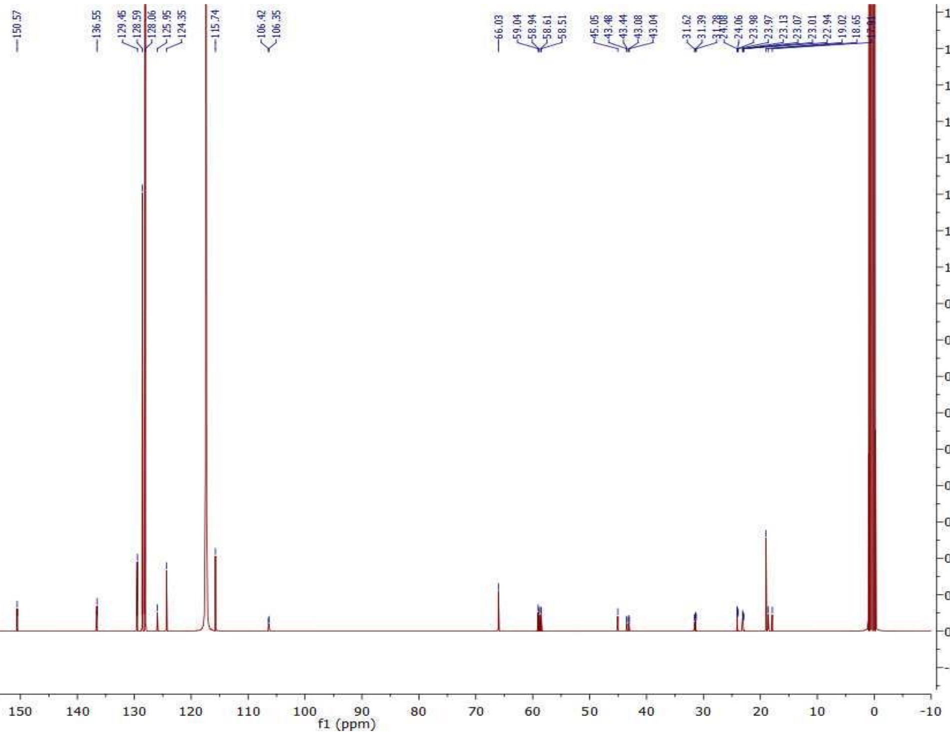

^{13}C NMR of Chromene **3r** (CD_3CN, 101 Hz)

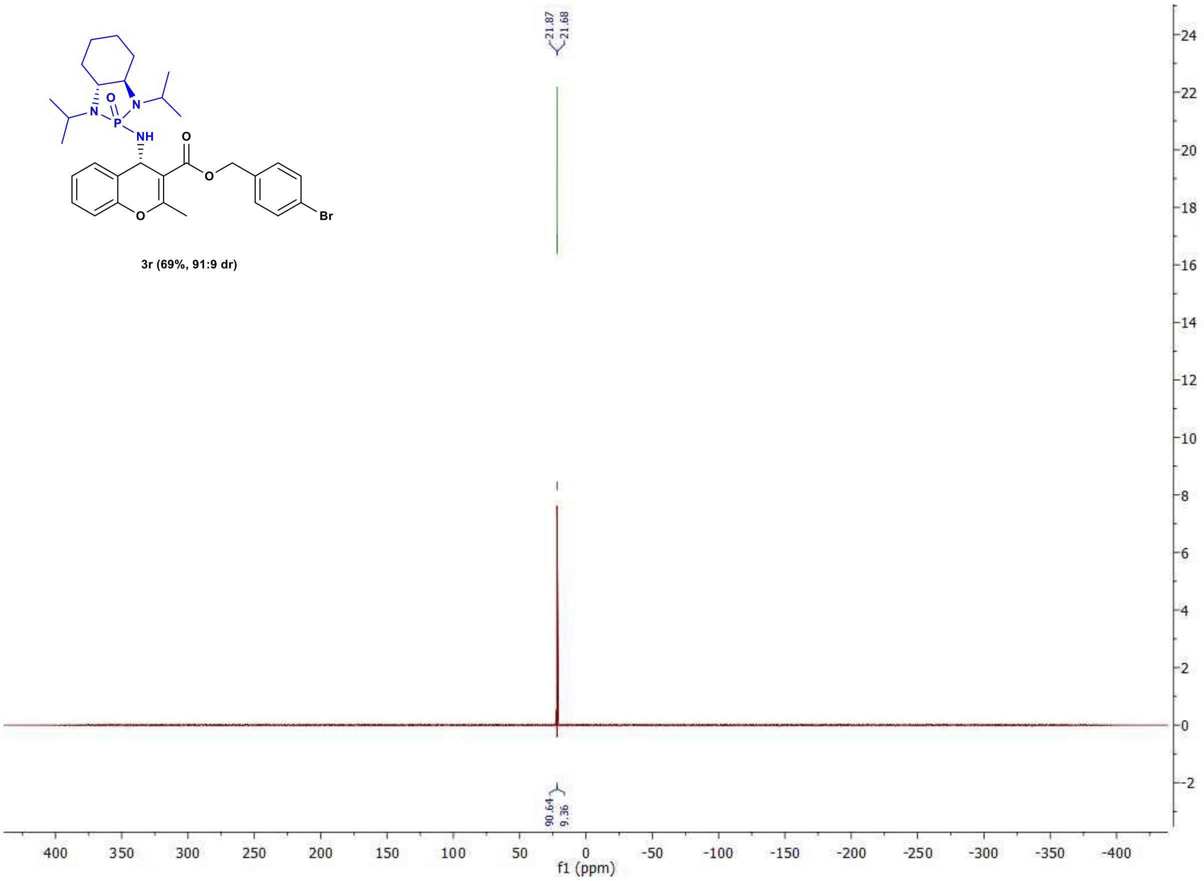

^{31}P NMR of Chromene **3r** (CD_3CN, 162 Hz)

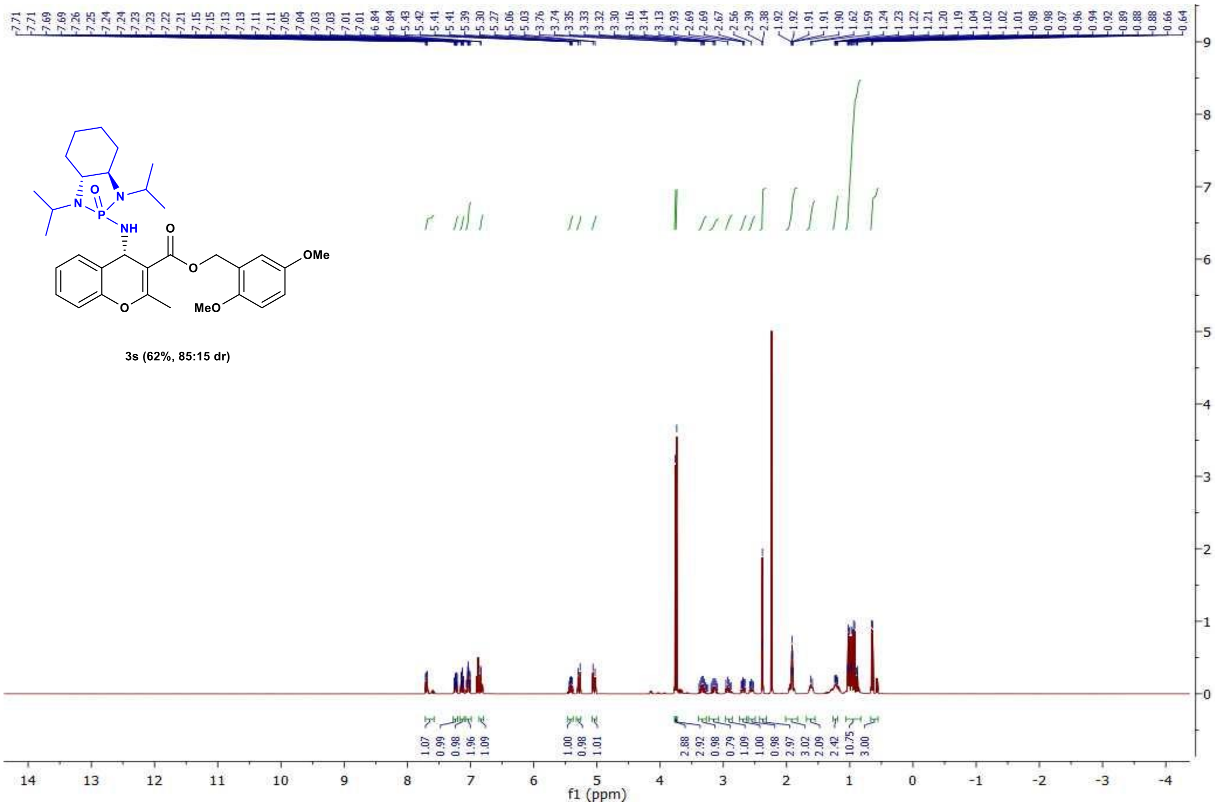

^{1}H NMR of Chromene **3s** (CD_3CN, 400 Hz)

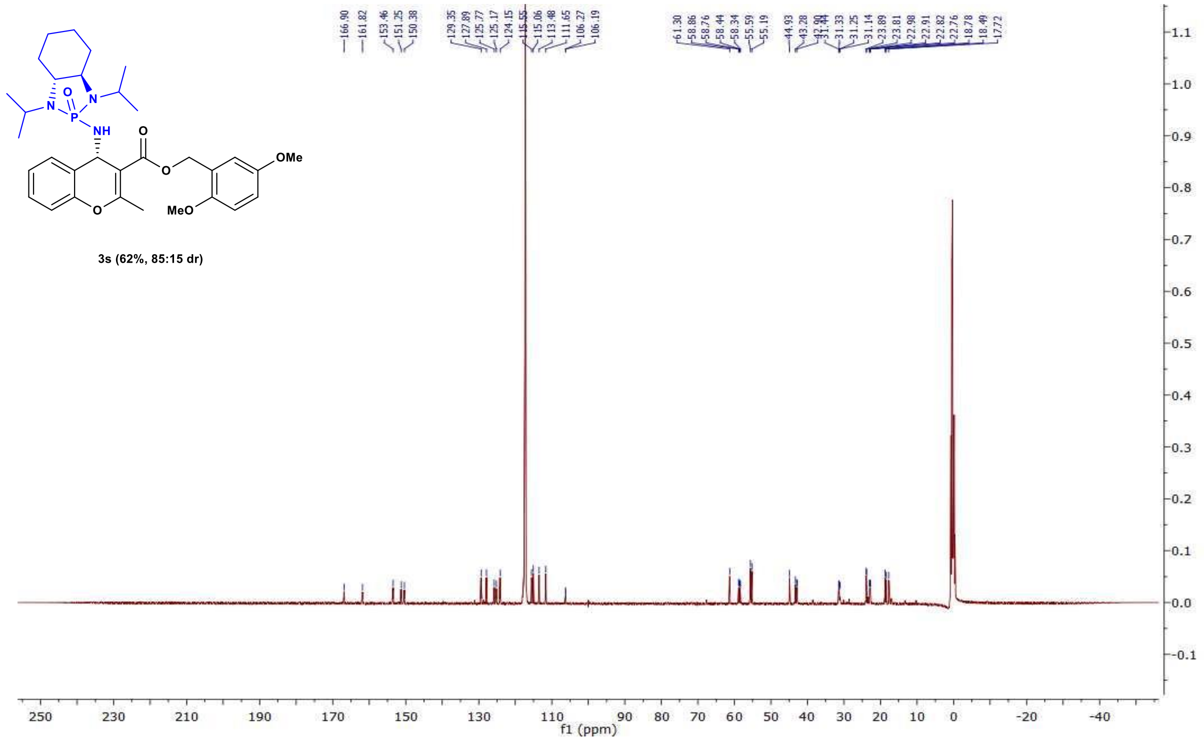

^{13}C NMR of Chromene **3s** (CD_3CN, 101 Hz)

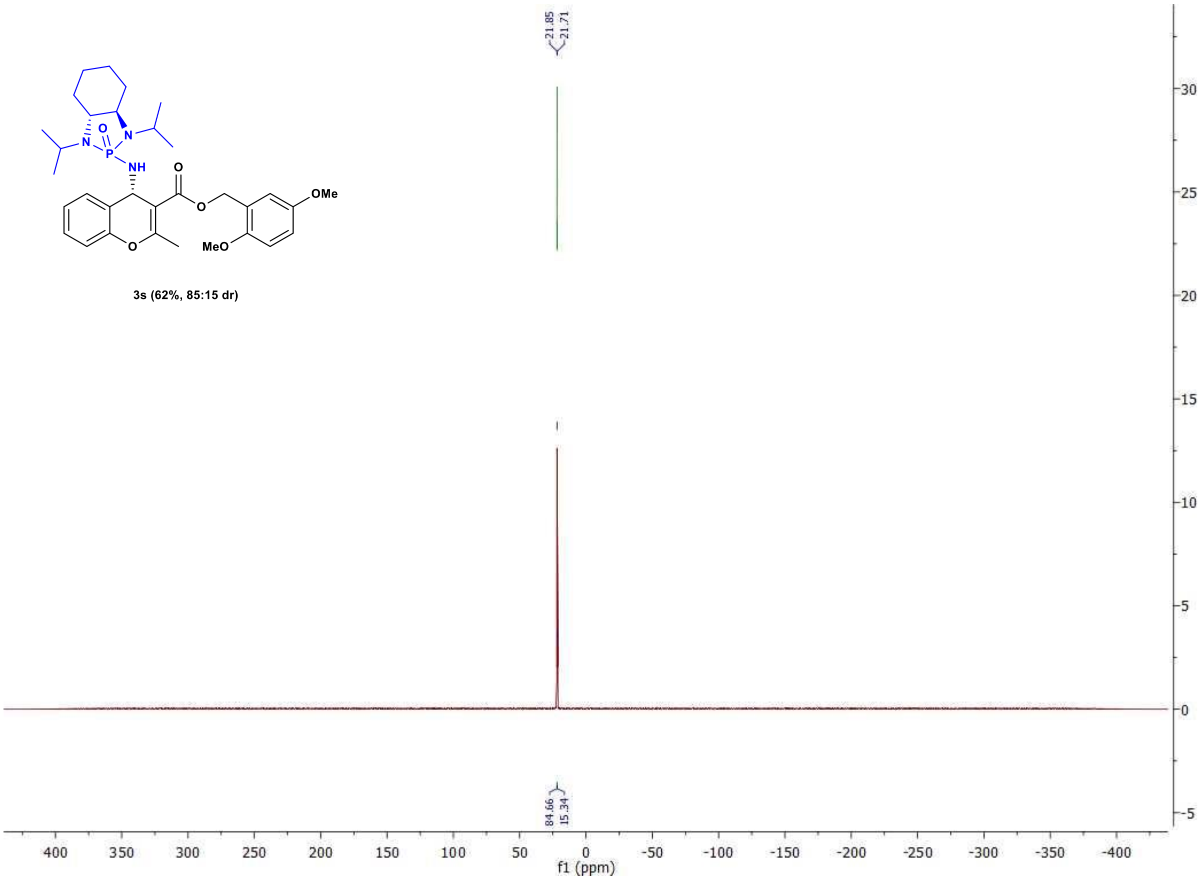

^{31}P NMR of Chromene **3s** (CD_3CN, 162 Hz)
